# A microbial therapy-mimicry assay shows how spatial resource dynamics control resistance escape

**DOI:** 10.1038/s41559-026-03178-z

**Published:** 2026-09-28

**Authors:** Nico Appold, Timon Citak, Auguste A. Palm, Jona Kayser

**Affiliations:** 1 Max-Planck-Zentrum für Physik und Medizin & Max Planck Institute for the Science of Light, Erlangen, Germany; 2 Department of Physics, Friedrich-Alexander-Universität Erlangen-Nürnberg, Erlangen, Germany; 3 Max Planck Institute for Infection Biology, Berlin, Germany; 4 Department of Biology, Chemistry, and Pharmacy, Freie Universität Berlin, Berlin, Germany

**Keywords:** Experimental evolution, Population dynamics

## Abstract

The evolution of therapy resistance in structured populations such as biofilms and solid tumours is shaped by emergent spatial organization, with profound consequences for evolution-based therapies. However, how treatment reshapes these patterns remains poorly understood. Here we show that intermittent treatment pulses transiently reconfigure the resource landscape, reorganize spatial growth zones and can enable resistant mutants to escape spatial confinement and drive therapy failure. We introduce a spatial evolution assay in which populations expand from single, genetically tailored yeast cells, enabling quantitative tracking of the full spatiotemporal trajectories of continually emerging resistant mutants under intermittent treatment. By integrating these observations with a mechanistically interpretable computational model in a real-to-sim-to-real loop, we identify a dynamic phase-transition-like boundary in schedule space that defines a candidate optimal balance between population control and sustained resistance confinement, which we test experimentally. Together, our results establish resource-mediated spatial confinement as a central organizing principle of resistance evolution and provide a mechanistic foundation for spatially informed, evolution-based therapies.

## Abstract (web summary)

This experimental and computational study asks how spatial structure and nutrient availability impact spread of resistant mutants and explores the impact of several treatment schedules to find a strategy that balances population control and resistance confinement.

## Main

Therapy resistance remains one of the biggest challenges in the treatment of pathological cellular populations, spanning bacterial infections, fungal pathogens and cancer^1–5^. Resistance-mediated therapy failure is driven by two tightly coupled processes: the continual de novo emergence of resistant mutants during population expansion and their subsequent amplification under treatment. In well-mixed, mean-field descriptions, these processes are often treated as separable, with mutations supplying resistant cells and therapy reshaping relative fitness. However, many cellular populations are neither well mixed nor exposed to spatially uniform environments. Biofilms, tissues and solid tumours are typically compact, mechanically crowded and subject to temporally varying spatial gradients of nutrients, oxygen and signalling molecules^6^. As a consequence, in structured populations, resistance evolution becomes intrinsically spatiotemporal: where a mutant arises within the population and when selective pressures change can be as important as the mutant phenotype.

Over the past two decades, a broad body of work has demonstrated that spatial population structure can fundamentally reshape evolution in dense, range-expanding populations through a variety of mechanisms, including changes in effective population size^7–13^ and collective cell dynamics^14–19^. In addition, advances in molecular manipulation techniques and spatial transcriptomics have begun to show the importance of spatial-evolutionary principles in an oncological context^20–23^. Together, these studies establish evolution in compact, growing populations as an emergent process in proliferating active matter that can become especially decisive when environmental conditions change^24^.

Therapy onset represents such a drastic environmental change and thus provides a natural conceptual bridge between spatial-evolutionary theory and the emerging paradigm of evolution-based cancer therapies. Rather than attempting to completely eradicate all cancer cells at the risk of accelerating resistance, evolution-based approaches deliberately exploit intrapopulation competition by making closed-loop treatment adjustments based on population-level observables and mathematical evolution models^25,26^. These adaptive therapies have shown promise in preclinical studies and early clinical trials^27–32^. Fixed-cycle intermittent schedules have been proposed as a minimal open-loop alternative that embodies a similar evolution-based rationale, but have so far shown limited success in clinical trials, potentially due to unmodelled heterogeneity in resistance phenotypes and schedule optimality^33–35^. In addition, simulations have demonstrated that local competition, spatial refugia and heterogeneity in resource and drug fields can qualitatively alter tumour evolution and therapy outcomes compared with mean-field predictions^35–41^.

**Fig. 1:**
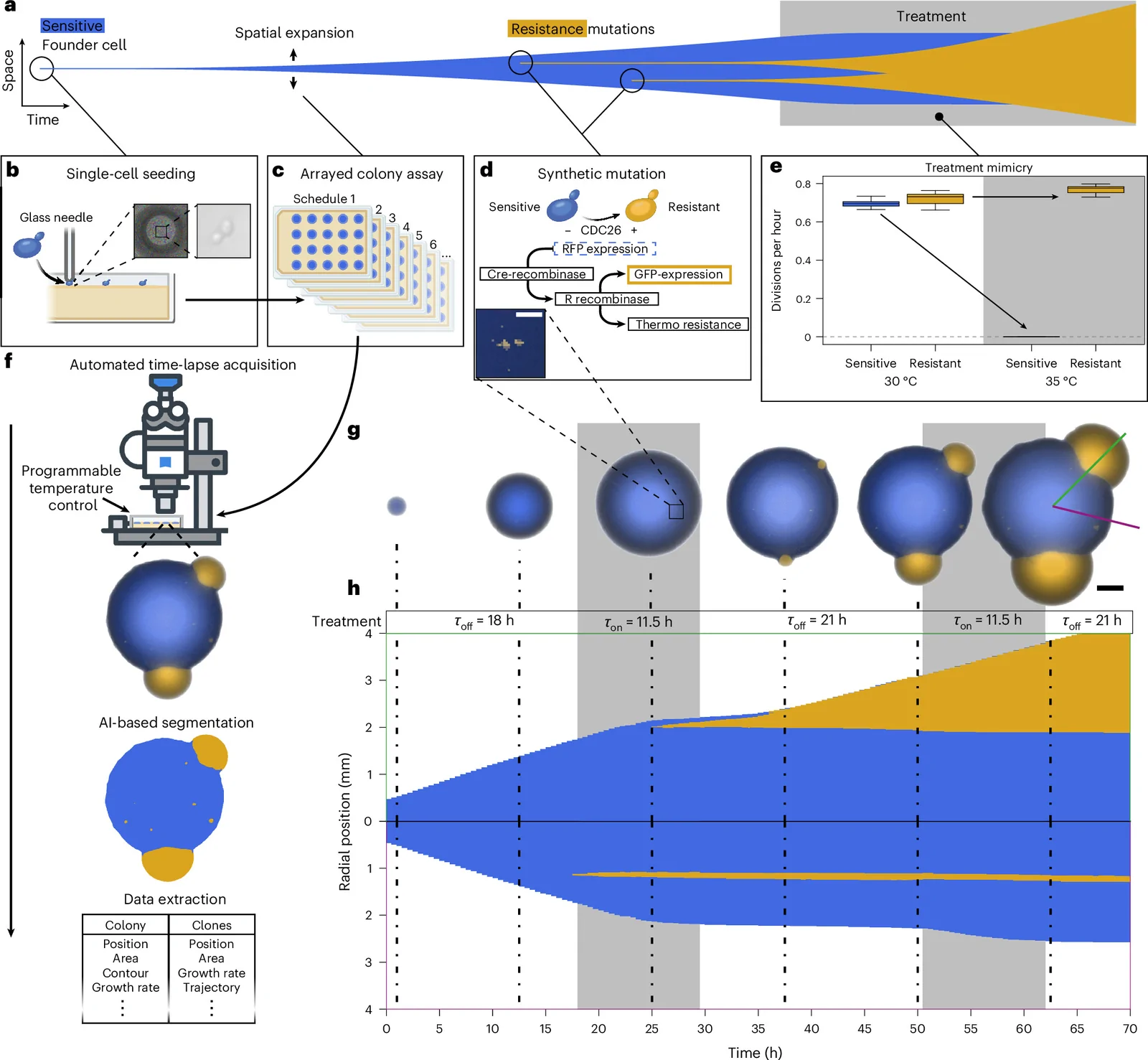
Experimental platform to study resistance under time-structured treatment. **a**, Schematic of resistance evolution and therapy failure in a spatially structured population. **b**, Single-cell seeding of sensitive cells. **c**, Colony formation and time-lapse imaging under variable treatment schedules. **d**, Synthetic mutation construct enabling stochastic, irreversible recombinase-mediated switching from thermosensitive to thermoresistant cells coupled to a fluorescence colour change (Extended Data Fig. 1). Inset: high-magnification image of resistant clones within a colony. Scale bar, 100 μm. **e**, Median growth rates of sensitive and resistant cells at 30 °C and 35 °C in liquid culture (also see Extended Data Fig. 1). Error bars indicate IQR, whiskers indicate 1.5× IQR; *n* = 9 per experiment and cell type, with two replicates for 30 °C. **f**, Image analysis pipeline from automated temperature control and time-lapse imaging to segmentation and colony- and clone-level data extraction. **g**, Representative intermittent therapy time-lapse. Imaging starts at *t* = 0 h (24 h after seeding). Resistant cells (yellow) expand while sensitive cells (blue) are arrested. Scale bar, 1 mm. **h**, Radial kymographs from the colony in **g** (coloured radials), showing escape (top) and confinement (bottom) of bulk-born resistant mutants. Grey shading indicates treatment. Dash-dotted lines indicate time points in **g**.

Together, these advances highlight a growing convergence between spatial-evolutionary theory and evolution-based therapy. Yet a quantitative, mechanistic understanding of how spatial heterogeneity, mutation supply and time-varying selection jointly shape evolutionary dynamics and therapy outcome is still missing. In particular, we lack experimentally tractable systems that integrate growth-driven range expansion, a continuous supply of de novo mutations and dynamically modulated treatment to test mechanistic hypotheses and validate in silico-derived therapy strategies (Fig. 1a).

Here we close the real-to-sim-to-real loop by combining a genetically tailored, high-throughput microbial therapy-mimicry assay with a mechanistically interpretable spatial digital twin to show that therapy-induced reconfiguration of the spatial resource landscape governs resistant-mutant dynamics, creating a sharp boundary between sensitive-dominated control and resistance-driven failure that defines a therapeutic sweet spot predicted in simulations and validated experimentally.

## Results

### A platform for tracking resistant mutants under time-modulated therapy

To quantitatively probe how de novo resistance emergence interacts with time-modulated, therapy-driven population dynamics in a spatially structured setting, we designed an experimental evolution assay initiated from individually placed, genetically tailored *Saccharomyces* *cerevisiae* cells (Fig. 1b). These founder cells expand into compact populations in a high-throughput arrayed colony assay (Fig. 1c).

**Extended Data Fig. 1:**
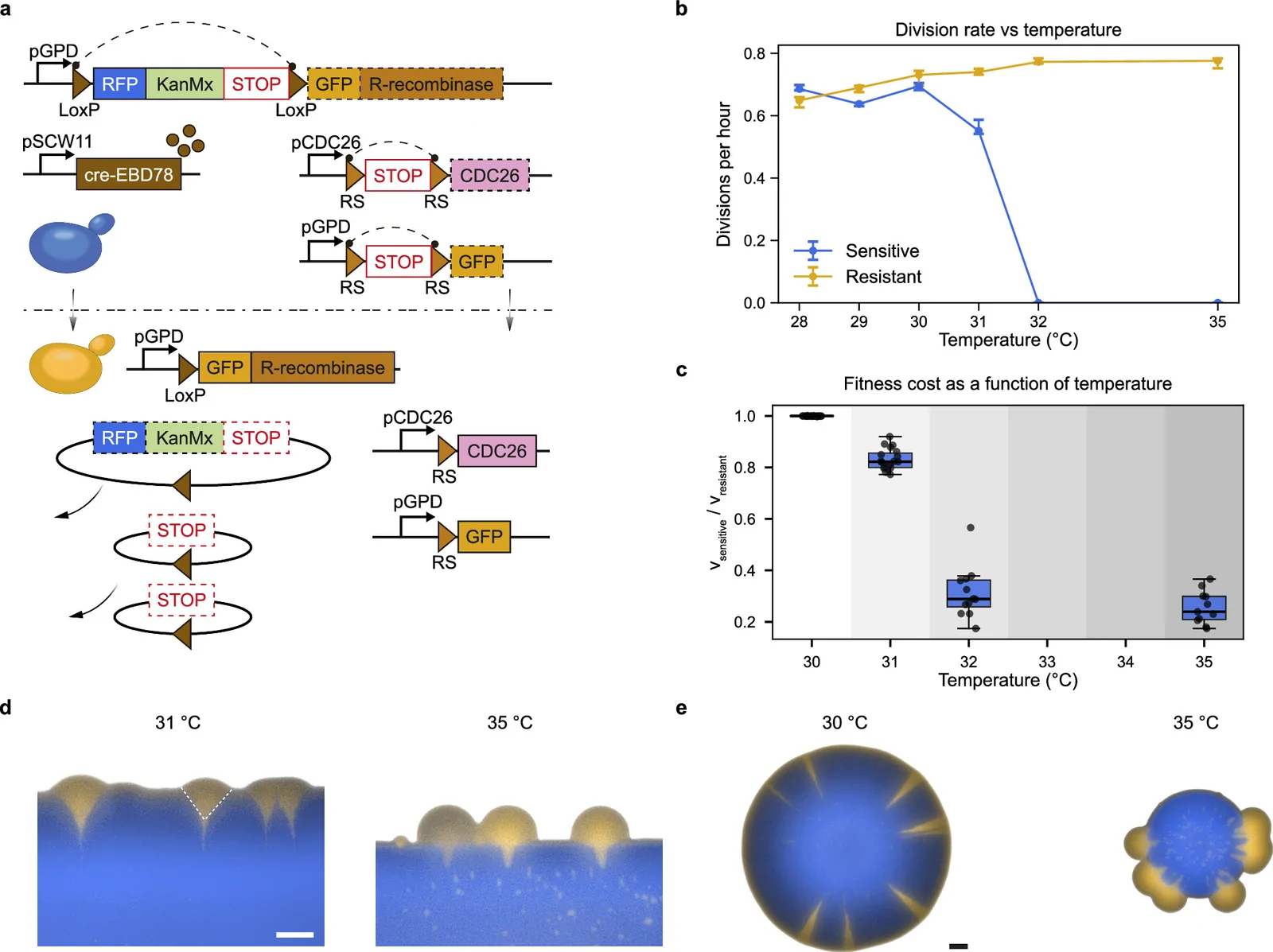
A hierarchical multi-recombinase system for synthetic thermo-resistance mutations. **a** Detailed schematic of the yNA16 genetic construct showing the synthetic mutation enabling the switch from thermo-sensitive to thermo-resistant cells via activation of an R-recombinase cascade through Cre-Lox recombination. **b** Median division rates of sensitive and resistant cells in liquid culture at the indicated temperatures. Whiskers show interquartile range. *n* = 9 per experiment and cell type, with two replicates for 30 °C. **c** Relative fitness of sensitive cells calculated from sector opening angles in linear colonies with random mutations at the indicated temperatures. Fitness is shown as the expansion velocity ratio *v*_sensitive_/*v*_resistant_. Points show individual angle measurements; boxes indicate the median and interquartile range. *n* = 24 for 30 °C, *n* = 19 for 31 °C, *n* = 12 for 32 °C, *n* = 11 for 35 °C. **d** Representative linear inoculation experiments at 31 °C and 35 °C with expanding resistant clone fronts; dashed lines indicate measured opening angles. **e** Representative radial mixed colonies containing resistant yNA16s cells diluted 1:1000 into susceptible yNA16 cells under no-treatment (30 °C) and treatment (35 °C) conditions. Linear and radial inoculations were grown at 30 °C for 24 h before being transferred at respective temperatures. **d-e** Scale bars, 1 mm.

To generate a continual supply of trackable resistant mutants, we engineered a stochastic and irreversible synthetic mutation system that couples resistance to a change in fluorescence colour via a hierarchical dual-recombinase switch (Fig. 1d and Extended Data Fig. 1a). Time-dependent treatment is implemented via programmable temperature shifts, allowing precise control over therapy onset, duration and pause (Fig. 1e and Extended Data Fig. 1b). In the unmutated state, cells are thermosensitive and proliferate at 30 °C but arrest at 35 °C, whereas mutated cells become thermoresistant and continue to proliferate at elevated temperature. Because this mutation is irreversible and fluorescently reported, resistant lineages can be identified and tracked throughout colony growth.

We combine this assay with multi-day time-lapse fluorescence microscopy and automated temperature control to record population histories for up to 160 h (>100 generations) (Fig. 1f). To quantitatively extract colony- and clone-level dynamics across the entire population history, we developed a custom image analysis pipeline combining automated acquisition with machine learning-based segmentation.

Figure 1g shows a representative time series under an intermittent schedule. To visualize spatiotemporal dynamics along the direction of expansion, we compute radial kymographs from selected colony radials (Fig. 1h; coloured radials in Fig. 1g). We observe two qualitatively distinct fates for resistant clones. Although some can permanently escape confinement as continually growing domes, most resistant clones remain confined within the population bulk.

Together, these observations establish a baseline phenomenology: in spatially structured, range-expanding populations, de novo resistant mutants are continually generated but are typically confined within the bulk. Therapy can alter this fate, enabling some bulk-born resistant clones to escape, whereas others remain suppressed. In the following sections, we investigate the mechanisms governing this confinement and release, and how they can be controlled by the timing of therapy.

### Pausing therapy spatially reconfines resistant mutants

**Fig. 2:**
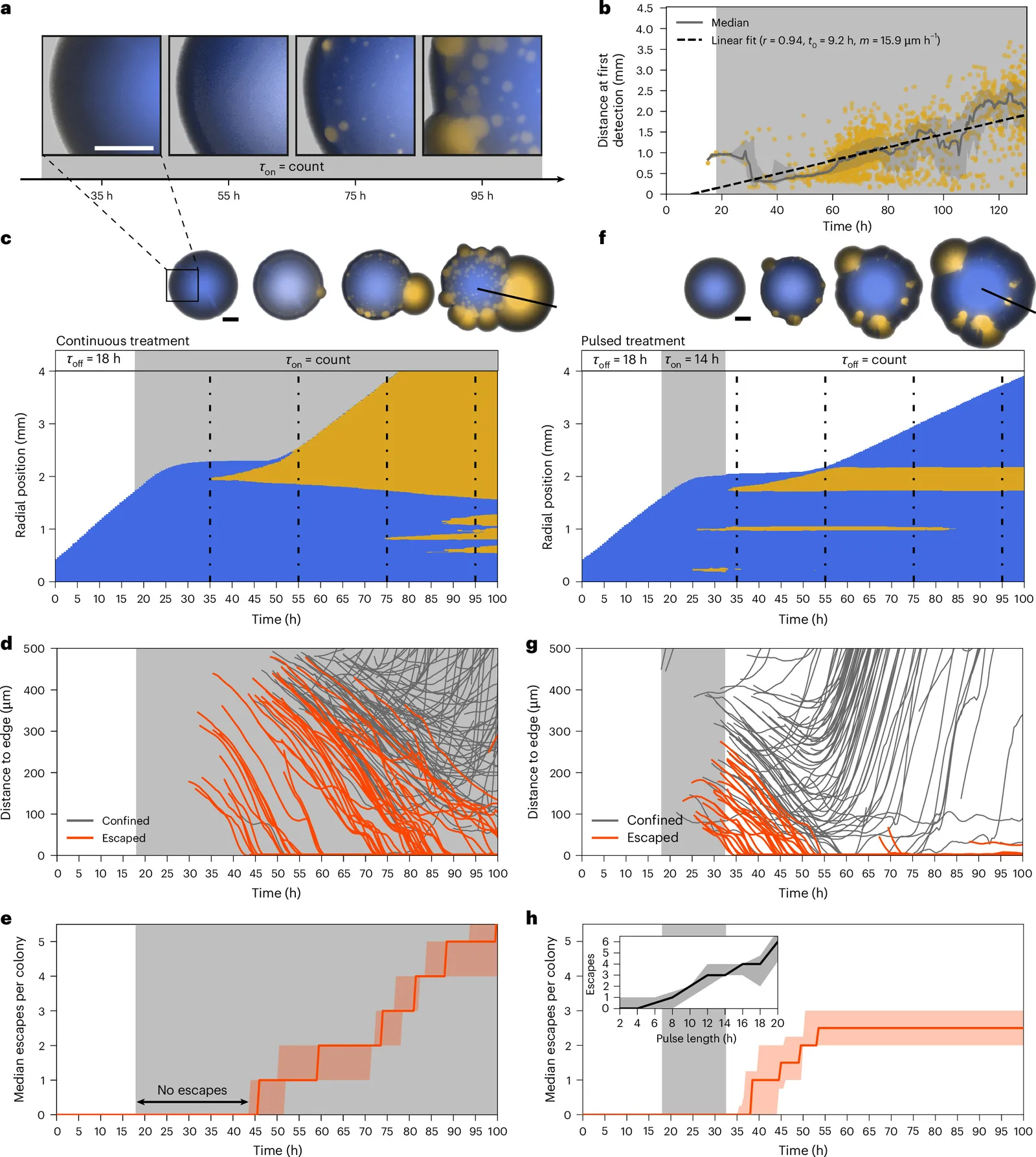
Confinement of resistant clones under pulsed therapy. **a**, High-magnification time-lapse images showing expansion of resistant clones during treatment. **b**, Distance of resistant clones to the colony front at first detection during CT (median ± IQR). **c**,**f**, Radial kymographs with corresponding time-lapse images for representative colonies under continuous (**c**) and pulsed (**f**) schedules. Dash-dotted lines mark shown time points. Scale bars, 1 mm. **d**,**g**, Trajectories of the front position of individual resistant clones over time for all colonies under continuous (**d**, *n* = 9 colonies) and pulsed (**g**, *n* = 12 colonies) treatment. **e**,**h**, Median cumulative number of escaped clones per colony under continuous (**e**) and pulsed (**h**) treatment. Inset: escape events per colony across pulse durations (median ± IQR, *n* in Supplementary Table 1 as ‘pulse test’). Grey shading indicates treatment in **a**–**h**.

To systematically study therapy-response dynamics, we first examined mutant emergence and expansion under continuous therapy (CT) (Fig. 2a). Colonies were expanded at 30 °C (therapy off) and then shifted to 35 °C (therapy on), where the temperature was held constant for the remainder of the experiment.

Despite the spatial homogeneity of the temperature shift, mutant expansion exhibits a pronounced spatiotemporal structure: the time at which a resistant mutant is first detected is strongly correlated with its distance from the colony front (Fig. 2b). Thus, resistant lineages do not become detectable throughout the colony simultaneously upon therapy onset, but rather appear progressively as a function of position, indicating that mutant growth is gated by a spatially varying growth potential within the colony.

Tracking individual mutant trajectories under CT reveals a characteristic approach towards the colony edge until permanent escape occurs (Fig. 2c,d). Notably, mutants originating further inward can sometimes remain confined when a more forward mutant lineage occupies the advancing front. At the colony level, these dynamics imply a finite time window after therapy onset during which no mutant has yet escaped; as therapy continues, this window closes and the cumulative number of escaped mutants increases steadily (Fig. 2e).

The existence of a delay between therapy onset and resistance escape suggests that a sufficiently short therapy pulse could be used to transiently suppress overall population expansion while still preventing mutants from reaching the colony edge. Moreover, the observation of local clonal interference raises the possibility that restoring sensitive population growth could act as an effective barrier at the front, enforcing reconfinement of resistant mutants.

To test this idea, we applied therapy as before but interrupted treatment after a pulse duration of *τ*_on_ = 14 h, allowing sensitive cells to resume growth (Fig. 2f). At the time of treatment interruption, no resistant mutant had yet escaped. Strikingly, this therapy pause indeed restored spatial confinement for many clones: mutants that had expanded during the pulse ultimately stalled and remained confined within the colony bulk without escaping (grey traces in Fig. 2g). Notably, the time at which these lineages became reconfined, as quantified by their closest approach to the colony front, often preceded the full recovery of sensitive population expansion, consistent with the re-establishment of an effective competitive barrier by sensitive cells. Nonetheless, because sensitive growth resumed only after a substantial regrowth lag, mutants residing sufficiently close to the front could continue advancing and escape for a limited period after therapy cessation (Fig. 2f–h).

Systematically varying the pulse duration shows that shortening treatment pulses strongly enhances spatial confinement (Fig. 2h, inset). In particular, for pulses shorter than *τ*_on_ < 6 h, resistance escape is almost completely suppressed, indicating that the maximum pulse length that reliably prevents escape is more restrictive than what would be inferred from CT trajectories alone, due to the additional time available for mutant advance during the post-pulse regrowth lag.

A possible explanation for these observations is that therapy reshapes the spatial resource landscape that naturally arises in many spatial population expansions^9,12,15^. More specifically, suppressing sensitive growth permits nutrients to redistribute from the colony periphery towards the interior, enabling bulk-born mutants to expand, whereas renewed sensitive growth restores resource depletion in the bulk and counteracts mutant advance. Nutrients are therefore a likely candidate for the hidden spatial field that gates mutant expansion, but directly measuring their spatiotemporal dynamics under time-modulated therapy is experimentally challenging. We therefore next introduce a mechanistically minimal digital twin that couples growth to a diffusing nutrient field in a spatial setting to test whether the observed release and reconfinement dynamics emerge naturally from this coupling.

### An in silico model couples spatial resource dynamics to resistance escape

**Fig. 3:**
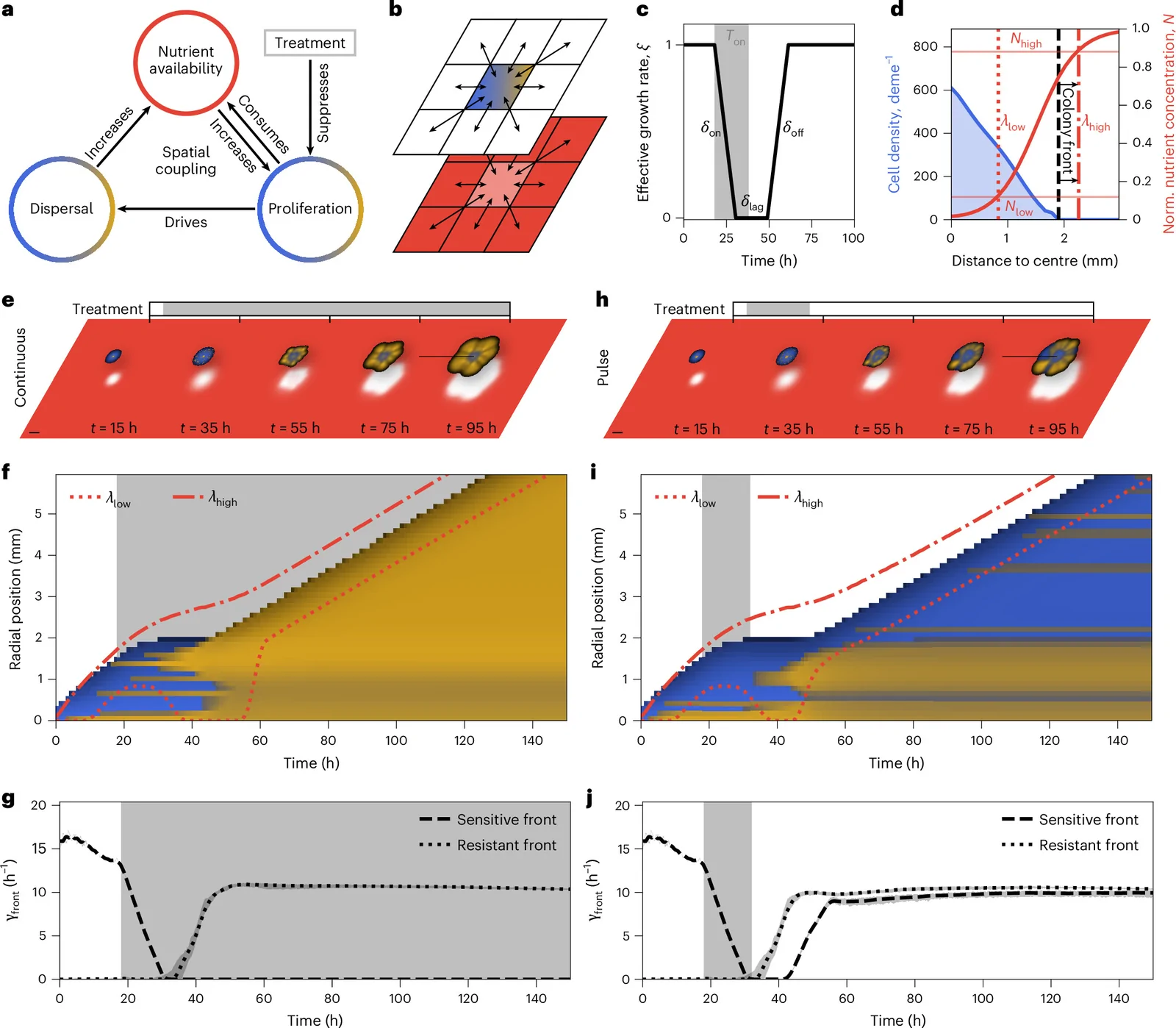
Simulation of spatial resource dynamics. **a**, Schematic of spatial couplings between nutrients, proliferation, dispersal and treatment. **b**, The simulation consists of sensitive (blue) and resistant (yellow) populations interacting with each other via an underlying nutrient landscape (red), organized on a 2D square lattice. **c**, Treatment modifies growth rates with delays, leading to an effective growth rate *ξ*. Grey shading indicates treatment. **d**, The nutrient dynamics in relation to the cell density can be visualized via a lower nutrient boundary *λ*_low_, defined by a set low nutrient threshold *N*_low_ and an upper nutrient boundary *λ*_high_, defined by the threshold *N*_high_ (Methods). **e**,**h**, Time-lapse of a CT (**e**) and a 14-h (**h**) pulse simulation run. The black line marks the position of the kymographs in **f** and **i**. Scale bar, 2 mm. Opacity indicates density. Sensitive and resistant cells are shown with gamma correction *γ* = 5 and *γ* = 2.5, respectively. **f**,**i**, Kymographs for the continuous dose and pulse treatments in **e** and **h**, respectively. The red lines show the nutrient dynamics as defined in **d**. Grey shading indicates treatment. The colours are displayed with gamma correction *γ* = 10 for all types. **g**,**j**, Median front cell growth speed *γ*_front_ of sensitive and resistant cells (*n* = 20) for continuous (**g**) and pulsed (**j**) treatment, calculated from the average type-specific number increase across all front demes. The shaded area indicates IQR. Grey shading indicates treatment.

To test whether therapy-induced release and reconfinement can emerge generically from coupling population growth to a spatial nutrient field, we constructed a stochastic simulation framework as a mechanistically minimal ‘digital twin’ in which nutrient dynamics are directly accessible (Fig. 3a). The model integrates nutrient-dependent proliferation, growth-coupled spreading and nutrient diffusion in two spatial dimensions. Growth and dispersal are spatially coupled: local proliferation drives outward spreading at the colony periphery, and this front motion continuously relocates the growth zone towards regions of higher nutrient availability similar to a travelling wave. As a result, population expansion is not merely enabled by nutrients, but also reshapes nutrient access by shifting where consumption occurs in space.

In short, space is discretized into a two-dimensional (2D) grid of demes that contain sensitive cells *S* and resistant cells *R*. Cells proliferate at a nutrient-dependent rate and undergo growth-driven local spreading (Fig. 3b). Resistant mutants arise stochastically during sensitive growth, such that mutation supply is coupled to proliferation as in our experiments. Therapy is implemented as a modulation of sensitive growth by a factor *ξ* ∈ [0, 1] that captures experimentally observed response kinetics, including an on-delay, a post-treatment lag and an off-delay (Fig. 3c). Importantly, competition is mediated solely through nutrient limitation (no explicit local carrying capacity), so that both cell types interact only indirectly via a shared nutrient field. Nutrients are modelled as a freely diffusing consumable resource that is depleted by local growth.

To visualize the shape of the nutrient gradient and its therapy-induced changes, we compute the position of two complementary nutrient thresholds: the low-threshold position *λ*_low_ and the high-threshold position *λ*_high_ (Fig. 3d). Whereas *λ*_low_ reports how far growth-permitting nutrients penetrate into the bulk, *λ*_high_ reports how tightly the leading edge remains coupled to the high-nutrient side of the gradient.

Using the model parameterized from independent growth, mutation and treatment-response measurements (Methods; Supplementary Fig. 1), we simulated population response to CT and to a finite pulse of *τ*_on_ = 14 h (Fig. 3e,h). Despite its minimal structure, the digital twin qualitatively reproduces the key spatiotemporal dynamics observed experimentally: resistant mutants originating in the bulk can become released and advance towards the colony edge under therapy, whereas pausing therapy can prevent escape and restore spatial confinement in a duration-dependent manner.

Because nutrients are explicitly modelled, we can directly link these behaviours to therapy-induced dynamics of the growth-permitting layer. Under CT, suppressed sensitive growth reduces nutrient consumption, allowing nutrients to penetrate inward and causing *λ*_low_ to move inward over time (Fig. 3f and Supplementary Fig. 2). This progressive expansion of the growth-permitting region releases bulk-born resistant mutants and ultimately enables them to reach and escape at the colony edge. Figure 3g shows the type-specific growth rate *γ*_front_ of cells at the front.

Under a finite pulse, by contrast, both the increase of *λ*_low_ and reduction in *γ*_front_ are transient: if therapy is halted sufficiently early for *γ*_front_ to recover, escape is reduced and the nutrient landscape relaxes towards its pretreatment state, restoring spatial confinement (Fig. 3h–j).

Together, Fig. 3 supports a simple mechanistic picture consistent with the experimental observations: spatial growth and nutrient consumption generate a nutrient gradient along the expansion direction, and growth-driven front advance continuously couples the colony edge to the high-nutrient side of this gradient. Therapy transiently weakens this coupling by suppressing sensitive growth, broadening the peripheral nutrient landscape and releasing bulk-born resistant mutants. Interrupting therapy can prevent escape by reforming a competitive barrier of sensitive cells at the colony edge and restore confinement by re-establishing nutrient depletion in the bulk. Note that the intentionally minimal nature of the model primarily serves as a mechanistically interpretable test of whether growth-nutrient coupling is sufficient to qualitatively capture the observed response classes. Any quantitative predictions therefore need to be interpreted cautiously, especially as we turn next to the question of therapy optimization.

### A phase-transition-like boundary defines a therapeutic sweet spot

Therapy optimization poses an intrinsic trade-off: suppressing sensitive growth slows overall colony expansion but increases the risk of resistant mutant escape; conversely, allowing sensitive growth helps to maintain spatial confinement, but comes at the cost of continued population expansion. Aiming to balance growth suppression with confinement naturally motivates a concatenation of therapy-on and therapy-off intervals, often referred to as intermittent therapy. However, the observed spatial couplings render a straightforward identification of optimal combinations of interval lengths *τ*_on_ and *τ*_off_ elusive.

**Fig. 4:**
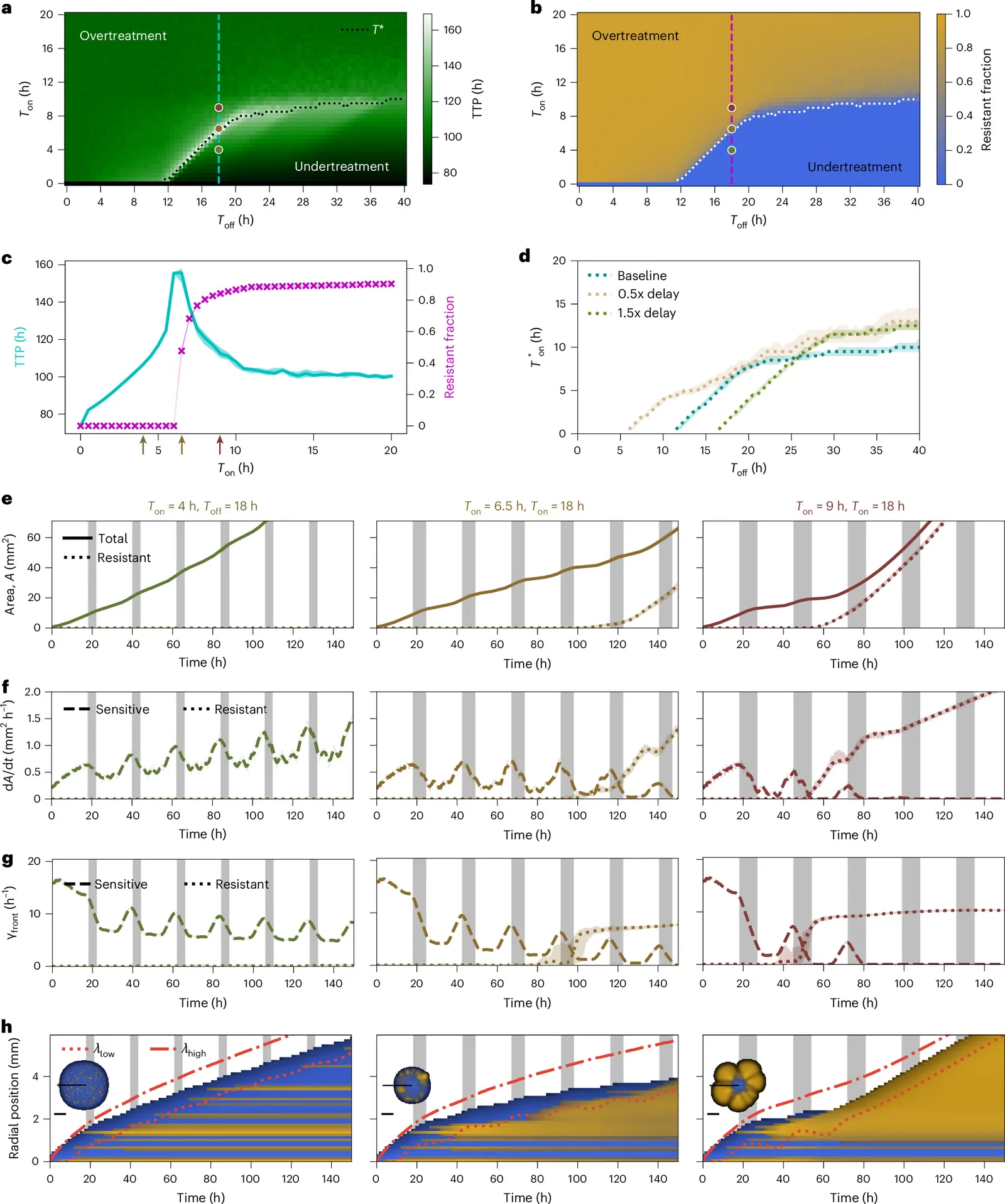
A dynamic phase-transition-like boundary in treatment space defines therapy outcome. **a**, Parameter sweep over combinations of *τ*_on_ and *τ*_off_, showing the median TTP (see main text) (*n* = 20 runs for each *τ*_on_, *τ*_off_ pair). Coloured dots mark the schedules shown in **e**–**h**. The turquoise dashed vertical line corresponds to the TTP profile shown in **c**. The dotted line shows τ* defined as the position of maximum TTP for each *τ*_off_ value. **b**, Median resistant fraction (*n* = 20) for the sweep shown in **a**. The dashed vertical line corresponds to the resistant fraction profile shown in **c**. **c**, Median TTP (turquoise) and resistant fraction profiles (pink crosses) together with their respective IQRs (*n* = 20) for the dashed vertical lines in **a** and **b**. The IQR of the full sweep is provided in Supplementary Fig. 3. **d**, Median τ* for different treatment delays. The shaded area indicates the IQR (*n* = 200 runs with parameters independently fitted from bootstrapped data). **e**–**g**, Median area *A* (**e**), median area growth rates d*A*/d*t* (**f**) and median front cell growth speed *γ*_front_ trajectories (**g**) for the indicated treatment schedules. The shaded area is the IQR (*n* = 20). Grey shading indicates treatment. The corresponding NT and CT baseline plots as well as the resistant fraction plots are shown in Supplementary Figs. 5 and 6. **h**, Kymograph together with the nutrient dynamics indicated by the red lines for one example angle and simulation run of the above schedules. The colony image shows the entire example colony at 120 h. Scale bars, 2 mm. The black line shows the position of the kymograph in the colony. Grey shading indicates treatment. In the colony images sensitive and resistant cells are shown with gamma correction *γ* = 5 and *γ* = 2.5, respectively, whereas in the kymograph colours are shown with gamma correction *γ* = 10 for all types.

We used the digital twin to scan a large space of schedules **τ** = (*τ*_on_, *τ*_off_) (Fig. 4a,b and Supplementary Fig. 3). We quantify schedule performance by the time to progression (TTP), defined as the time required for colony area to progress above a predefined threshold (Methods). Across schedule space, TTP exhibits a pronounced ridge of optimal schedules, τ*, separating two modes of early progression (Fig. 4a). On one side, progression is sensitive-dominated (undertreatment); on the other, resistant-dominated (overtreatment), with a sharp switch in resistant fraction near τ* (Fig. 4b). Thus, τ* marks a narrow line of ‘sweet spots’ that maximizes delay while maintaining confinement. A slice through schedule space at *τ*_off_ = 18 h shows the shape and co-localization of the transitions in resistance fraction and TTP (Fig. 4c). In general, the position of the τ*-boundary depends on the extent of therapy-response delays, whereas variability remains small within the uncertainty of parameter fitting (Fig. 4d and Supplementary Fig. 4).

To connect this macroscopic optimum to within-cycle dynamics, we examined three schedules spanning the optimum (Fig. 4e–h; *τ*_on_ = 4 h, 6.5 h, 9 h; *τ*_off_ = 18 h). For short pulses, *γ*_front_ rebounds during each pause, rapidly restoring a sensitive rim at the edge. This reinstates bulk depletion and allows *λ*_low_ to retreat close to its prepulse value, rendering consecutive cycles approximately independent. For ${\tau }_{{\rm{on}}} < {\tau }_{{\rm{on}}}^{* }$, this leads to an almost constant cycle-averaged growth. Beyond ${\tau }_{{\rm{on}}}^{* }$, by contrast, therapy depresses *γ*_front_ strongly enough that front recovery during *τ*_off_ is incomplete. Because nutrient influx is set by sensitive cell front expansion, incomplete rebound of *γ*_front_ also causes a cycle-to-cycle drift in *λ*_low_. This ratchet-like loss of peripheral growth progressively weakens confinement until it can no longer be maintained, resulting in escape and rapid expansion of resistant clones (Fig. 4g,h). Consistent with this picture, the linear increase of τ* for intermediate pauses follows from therapy-response kinetics, whereas for long pauses the boundary is set by single-pulse escape and for very short pauses by the post-treatment regrowth lag.

**Extended Data Fig. 2:**
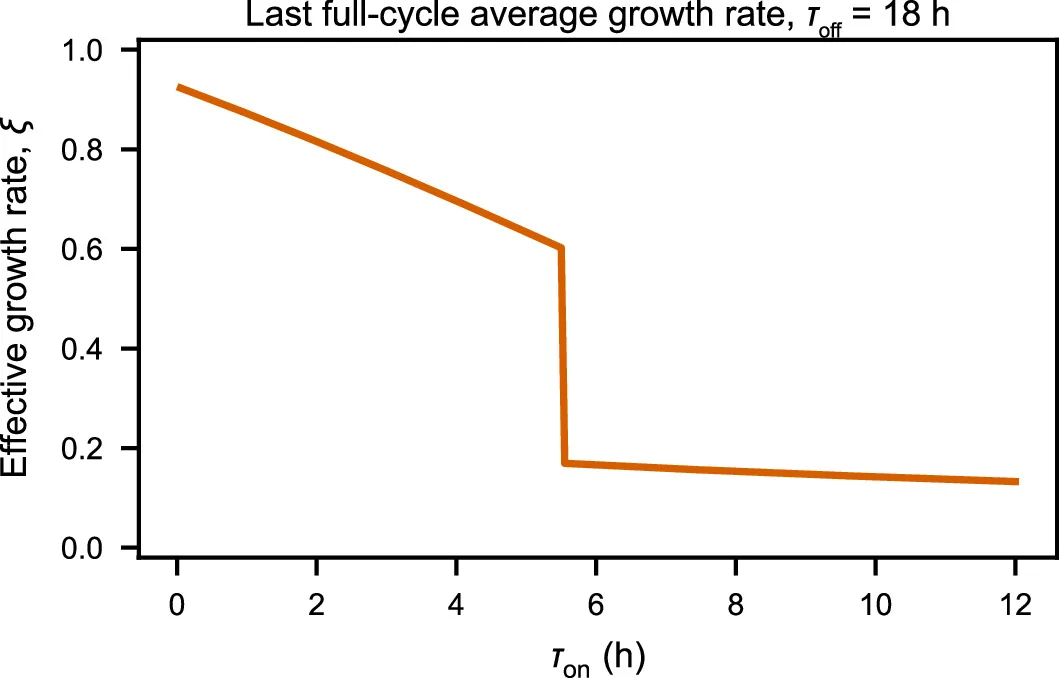
Schedule-dependent transition in the effective growth rate. Average effective growth rate of the last full treatment cycle (averaged over the duration of one cycle) along the *τ*_off_ = 18 h line. The values are calculated for long simulation times (≈ 625 days).

**Extended Data Fig. 3:**
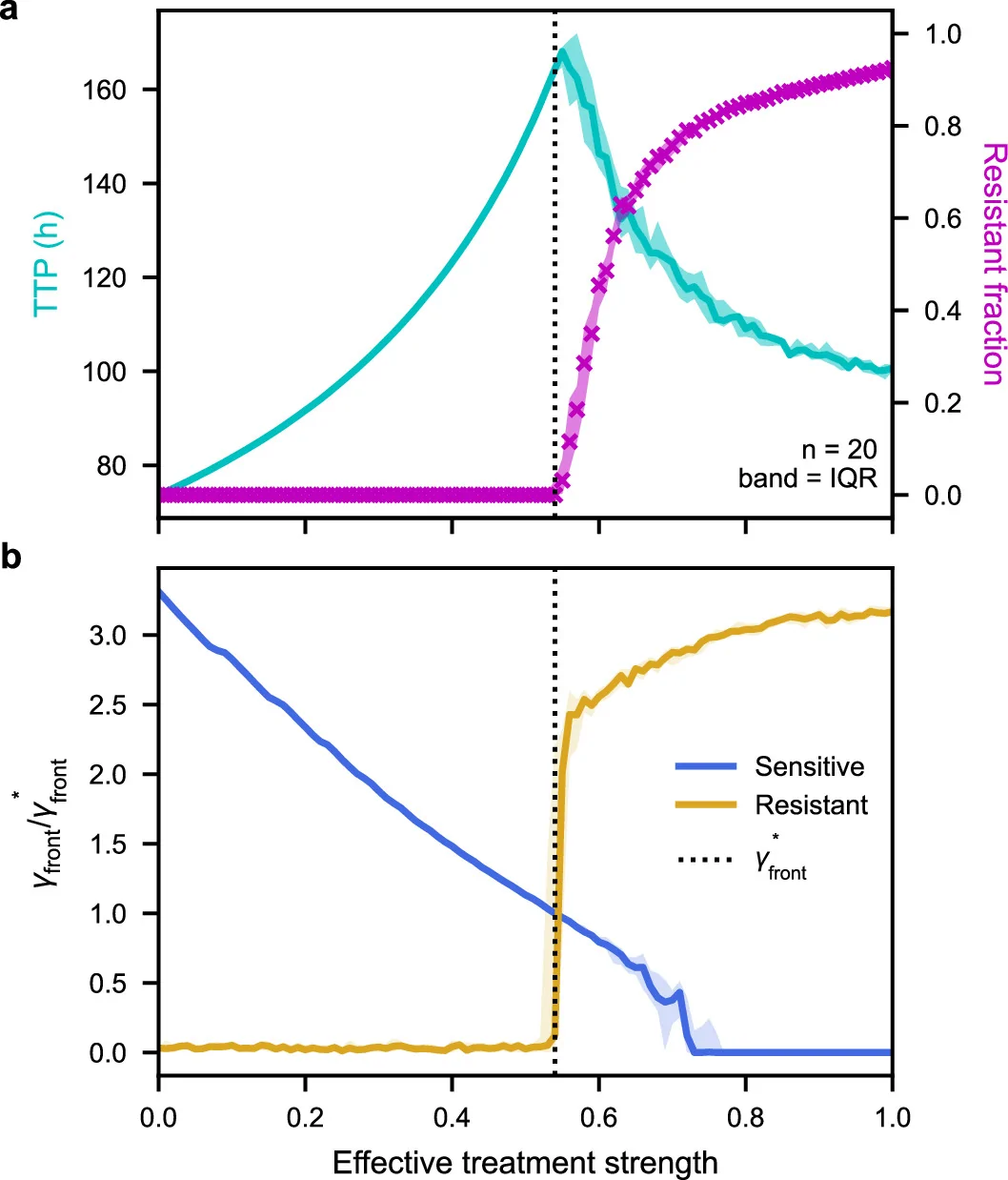
Transition in a schedule-independent constant dose scenario. **a** Median TTP and resistant fraction for a sweep performed over continuous therapy with different treatment strenghts (*n* = 20 runs for each treatment strength). The shaded area shows the IQR. **b** Median *γ*_front_ at TTP scaled by ${\gamma }_{{\rm{front}}}^{* }=3.37$ 1/h for both cell types (*n* = 20 runs for each treatment strength). The shaded area shows the IQR. The dotted line (effective treatment strength at ${\gamma }_{{\rm{front}}}^{* }$ is 0.54) is defined as the first point where the resistant *γ*_front_ deviates from the baseline, indicating escape.

**Extended Data Fig. 4:**
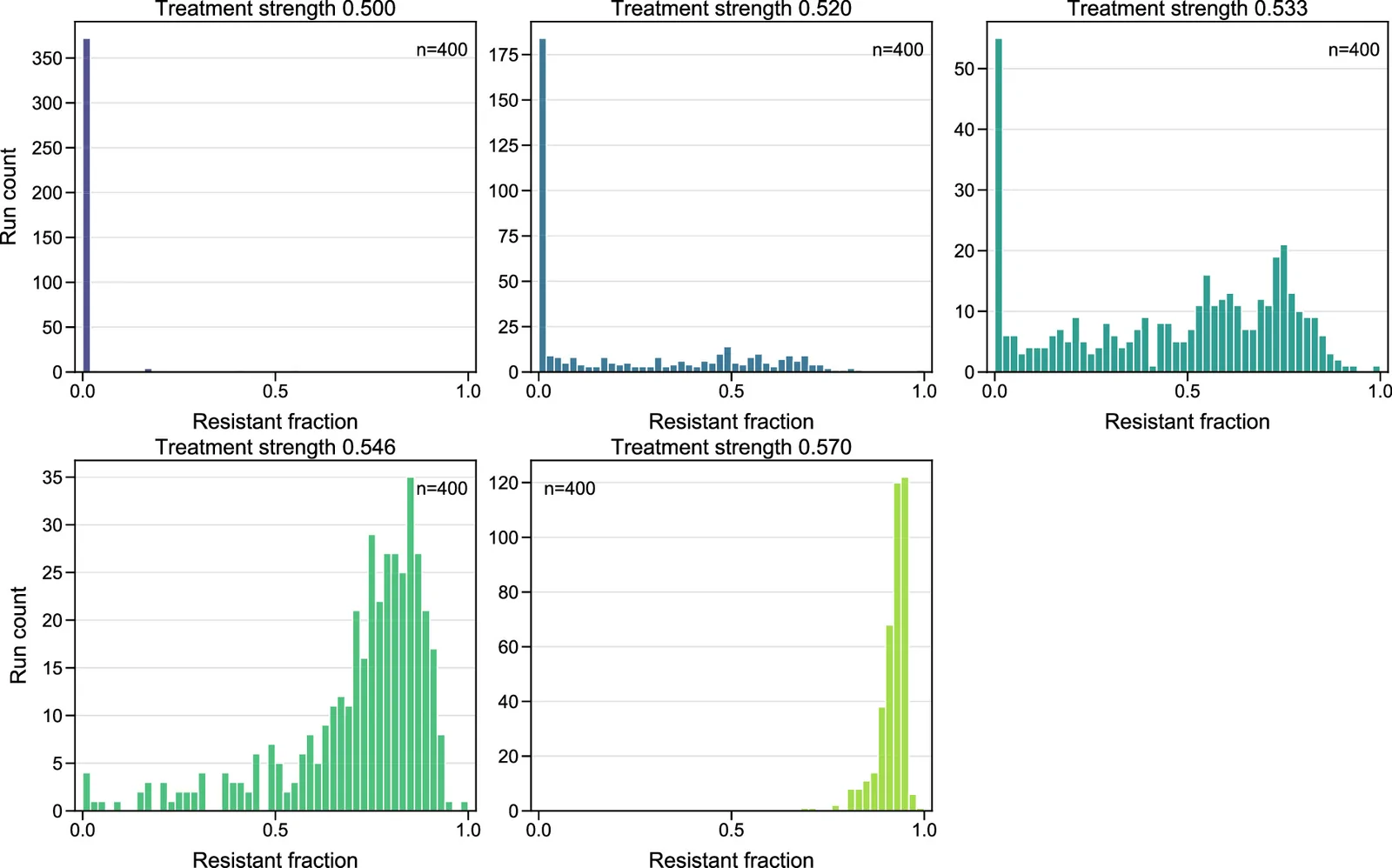
Bimodality analysis of the schedule-independent transition. Resistant fraction distributions for simulations with long simulation time (≈ 500 h). It shows continuous therapy simulations with two doses below, one close to, and two above the transition point. All distributions have a replicate number of *n* = 400.

**Extended Data Fig. 5:**
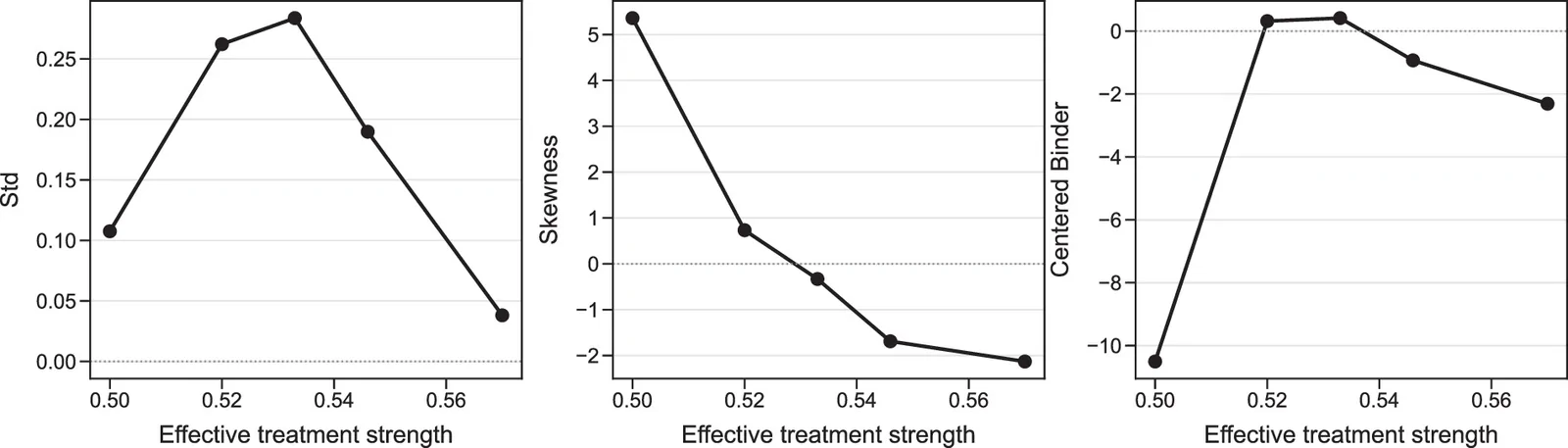
Bifurcation analysis of the schedule-independent transition. Std, skewness and centered Binder cumulant for the long simulation time continuous therapy runs at different treatment strengths. Each dot is calculated from the distribution (*n* = 400) of the resistant fraction at the endpoint of the simulation.

The sharp switch between a history-independent (approximately Markovian) regime and a history-dependent (non-Markovian) regime can be viewed as a dynamic phase-transition-like boundary in schedule space (Extended Data Fig. 2 shows this along one *τ*_off_ in the long-term cycle-averaged growth rate), analogous to dynamic transitions described previously for population extinction dynamics and conversional meltdown transitions^13,42,43^. Unlike the shift between equilibrium states in canonical thermodynamic phase transitions, these transitions separate distinct classes of dynamic trajectories. A detailed analysis of our scenario, using the resistant fraction as trajectory-level order-parameter-like observable, is provided in Supplementary Information. In short, the observed transition between sensitive-dominated or resistance-dominated trajectories results from a combination of two superimposed mechanisms, one schedule-dependent and one schedule-independent (Extended Data Figs. 2–5).

Finally, we do not expect the simplified digital twin to quantitatively reproduce all experimental details. Quantitative predictions from the sweep should therefore be interpreted as rough guides. Nevertheless, the underlying mechanism is generic and should remain relevant in more complex experimental settings.

### Experimental test of intermittent therapy

**Fig. 5:**
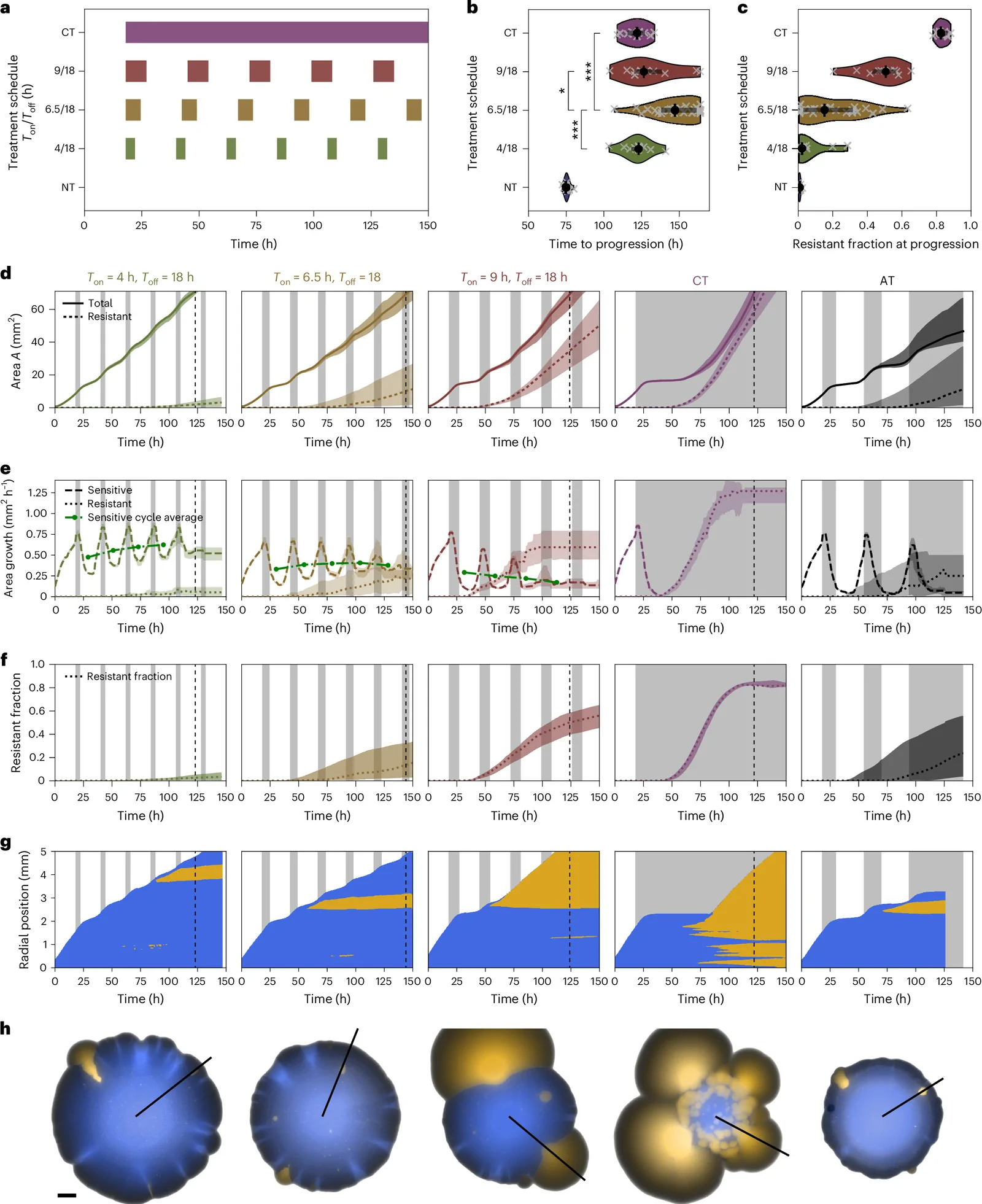
Experiments support delayed progression found in simulated treatment schedules. **a**, Experimentally tested schedules and corresponding *τ*_on_ intervals. **b**, Violin plots of TTP, defined as the first time point at which colony area reached ≥71 mm^2^, for indicated treatment schedules. Grey crosses represent individual colonies, markers indicate medians and bars indicate IQRs. Statistical comparisons were performed using one-sided Mann–Whitney *U*-tests testing whether TTP under the 6.5 h/18 h strategy was greater than under the respective comparator strategy, followed by Holm correction for several comparisons. Strategy-to-strategy comparisons around the optimum are indicated in the plot (6.5 h/18 h versus CT: ΔTTP 25.5 h, 95% CI 16–40.5 h, *P* = 0.00036; versus 9 h/18 h: ΔTTP 21 h, 95% CI 3–35.5 h, *P* = 0.01004; versus 4 h/18 h: ΔTTP 24.5 h, 95% CI 17–38.25 h, *P* = 0.00036). *P ≤ 0.05, **P ≤ 0.01, ***P ≤ 0.001. **c**, Resistant cell fraction at TTP (median, IQR). **d**, Median total and resistant colony areas for indicated intermittent schedules, CT and AT. Shaded areas indicate IQR. Dashed black lines indicate median TTP. **e**, Median growth dynamics of sensitive and resistant populations, including cycle-averaged sensitive growth. Shaded areas indicate IQR. Dashed black lines indicate median TTP. **f**, Median temporal evolution of the resistant fraction. Shaded areas indicate IQR. Dashed black lines indicate median TTP. **g**, Radial kymographs of representative colonies for each indicated treatment strategy. **h**, Images at 120 h corresponding to **g**; black radials mark the sampled angle. Scale bar, 1 mm. Grey shading denotes treatment periods. Sample sizes in **b**–**f** were *n* = 25 colonies for 6.5 h/18 h, *n* = 9 for CT, *n* = 10 for 9 h/18 h, *n* = 10 for 4 h/18 h, *n* = 8 for AT and *n* = 13 for NT baseline. See Supplementary Fig. 7 for NT.

Guided by the digital twin, we tested the observed principles empirically by applying the same set of schedules (*τ*_off_ = 18 h; *τ*_on_ = 4 h, 6.5 h, 9 h) in experiments, in addition to no-treatment (NT) and CT references (Fig. 5a).

Comparing overall therapy performance by measuring the TTP and resistant fraction, we find a characteristic local optimum similar to that observed in the digital twin (Fig. 5b,c). Although the optimum is significantly better than the adjacent strategies and the resistant fraction appears to become bimodal at the transition, the limited resolution and statistical power of the experimental data curtail any direct conclusions on boundary sharpness. TTP initially increases with longer treatment duration but then peaks at *τ*_on_ = 6.5 h (Fig. 5b). This peak also correlates with a sudden rise in mutant abundance (Fig. 5c).

Measuring how the areas of sensitive and resistant cell populations change over time, analogous to the analysis of simulation data in Fig. 4, we find a behaviour that qualitatively matches that observed in the digital twin (Fig. 5d–g). For short treatment durations (*τ*_on_ = 4 h), therapy-induced growth rate reductions of sensitive cells are balanced by a rebound of growth during therapy pauses, resulting in a slowly increasing cycle-averaged growth rate and strong confinement of resistant mutants (Fig. 5e,f). For *τ*_on_ = 6.5 h, the cycle-averaged growth rate remains almost constant throughout the experiment, paired with a noticeable but still small increase in resistance fraction. Intriguingly, with *τ*_on_ = 9 h, experiments indeed exhibit the cumulative decrease in sensitive cell growth and the surge in mutant escapes that is characteristic of the resistance-dominated region of schedule space (Fig. 5e,f; *τ*_on_ = 9 h).

Although these empirical results only cover a very small, albeit critical, part of the therapy space, they support the model-predicted therapeutic sweet spot at the transition from sensitive-dominated to resistance-dominated outcomes in the tested region of schedule space (Fig. 5h). Although the transition appears more gradual in experiments than in simulations, this observation is a direct demonstration that the intrinsic coupling between spatial population structure and a therapy-modulated nutrient landscape can be exploited for therapy optimization while concomitantly making it challenging to hit the optimal balance.

**Extended Data Fig. 6:**
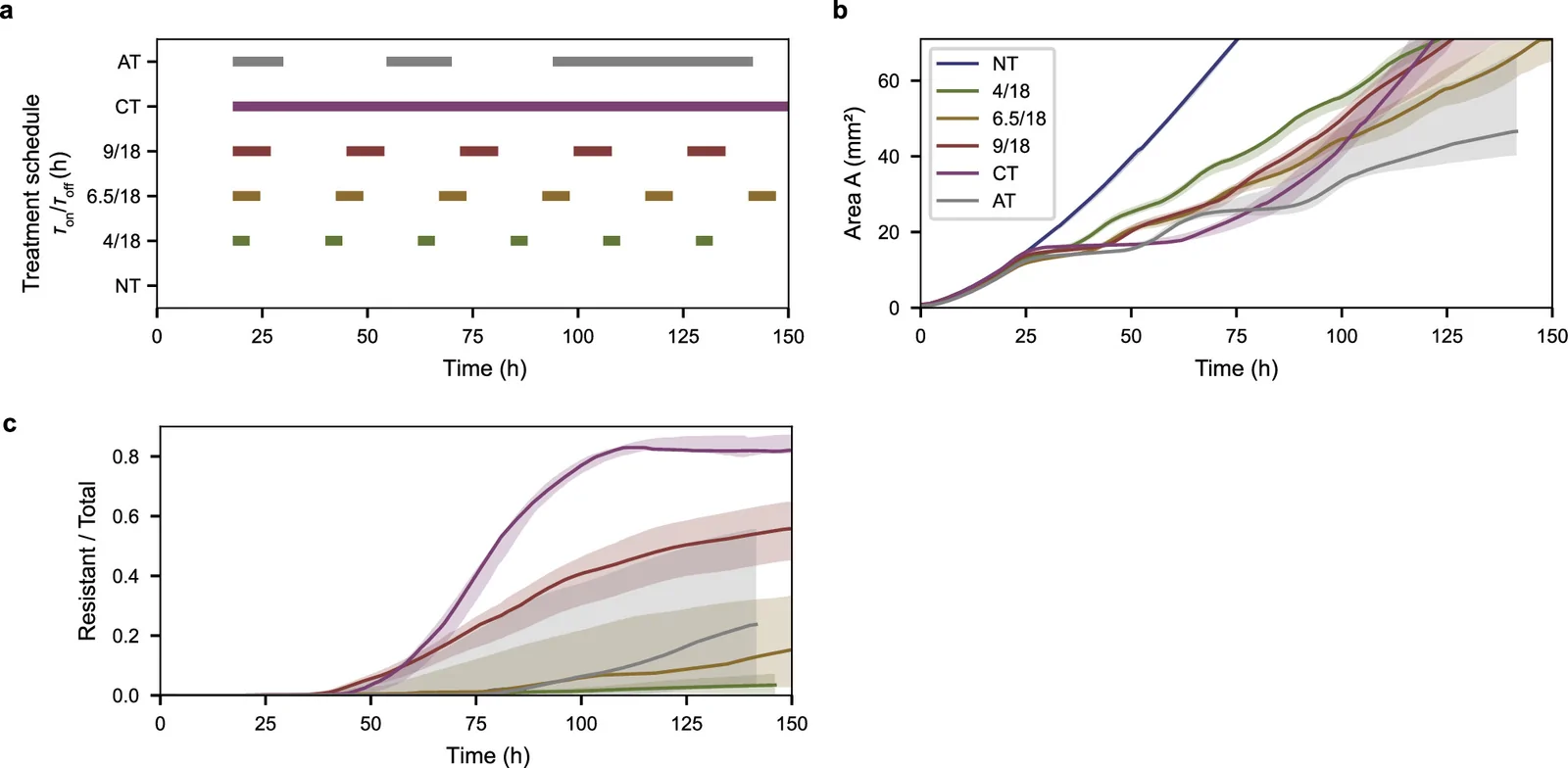
Direct schedule comparisons. **a** Treatment schedules used for adaptive therapy (AT), continuous therapy (CT), 9 h/18 h (*τ*_on_/*τ*_off_), 6.5 h/18 h, 4 h/ 18h and no treatment (NT). **b** Total population area over time under the indicated treatment schedules. Solid lines indicate the median size; shaded areas denote the interquartile range. **c** Fraction of resistant area over time for the indicated treatment schedules. Solid lines indicate the median; shaded areas denote the interquartile range. **a-c** Sample sizes were *n* = 25 colonies for 6.5 h/18 h, *n* = 9 for CT, *n* = 10 for 9 h/18 h, *n* = 10 for 4 h/18 h, *n* = 8 for AT, and *n* = 13 for NT.

However, these data should not be interpreted as confirmation of a globally sharp and symmetric boundary across all combinations of *τ*_on_ and *τ*_off_. For example, experiments with varying pause duration suggest an inter-cycle feedback between treatment effect and the preceding recovery phase that is not captured by the minimal digital twin (Supplementary Fig. 8). This feedback results in stabilized cycle-averaged growth rate profiles across different *τ*_off_ and, consequently, a wider TTP optimum along this axis. Thus, the relevant control variable may not be only the prescribed pair (*τ*_on_, *τ*_off_), but the realized growth state of the system as it enters each treatment pulse. Motivated by this observation, we asked whether the spatial-confinement mechanism could be translated into a simple closed-loop adaptive treatment heuristic based on measured growth rebound rather than on fixed schedule intervals alone. As a proof-of-principle heuristic, we performed an adaptive therapy (AT) experiment in which we continuously monitored colony expansion to adaptively control treatment intervals in real time. The aim of this strategy was to briefly halt proliferation and to then allow sensitive growth to rebound before the next treatment pulse (Fig. 5d–h, AT). Indeed, AT resulted in a slower overall growth than observed for any fixed-cycle schedule, typically delaying progression beyond the experimental runtime (see Extended Data Fig. 6 for a direct schedule comparison). At the same time, resistant-population dynamics were more variable than under any fixed schedule. Together, these results suggest that growth rebound may serve as an important and accessible state variable for adaptive scheduling. Within the scope of this work, however, this finding should be viewed as a proof of principle and as a starting point for future studies that optimize adaptive control policies in spatially structured populations.

## Discussion

This work shows that in structured populations, resource fluxes are a prime determinant of spatial growth patterns. Therapy can transiently disrupt these patterns, resulting in the escape of previously confined resistant mutants from the population bulk.

To systematically study the processes that govern these dynamics, this work introduces a therapy-mimicry assay based on a genetically tailored yeast model. Pairing empirical results with an experiment-parameterized digital twin in a real-to-sim-to-real approach, we probe a large therapy design space, identify an intermittent therapy schedule that optimally balances population growth and resistance confinement, and then test this in silico-derived strategy in experiments.

Three main conceptual insights emerge from this study. First, in populations undergoing resource-limited range expansion, therapy-induced halt of peripheral growth intrinsically results in a redistribution of resources towards the deprived bulk, which can release previously confined resistant mutants from resource-mediated confinement. Second, strategically pausing treatment can be leveraged as a general control principle by re-establishing resource-mediated confinement via rebounding sensitive cell growth at the population front. Third, small changes in treatment duration can cause dramatic changes in therapy outcome by shifting the system from an approximately Markovian to a non-Markovian response class, in which sensitive growth at the front progressively drifts below the threshold required to maintain confinement. The resulting dynamic phase-transition-like boundary defines a narrow therapeutic sweet spot whose location is governed by treatment-response kinetics.

A broad collection of previous work has shown how emergent spatial and physical phenomena reshape the evolutionary dynamics at the expanding front of these systems^9,11,12,15–17,37,38^. Our work builds on these insights by showing that fluctuating fitness landscapes, with modulated therapy being an extreme example, can transiently shift growth towards the mutant-rich population bulk, drastically increasing the genetic diversity accessible to evolution. Importantly, strategically controlling intrapopulation competition is foundational to the new paradigm of evolution-based tumour therapies^25,28,39,40^. In this context, our findings offer a new perspective on how the inherent resource gradients in solid tumours may be optimally exploited for improved therapy design. For example, using growth rebound as a clinically accessible measure for confinement potential in addition to overall burden might offer valuable guidance for treatment decisions. In addition, poorly vascularized tumour types, often associated with steep gradients and resource-depleted core regions, appear as ideal candidates for evolution-based therapies. Complementary treatments targeting angiogenesis, such as vascular endothelial growth factor (VEGF) inhibitors, may further enhance the effectiveness of such therapy strategies. In addition, our results may offer an additional rationale explaining why recent clinical trials of intermittent therapy did not improve progression-free survival in patients, despite strong preclinical evidence^34,35^. Finding the narrow therapeutic sweet spot is inherently challenging because of the biphasic nature of the therapy space whereas the commonly applied lead-in phase may eradicate it altogether.

**Extended Data Fig. 7:**
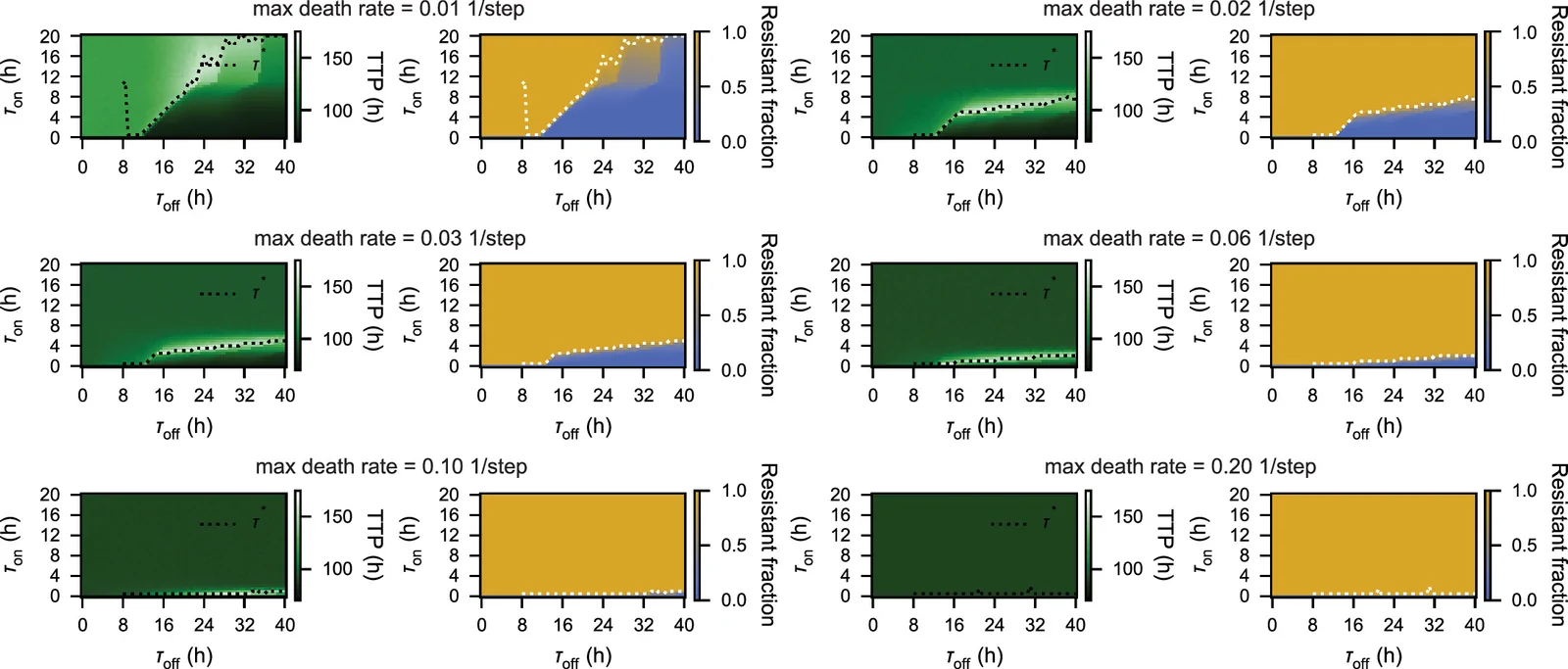
Parameter sweeps for cytotoxic treatment. Parameter sweeps for cytotoxic treatment of different strengths. Each pixel is the median of 20 replicates.

**Extended Data Fig. 8:**
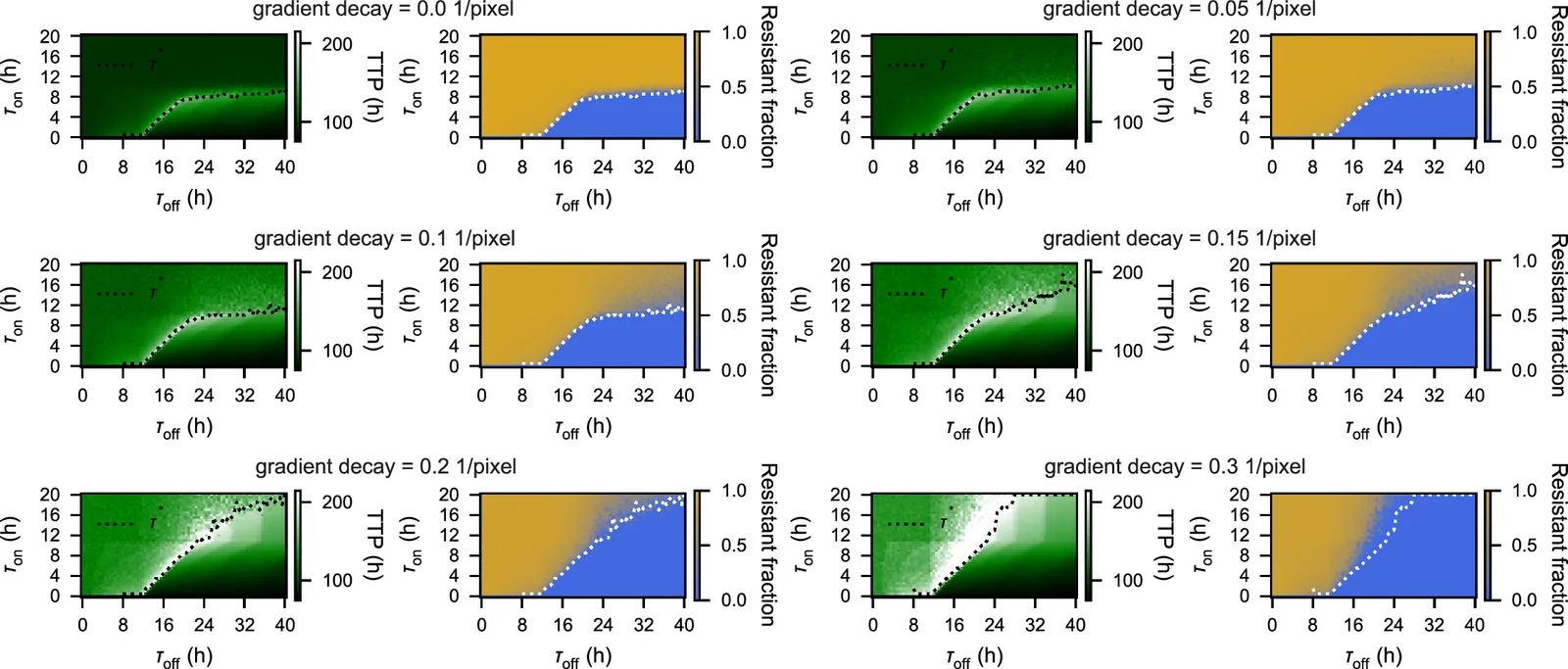
Parameter sweeps for spatial treatment gradients. Parameter sweeps for spatial treatment application of different decays. The treatment is strongest at the front of the colony and decays inward exponentially with the given decay factor. Each pixel is the median of 20 replicates.

Although the basic ingredients of the mechanism identified here—spatial structure, local competition, resource gradients and treatment-modulated growth—are present in many dense cellular populations, our assay and simulations are deliberately reductionist. Thus, the possibility that similar confinement-and-release dynamics arise in bacterial biofilms, poorly vascularized solid tumours or other structured populations should be interpreted as a mechanistic hypothesis. In more complex settings, features such as three-dimensional (3D) architecture, active migration, extracellular matrix, immune and stromal interactions, vascular structure and spatially variable drug penetration may alter resistance evolution and therapy response. Moreover, many antibiotic and anticancer therapies induce cell death rather than the reversible growth arrest studied here. It will therefore be essential to test resource-mediated spatial confinement and schedule-derived control strategies in more realistic model systems, such as 3D cancer organoids or matrix-producing biofilms, before drawing clinical conclusions. As an initial computational test of these limitations, we extended the digital twin to include therapy-induced sensitive cell death (Extended Data Fig. 7). The qualitative confinement boundary persisted in these cytotoxic simulations but shifted towards shorter treatment-on times as killing increased, consistent with the expectation that loss of sensitive cells delays re-establishment of a confining front. In the digital twin, we also tested spatially graded treatment fields in which treatment strength was highest near the colony periphery and decayed towards the population bulk (Extended Data Fig. 8). Across the tested gradients, the qualitative sweet spot remained present with steeper gradients facilitating confinement. These simulations do not constitute validation in tumour-like or biofilm-like systems, but they provide sensitivity analyses for two key assumptions of the yeast assay and emphasize that the quantitative optimum is context dependent.

In conclusion, our work underscores the critical role of an inherent spatiotemporal coupling between therapy-modulated nutrient landscapes, mutant growth and resistance escape in densely packed cellular populations. In essence, therapy failure risk is governed by whether therapy shifts the resource gradient long enough for bulk-born mutants to permanently escape confinement. Integrating the gained insights into our understanding of resistance evolution and therapy response will open new avenues for combating resistance-mediated therapy failure.

## Methods

### Strains

All experiments were performed using the genetically engineered strain yNA16 of the budding yeast *S. cerevisiae*. Strain yNA16 is derived from the ancestor strain yJK19^17^ and carries a β-estradiol-inducible Cre-EBD recombinase expressed from the *SCW11* promoter and integrated at the *HO* locus. The yeast yNA16 harbours a synthetic, irreversible recombination-based mutation cassette (*pGPD-loxP-yEmRFP-ubq-cyh2r-tCYC1-kanMX-loxP-yEGFP-ubq-Rrec*), enabling stochastic switching from a sensitive to a resistant state accompanied by a fluorescent colour change.

Thermosensitivity was introduced via an auxotrophic insertion at the *CDC26* locus (*pCDC26-RS-URA3-tURA3-RS-CDC26*). To enhance fluorescent signal in the switched state, a second activatable GFP cassette (*pGPD-RS-LEU2-tLEU2-RS-yEGFP-tADH*) was integrated. A switched derivative strain (yNA16s) was generated by inducing Cre activity with 3 μM β-estradiol in liquid culture, followed by selection at 35 °C. A resistant clone exhibiting stable green fluorescence was isolated and used for downstream experiments.

### Construction of yNA16

The yeast yNA16 was generated through sequential genetic modifications of yJK19^17^, through three genomic modifications. DNA fragments were amplified by PCR and assembled using NEBuilder HiFi DNA Assembly Master Mix (New England Biolabs, E2621). The underlying concepts were inspired by previous works^12,13,16,44^. Oligonucleotides used for plasmid and strain construction, including their sequences, are provided in Supplementary Data. Plasmid intermediates pNA7 and pNA8 were generated during construction of the primary switching cassette. Plasmid pNA7 was assembled from a pJR3214-derived backbone^45,46^, a *HygMX* fragment amplified from pAG32 (available from www.addgene.com, no. 35122) and a *ubiquitin* (*ubq*) fragment amplified from pJK19^17^. Plasmid pNA8 was subsequently assembled from a pNA7-derived backbone and a *ubq-cyh2r* fragment amplified from pJK19. Plasmid pNA8 was then used as the backbone for construction of pNA14. For pNA14, a pNA8-derived backbone was assembled with an R-recombinase-containing fragment amplified from pRS415-recR (a kind gift of J. Rine (UC Berkeley) containing the site-specific R-recombinase of *Zygosaccharomyces* *rouxii*^47^) and a *ubq* fragment amplified from pJK19 genomic DNA. To generate yNA14, the primary switching cassette was amplified from pNA14 and assembled with 369-basepair (bp) and 343-bp regions homologous to the sequences flanking the *URA3* integration site. The resulting approximately 7.2-kilobase integration fragment contained the cassette *pGPD-loxP-yEmRFP-ubq-cyh2r-tCYC1-kanMX-loxP-yEGFP-ubq-Rrec-tADH1* and was transformed into yJK19. Transformants were selected on the basis of G418 resistance conferred by *kanMX*, yielding the intermediate strain yNA14.

Thermosensitivity was introduced in a second modification to generate yNA15. A URA3-tURA3 fragment amplified from pJR3428^48^ was assembled with *pCDC26* and *CDC26* fragments amplified from yeast genomic DNA and bridging oligonucleotides introducing *RS*-sites. This led to the fragment with the architecture *pCDC26-RS-URA3-tURA3-RS-CDC26*. The fragment was transformed into yNA14 and transformants were selected on CSM lacking uracil.

Finally, an additional recombination-activated GFP cassette was integrated at the HIS3 locus. A pGPD fragment and a GFP-containing fragment were amplified from pJR3214 and an RS/LEU2-containing fragment was amplified from pRS415-recR. These fragments were joined together with two bridging oligonucleotides. The resulting approximately 3.71-kb cassette was amplified with overlaps for a second assembly and combined with upstream and downstream HIS3 homology regions amplified from yJK19 genomic DNA. The final approximately 4.48-kb integration fragment contained the construct *pGPD-RS-LEU2-tLEU2-RS-yEGFP-tADH* and was transformed into yNA15. Transformants were selected on CSM lacking leucine and functionally screened for recombinase-dependent thermoresistance and fluorescence switching.

Newly generated yeast strains and plasmids are available from the corresponding author upon reasonable request.

### Yeast transformation

Yeast transformations were performed using a lithium acetate/polyethylene glycol (LiAc/PEG)-based protocol. Yeast strains were inoculated into 5 ml of YPD and grown overnight at 30 °C. The following day, cultures were diluted into 50 ml of YPD to an initial optical density at 600 nm of 0.2 and grown at 30 °C with shaking for 3–4 h until reaching optical density at 600 nm of ~1.0. Cells were harvested by centrifugation (300*g*, 5 min), washed once with sterile distilled water and resuspended in 1 ml of distilled water. Aliquots of 100 μl were pelleted and resuspended in a transformation mix containing 50% (w/v) PEG3500, 1.0 M lithium acetate, boiled single-stranded carrier DNA and either PCR product DNA or plasmid DNA. Cells were incubated at 42 °C for 40 min, pelleted and gently resuspended in 250 μl of YPD. Transformants were plated on YPD agar or selective media and incubated at 30 °C. For antibiotic selection, colonies were replica-plated onto YPD plates containing the corresponding antibiotic after overnight recovery.

### Measuring growth rate

Growth rates of yNA16 and yNA16s at 30 °C and 35 °C were determined using plate reader assays. Thin liquid cultures were diluted 1:1,000 and 10 μl of each culture was added to 450 μl of YPD per well in a 48-well plate. Each strain was measured in 16 replicates per plate, with optical density at 600 nm recorded every 10 min. Plates were continuously shaken during incubation to promote exponential growth of all cells in suspension. Doubling times were estimated by fitting an exponential curve to the growth trajectories. The experiment at 30 °C was repeated twice to account for stochastic variation. To validate the temperature-dependent fitness difference under colony expansion conditions, we additionally measured expansion of linear inoculations with spontaneously arising resistant-clone sectors on solid agar at 30 °C, 31 °C, 32 °C and 35 °C. Colonies were first grown at 30 °C for 24 h and then transferred to their respective assay temperatures. After an additional 24 h, colonies were transferred to 4 °C to arrest further growth before imaging. Relative fitness differences were inferred from the opening angle between diverging clone boundaries in the linear expansions (Extended Data Fig. 1)^10^. In addition, we performed mixed-inoculation radial expansion assays using a 1:1,000 ratio of resistant yNA16s to susceptible yNA16 cells under the same temperature conditions to confirm neutrality in the absence of treatment.

### Arrayed colony assay

For the standard arrayed colony assay, *yNA16* populations were precultured on YPD + 2% agar supplemented with 200 μg ml^−1^ of G418 for 2 days, and then stored at 4 °C for up to 3 weeks. Monoclonal colonies from these plates were used to inoculate 3 ml of liquid YPD medium containing 200 μg ml^−1^ of G418 and grown for approximately 5 h at 30 °C. Cultures were vortexed for 5 min to disperse cell clusters and 30 μl were spread in a narrow band on one side of a monowell plate containing 70 ml of 1% agar YPD. The plates were dried under sterile conditions with the lid open for 45 min.

Single cells, preferentially with a small bud, were isolated using a Singer MSM400 dissection microscope and placed in a 5 × 4 grid, yielding a potential maximum of 20 colonies per plate. Plates were incubated at 30 °C and 85% relative humidity for 24 h to allow initial colony expansion, after which they were transferred to an on-stage incubation chamber for time-lapse imaging. Multichannel images were acquired every 30 min for up to 7 days. Temperature-based treatments and therapy holidays were implemented by adjusting the incubation chamber temperature manually or via a preprogrammed script. A type K thermocouple, placed on the agar surface at colony height, was used to monitor on-plate temperature. Colonies showing growth defects, petite mutations, contamination or physical obstruction due to agar irregularities were excluded from analysis. A summary of all experiments and the corresponding numbers of analysed colonies is provided in Supplementary Table 1.

### Adaptive therapy

AT was applied without a predetermined *τ*_on_/*τ*_off_ schedule. Instead, treatment phases were adjusted on the basis of colony growth. Treatment was initiated and maintained until colony expansion appeared fully arrested. Colonies were then released from treatment until growth resumed, followed by an additional recovery period to allow sensitive cell expansion to return to its pretreatment growth regime before treatment was re-applied. This adaptive procedure resulted in three applied treatment phases over the experiment runtime, as shown in Fig. 5a and Extended Data Fig. 8. The AT time-lapse terminated prematurely after 126 h. An additional endpoint image was acquired 16 h after the final time-lapse frame. Colony and resistant-clone areas were interpolated separately between the last time-lapse frame and this endpoint image.

### Imaging and analysis

Imaging was conducted using an AxioZoom V16 fluorescence microscope from Zeiss with an Objective Apo Z ×1.5/0.37 at a total magnification of 10.5 over the whole time span of an experiment. Final images were taken using the PlanApo Z ×0.5 objective at a total magnification of 6.3. As software for the acquisition we used Zen Blue 3.0. Images were then analysed using Python 3.11 scripts and Ilastik, a machine learning-based image segmentation tool, trained on time-spaced frames of randomly picked colonies^49^. All time-lapse data shown were segmented with the same trained model, assigning discrete labels for sensitive cells, resistant cells and background. Final endpoint images used for extrapolation were segmented with a separate model to ensure accuracy. For visualization purposes only, raw experimental images were processed in Fiji to adjust the display lookup table and colourmap. Radial kymographs were generated from segmented image stacks. For each colony, the centroid was determined and radial lines were sampled from the centroid to the border. Kymographs were computed over an angular window of ±2° with steps of 0.5° and combined by taking the pixel-wise minimum across angles. A 3 × 1 median filter (time × space) was applied for visualization only.

### Clonal tracking

Clone trajectories were filtered to restrict the analysis to comparably emerging and reliably tracked clones. Only clones whose first detection occurred between 12.5 h and 100 h were included, excluding late detections with insufficient temporal coverage. From this subset, clones were required to enter a defined spatial band relative to the colony front at least once during their lifetime, with distances constrained between 17 μm and 500 μm. This criterion restricted the analysis to clones originating within the actively growing region of the colony and excluded both central and edge-contact artefacts. Clones were further required to be tracked for a minimum of three consecutive frames.

Distances to the colony edge were converted to physical units using a scale factor of 8.648 μm per pixel. Clones were classified on the basis of their interaction with the colony front: a breach was defined as reaching a distance of ≤20 μm from the colony edge at least once. Following a breach, clones were classified as escaped if they did not subsequently retreat beyond 80 μm from the colony edge, whereas clones that never breached or retreated beyond this threshold were classified as confined. For visualization purposes only, trajectory traces were smoothed using a Savitzky–Golay filter; all classification decisions were based on unsmoothed data.

### Time-to-progression and out-of-frame interpolation

Time-to-progression was based on a fixed maximal colony area, corresponding to the area a perfectly round colony can occupy in the absence of imaging-related truncation. With the field of view used for time-resolved imaging, this area was determined to be 71 mm^2^. To avoid differential runtime bias, an experiment-wide censoring time was defined by the shortest non-progressing colony, excluding the NT condition, and all colonies were evaluated only up to this time. Colonies not reaching the threshold within this window were censored at the shared censoring time. Because TTP increases by definition when a larger area threshold is chosen, we also repeated the main schedule comparisons over a range of area thresholds to test robustness of the relative schedule ordering (Supplementary Fig. 9). Colony- and clone-area trajectories were corrected when growth extended beyond the field of view. Late trajectories were linearly interpolated between the last fully trusted time-lapse frame and a lower-magnification endpoint image (Supplementary Fig. 10a). Under treatment, resistant clones could expand asymmetrically and partially leave the imaging frame, leading to an underestimation of clonal area. From this frame onward, the total clonal area was linearly extrapolated to the final clonal area measured from the corresponding lower-magnification endpoint image. Corrected colony area was then calculated by replacing the measured clonal area with the extrapolated clonal area. For the NT condition, no extrapolation was applied, as resistant sectors did not reach the field of view boundary and thus did not contribute to growth outside the imaged area. Uncertainty introduced by interpolation to the final endpoint was estimated from fully observed colonies of the intermittent 6.5 h/18 h schedule. Linear interpolation was initiated at alternative late time points and extended to the independently segmented final image, after which the interpolated colony and clone areas at 125 h were compared with the measured time-lapse ground truth. Absolute percentage errors were calculated across interpolation start points, excluding colonies that reached the image border before or at the ground-truth frame. The largest median absolute percentage error (0.78%) was used as an empirical relative uncertainty estimate for colony area in subsequent robustness analyses (Supplementary Fig. 10b,c). For time-resolved visualization of population-level trajectories and resistant fraction, the median and interquartile range (IQR) across colonies were computed per time point. To reduce frame-to-frame variability, curves were smoothed using a centred rolling median filter (window size 4.5 h). Smoothing was applied for display purposes only and did not affect statistical analyses or classification criteria. Statistical comparisons of TTP and resistant fraction between treatment schedules were performed using one-sided Mann–Whitney *U*-tests with Holm correction for several comparisons across the tested schedule pairs. Effect sizes for TTP were reported as median differences between schedules. Confidence intervals for these median time-to-progression differences were estimated by nonparametric bootstrapping, resampling colonies within each schedule with replacement.

### Simulation

We built a custom 2D lattice simulation consisting of sensitive and resistant cells interacting via a nutrient layer on a 200 × 200 grid, where the cells have periodic boundary conditions and the nutrients have Dirichlet boundary conditions, leading to a constant influx of nutrients from the boundary. The simulation starts with a single sensitive cell in the centre pixel, from which we pregrow the colony until it reaches a size similar to that observed in the first experimental image.

Space is divided into a 2D grid of demes, each containing a mixture of sensitive cells *S* and resistant cells *R* that proliferate at rate *α*, mutate stochastically (${{\mathcal{M}}}_{\mu }$) and disperse with an effective diffusion coefficient *D*. To emulate the growth-dependent mutation and dispersal of our experiments, these processes are only applied to the actively growing per-deme populations of sensitive and resistant cells *S*_d_ = *αξNS* and *R*_d_ = *αNR*, respectively:

1 $$\frac{\partial S}{\partial t}={S}_{{\rm{d}}}+D\Delta {S}_{{\rm{d}}}-{{\mathcal{M}}}_{\mu }({S}_{{\rm{d}}})\,,$$

2 $$\frac{\partial R}{\partial t}={R}_{{\rm{d}}}+D\Delta {R}_{{\rm{d}}}+{{\mathcal{M}}}_{\mu }({S}_{{\rm{d}}})\,.$$

Additionally, dispersal only occurs after a defined population threshold is reached in a deme. This threshold is cell-type-specific. Experimentally observed delays in treatment effects on growth rate are implemented via the therapy-induced growth-rate modulation *ξ* ∈ [0, 1], exhibiting an on-delay *δ*_on_, overshoot phase *δ*_over_, lag-delay *δ*_lag_ and off-delay *δ*_off_. Note that the overshoot phase is part of the lag phase. Stochastic mutations are randomly sampled from a Poisson distribution ${{\mathcal{M}}}_{\mu }$ defined by the expectation value *μS*_d_, with *μ* being a mutation prefactor and then divided by a scaling factor to convert the sampled number to the scale used in the simulation.

Importantly, competition is solely mediated via nutrient limitation without introducing a local carrying capacity. Nutrients are modelled as a freely diffusing consumable resource with the diffusion coefficient *D*_N_:

3 $$\frac{\partial N}{\partial t}={D}_{{\rm{N}}}\Delta N-\beta ({S}_{{\rm{d}}}+{R}_{{\rm{d}}})\,,$$

with *β* being the consumption rate of nutrients. If the defined TTP size is not reached, the endpoint of the simulation is used as the TTP value. If the simulation time is increased beyond TTP, the boundary seen in Fig. 4 will shift to smaller *τ*_on_ values due to finite time effects until it reaches a final value.

All median trajectory plots of simulation data are plotted with a rolling average of window size 2.5 h. The area growth rate and front growth rates additionally have a rolling average of the same window size applied during calculation.

#### Cytotoxic treatment

Cytotoxic treatment is implemented by removing the original modification of the sensitive cell growth rate and instead adding it as a modification of a newly introduced death term with a chosen maximum death rate.

#### Spatial treatment

Spatial treatment is implemented by multiplying the current treatment value with an array that is zero outside the colony, equals one at the front and then decays exponentially from there inward. The decay factor can be varied.

#### Fitting

The treatment strength *E* increases linearly with time from zero to one during treatment, reaching full effect after *δ*_on_ = 247 simulation steps (12.35 h). If the treatment ends before full effect is reached, *E* continues to increase for another *δ*_over_ = 30 simulation steps (1.5 h). After treatment ends *E* has a *δ*_lag_ = 220 simulation steps (11 h) lag phase including the overshoot time before it decreases linearly back to zero in *δ*_off_ = 244 simulation steps (12.2 h). If treatment starts again before zero is reached, an immediate switch occurs to the treatment-on dynamics. These delays are determined directly from the experiment by analysing the radial growth over time (Supplementary Fig. 1a) for a 7 h/18 h intermittent schedule and 14-h pulse experiment. Example effective growth rates (*ξ* = 1 − *E*) for different schedules are shown in Supplementary Fig. 11. The growth rate *α* is calculated from the doubling time *τ*_2_ of the sensitive cell population determined via a plate reader experiment using:

4 $$\alpha =\frac{\log (2)}{{\tau }_{2}}.$$

The cell diffusion coefficient *D*, the nutrient diffusion coefficient *D*_*N*_ and the consumption rate *β* are fitted together using the Nelder–Mead method. The value we minimize is the sum of two normalized mean squared errors (NMSEs), each targeting a different, but not independent, system dynamic. The first part is calculated by comparing the radial expansion of 13 experimental replicates of the NT condition to the simulation (Supplementary Fig. 1b). We calculate the NMSE for each replicate and then average over the replicates, yielding us the first part of the sum. The second part is calculated from the time it takes for a clone to start growing again after treatment starts over the distance to the front (experimental data from Fig. 2). We choose four distances (2 pixels, 4 pixels, 6 pixels, 8 pixels) and initialize the simulation with exactly one mutated cell at the corresponding distance along the cardinal directions, simulate treatment and compare the time it takes for the mutated population to reach the next pixel closer to the front to the time measured from the experiment (Supplementary Fig. 1c). For this, we again calculate the NMSE. Given the result of this fit, we next fit the mutation prefactor by minimizing the squared difference between the number of mutation events in the simulation and the experiment in the case of CT using the Nelder–Mead method. The mutation scaling is obtained by dividing the size of one simulation pixel in micrometres by the size of a single cell, which is taken from the average mask size of a segmented colony image in single-cell resolution (Supplementary Fig. 1d). The segmentation is performed using cellpose^50^. The final parameter set is available in the code repository and in Supplementary Table 2. Comparison runs between the simulation and the experiment are shown in Supplementary Fig. 12.

#### Bootstrapping

The uncertainties of the Nelder–Mead-fitted parameters are estimated by 1,000 bootstrap refits using experimental data resampled with replacement. Uncertainties of the remaining derived parameters are obtained by sampling or propagating the corresponding experimental uncertainties, or from the uncertainty estimates of the underlying fits (Supplementary Table 2).

#### Leave-one-out cross-validation

The leave-one-out cross-validation is implemented as a hierarchical paired leave-one-out validation to separate fitting from validation data: in each fold, one NT trajectory and one CT trajectory are held out; all fitting steps are then rerun using only the remaining training data, and the resulting fold-specific model is evaluated on the held-out data (Supplementary Table 3).

### Nutrient threshold

The nutrient thresholds *N*_low_ and *N*_high_, which define the positions of *λ*_low_ and *λ*_high_, are defined as the values of a sigmoidal function at the position where a linear function with the slope of the sigmoid function at 0 intersects 0 and 1 (Supplementary Fig. 13). With this, ${N}_{{\rm{low}}}=\frac{1}{1+{e}^{2}}$ and ${N}_{{\rm{high}}}=\frac{1}{1+{e}^{-2}}$.

### Reporting summary

Further information on research design is available in the Nature Portfolio Reporting Summary linked to this article.

## Supplementary information

Supplementary Information Supplementary Text, Tables 1–3 and Figs. 1–13.

Reporting Summary

Peer Review File

Supplementary Video 1 Video of the example colony shown in Fig. 1g using *τ*_on_ = 11.5 h and *τ*_off_ = 21 h. The grey outline indicates the current treatment state.

Supplementary Video 2 Video of the example colony shown in Fig. 2c. The applied treatment is continuous dose. The grey outline indicates the current treatment state.

Supplementary Video 3 Video of the example colony shown in Fig. 2f. The applied treatment is a 14-h pulse. The grey outline indicates the current treatment state.

Supplementary Video 4 Video of the simulated example colony shown in Fig. 3e. On the left the colony is shown, on the right the corresponding nutrient landscape. The applied treatment is continuous dose. The grey outline indicates the current treatment state.

Supplementary Video 5 Video of the simulated example colony shown in Fig. 3h. On the left the colony is shown, on the right the corresponding nutrient landscape. The applied treatment is a 14-h pulse. The grey outline indicates the current treatment state.

Supplementary Video 6 Video of the simulated example colony shown in Fig. 4h using *τ*_on_ = 4 h and *τ*_off_ = 18 h. On the left the colony is shown, on the right the corresponding nutrient landscape. The grey outline indicates the current treatment state.

Supplementary Video 7 Video of the simulated example colony shown in Fig. 4h using *τ*_on_ = 6.5 h and *τ*_off_ = 18 h. On the left the colony is shown, on the right the corresponding nutrient landscape. The grey outline indicates the current treatment state.

Supplementary Video 8 Video of the simulated example colony shown in Fig. 4h using *τ*_on_ = 9 h and *τ*_off_ = 18 h. On the left the colony is shown, on the right the corresponding nutrient landscape. The grey outline indicates the current treatment state.

Supplementary Video 9 Video of the example NT colony used in Fig. 5g with no treatment applied. The grey outline indicates the current treatment state.

Supplementary Video 10 Video of the example colony shown in Fig. 5g,h using *τ*_on_ = 4 h and *τ*_off_ = 18 h. The grey outline indicates the current treatment state.

Supplementary Video 11 Video of the example colony shown in Fig 5g,h using *τ*_on_ = 6.5 h and *τ*_off_ = 18 h. The grey outline indicates the current treatment state.

Supplementary Video 12 Video of the example colony shown in Fig 5g,h using *τ*_on_ = 9 h and *τ*_off_ = 18 h. The grey outline indicates the current treatment state.

Supplementary Video 13 Video of the example continuous dose colony shown in Fig 5g,h. The grey outline indicates the current treatment state.

Supplementary Video 14 Video of the example continuous dose colony shown in Fig 5g,h. The grey outline indicates the current treatment state.

Supplementary Data Oligonucleotides used for plasmid and strain construction of yNA16.

## Data Availability

The source and processed data underlying the figures are available via figshare at https://doi.org/10.6084/m9.figshare.31356370.v3 (ref. ^51^). The analysis code used to generate the figures is available via GitHub at https://github.com/KayserLab/Appold_Citak_2026.git. Raw imaging datasets are too large for public deposition but are available from the corresponding author upon reasonable request.

## Code availability

Custom code used for the simulations is available via GitHub at https://github.com/KayserLab/Appold_Citak_2026.git. The repository contains all scripts necessary to reproduce the simulation results and figures presented in this study.
