# Supplementary material for: A microbial therapy-mimicry assay shows how spatial resource dynamics control resistance escape
Source: Nat Ecol Evol. 2026 Sep 28;10(11):2160–73. doi: 10.1038/s41559-026-03178-z (PMC13736643; doi:10.1038/s41559-026-03178-z)
Supplement: Supplementary file 1 [file 41559_2026_3178_MOESM1_ESM.pdf]

nature ecology & evolution

Supplementary information

https://doi.org/10.1038/s41559-026-03178-z

A microbial therapy-mimicry assay shows how spatial resource dynamics control resistance escape

Supplementary Information

Transition analysis Asking whether this boundary reflects a true dynamic bifurcation rather than only a steep but continuous crossover, we identify two superimposed mechanisms: the cycle-to-cycle ratchet described above, and an underlying schedule-independent threshold in peripheral growth. Consistent with the first contribution, the switch from approximately Markovian to non-Markovian treatment kinetics coincides with a jump in long-term cycle-averaged growth near the boundary (Extended Data Fig. 2). In addition, we conducted constant-treatment simulations across different treatment strengths to decouple γ_{front} from treatment history. These simulations reveal an additional sharp crossover at a threshold treatment strength, with $\gamma_{\text{front}}/\gamma^*_{\text{front}}$ as the underlying governing parameter, where γ^*_{front} is a system dependent threshold that quantifies whether sensitive-front expansion remains sufficient to offset resistance-mediated advantages and maintain confinement (Extended Data Fig. 3). Near this threshold, replicate simulations separate into sensitive-dominated and resistance-dominated endpoint states, producing a bimodal distribution of resistant fractions (Extended Data Fig. 4). While the bimodality, accompanied by a pronounced anomaly in Binder cumulant, changes in skewness, and a peak in standard deviation near the same treatment threshold, is consistent with the notion of a true dynamic bifurcation in the simulations it is not yet enough to proof that the phase-transition-like boundary is a true dynamic bifurcation (Extended Data Fig. 5).

Name or Schedule ($\tau_{\text{on}}/\tau_{\text{off}}$)	No. Colonies
No treatment	13
Continuous treatment	9
Pulse (14 h)	12
Pulse Test (2 h)	10
Pulse Test (4 h)	13
Pulse Test (6 h)	10
Pulse Test (8 h)	15
Pulse Test (10 h)	11
Pulse Test (12 h)	13
Pulse Test (14 h)	13
Pulse Test (16 h)	12
Pulse Test (18 h)	10
Pulse Test (20 h)	10
4/18	10
6.5/20.5	8
6.5/18	25
6.5/15.5	10
9/20.5	15
9/18	10
Adaptive	8

Supplementary Table 1: **Experimental setups and corresponding number of colonies**

Parameter	Full-data fit	mean	SD	95% CI	Method	Unit
Sensitive dispersion (D)	0.42	0.42	0.04	0.34–0.50	BR	px^2
Resistant dispersion (D)	0.42	0.42	0.04	0.34–0.50	tied BR	px^2
Nutrient consumption (β)	2.6	3.0	0.6	2.2–4.3	BR	unitless
Nutrient diffusion (D_{N})	0.040	0.032	0.008	0.017–0.041	BR	px^2 $step^{-1}$
Pregrowth duration	304	305	15	275–328	BR	step
Mutation prefactor (μ)	0.59	0.74	0.16	0.53–1.11	BR	mut./growth inc.
Mutation scaling factor	680	680	14	654–708	MU	unitless
Sensitive proliferation rate (α)	0.0245	0.0245	0.0005	0.0235–0.0255	MU	$step^{-1}$
Resistant proliferation rate (α)	0.0245	0.0245	0.0005	0.0235–0.0255	tied MU	$step^{-1}$
Treatment on-delay (δ_{on})	247	248	8	232–265	FU	step
Treatment off-delay (δ_{off})	244	247	23	206–301	FU	step
Treatment lag-delay (δ_{lag})	220	218	6	210–230	FU	step
Treatment overshoot phase (δ_{over})	30	29	3	20–30	FU	step

Supplementary Table 2: Simulation parameters and their uncertainty estimates obtained using different methods as indicated: BR, uncertainty estimate from 1000 bootstrap refits; MU, resampled 1000 times from underlying measurement uncertainty; FU, resampled 1000 times from underlying fitting uncertainty; tied, constrained to the corresponding sensitive-cell parameter.

Parameter	Full	mean	SD	min–max range	Unit
Regrowth onset time (t_0)	9.2	9.1	2.3	6.5–13.4	h
Regrowth slope (m)	15.9	15.9	0.6	15.3–17.2	µm h^{-1}
Sensitive-cell dispersion (D)	0.422	0.414	0.030	0.268–0.447	px^2
Nutrient consumption rate (β)	2.58	2.61	0.25	1.36–3.70	unitless
Nutrient diffusion (D_{N})	0.040	0.037	0.007	0.017–0.040	px^2 $step^{-1}$
Pregrowth duration	304	303	7	280–312	step
Mutation prefactor (μ)	0.59	0.62	0.08	0.42–1.04	mut./growth inc.

Supplementary Table 3: Leave one out cross validation (LOOCV) parameter stability for parameters refit during the LOOCV.

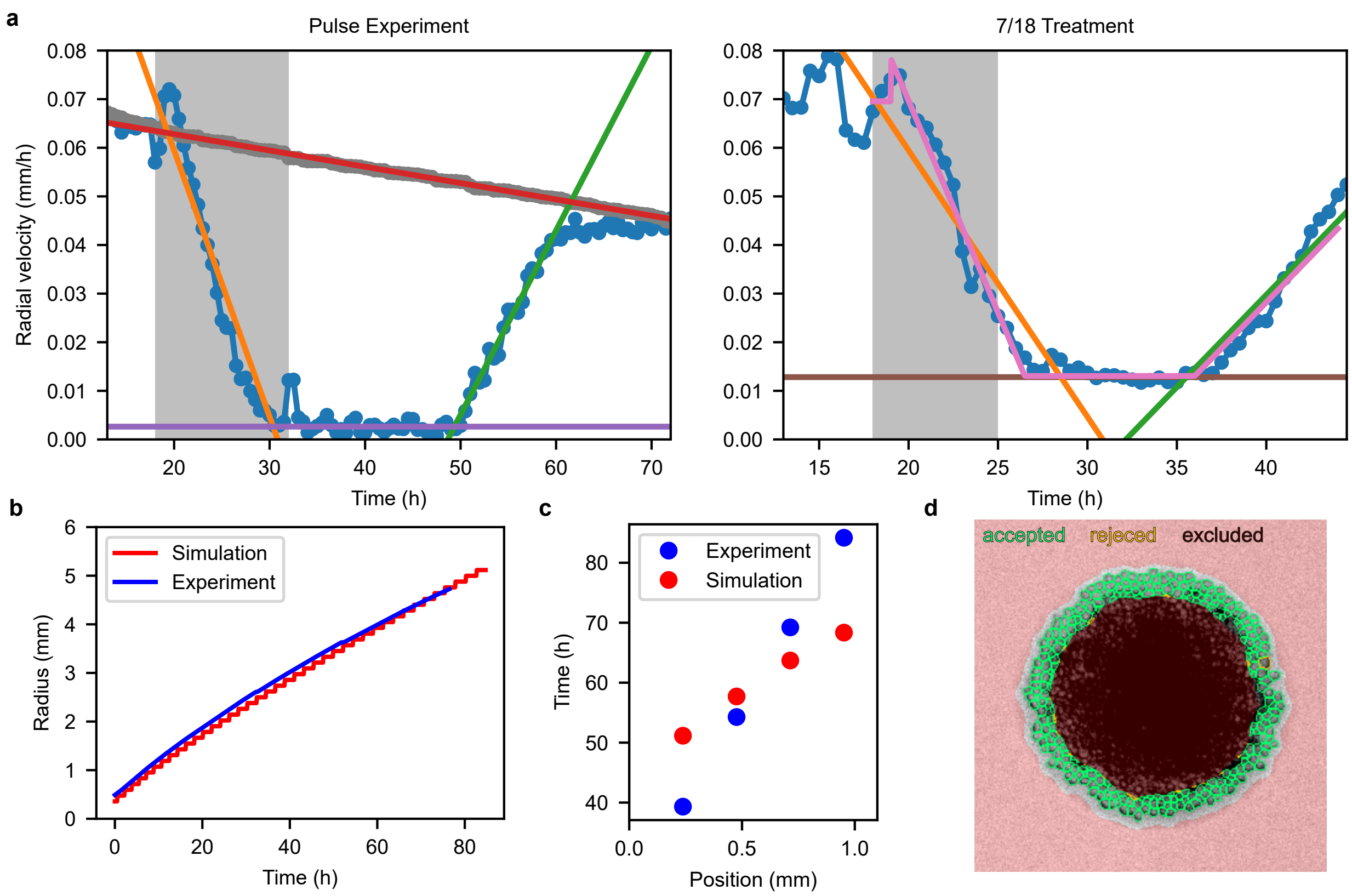

Supplementary Figure 1: **a** The blue line is the mean of a rolling median with window size = 9: In the left panel it is the pulse experiment ($n = 12$), and in the right panel it is a 7/18 schedule escperiment ($n = 17$). Treatment delays fit: The orange line in the left panel from treatment start until it intersects with the purple line gives the duration of the δ_{on} delay. The x-axis difference between the intersection of the green line with the purple line and the red line gives the δ_{off} delay, the red line being the maximum radial velocity at that time point taken from a no treatment experiment. The right panel gives the δ_{over} and δ_{lag} delays. The lag delay is the x-axis difference from the treatment end point until the pink line starts to increase again, while the overshoot delay is the short duration after treatment ends it still takes for the pink line to reach its minimum. Gray shading indicates treatment. The blue line is **b** Radial expansion fit in the no treatment scenario. **c** Time it takes a resistant clone to start growing again after treatment starts at 18 h depending on the distance from the colony front. For this continuous treatment was used. **d** High-magnification image used for calculating the average size of a cell, which is used to get the scaling factor from cells to simulation pixel value. The green cells are accepted, the orange ones are rejected and the red areas are excluded from the analysis. The segmentation was performed with cellpose [50].

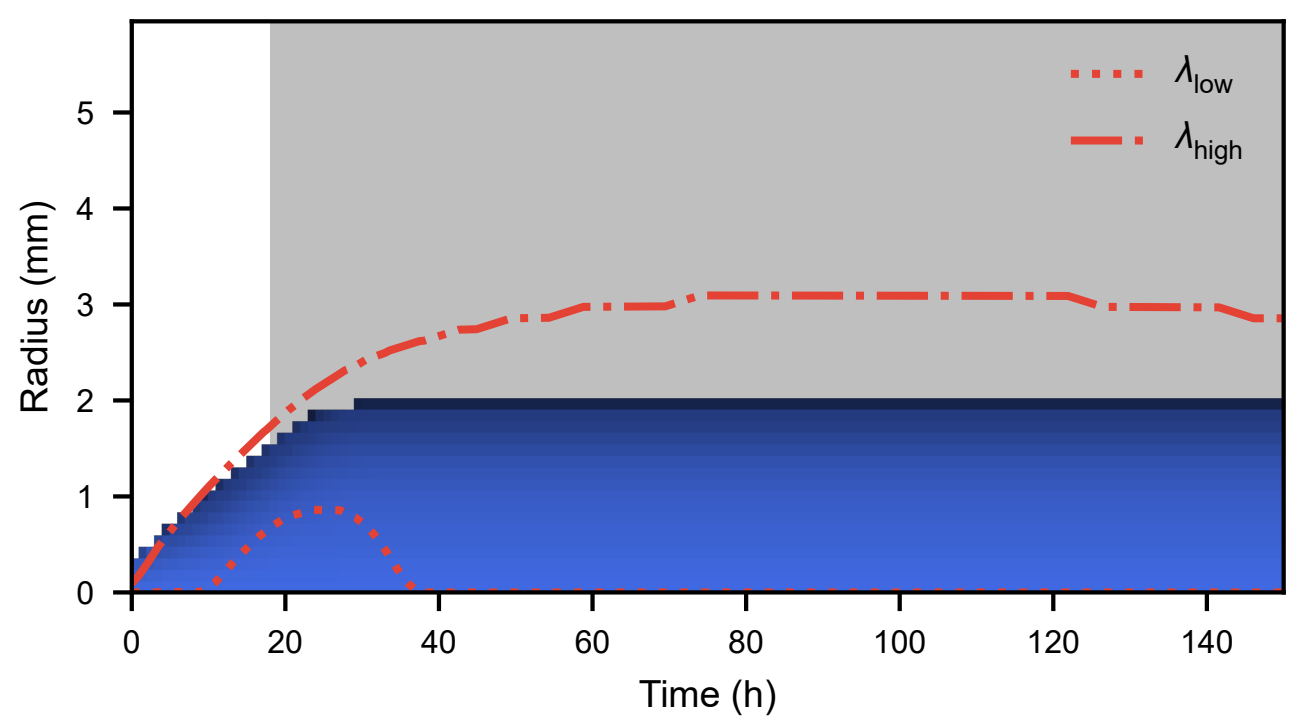

Supplementary Figure 2: Example of how the nutrient dynamics indicated by λ_{low}, λ_{high} evolve under continuous treatment if no mutations occur.

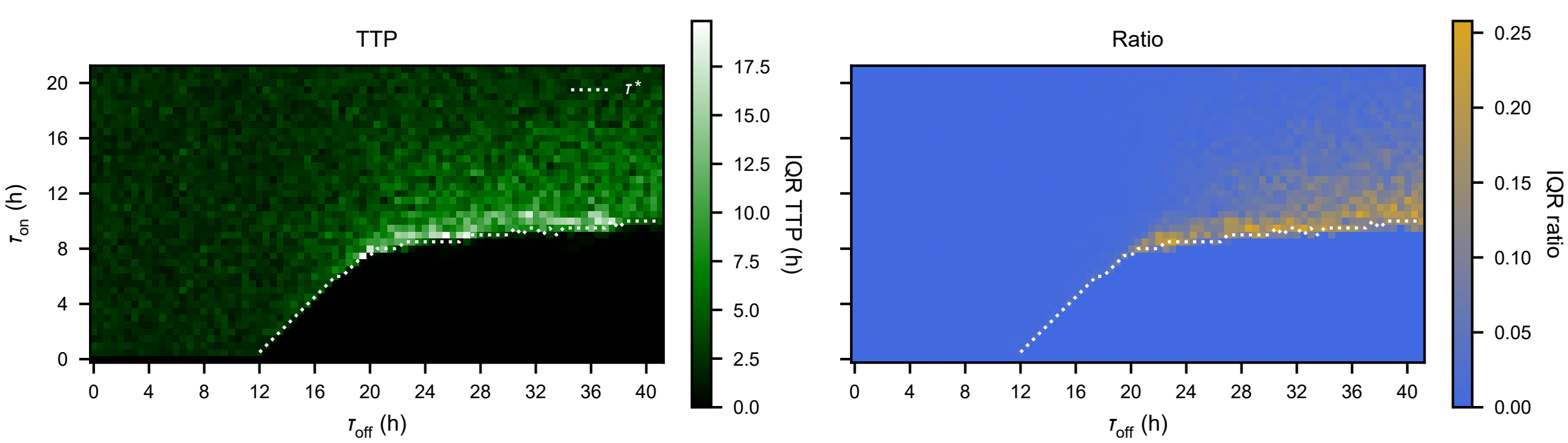

Supplementary Figure 3: IQR ($n = 20$) for the full parameter sweep in Fig. 4a, b.

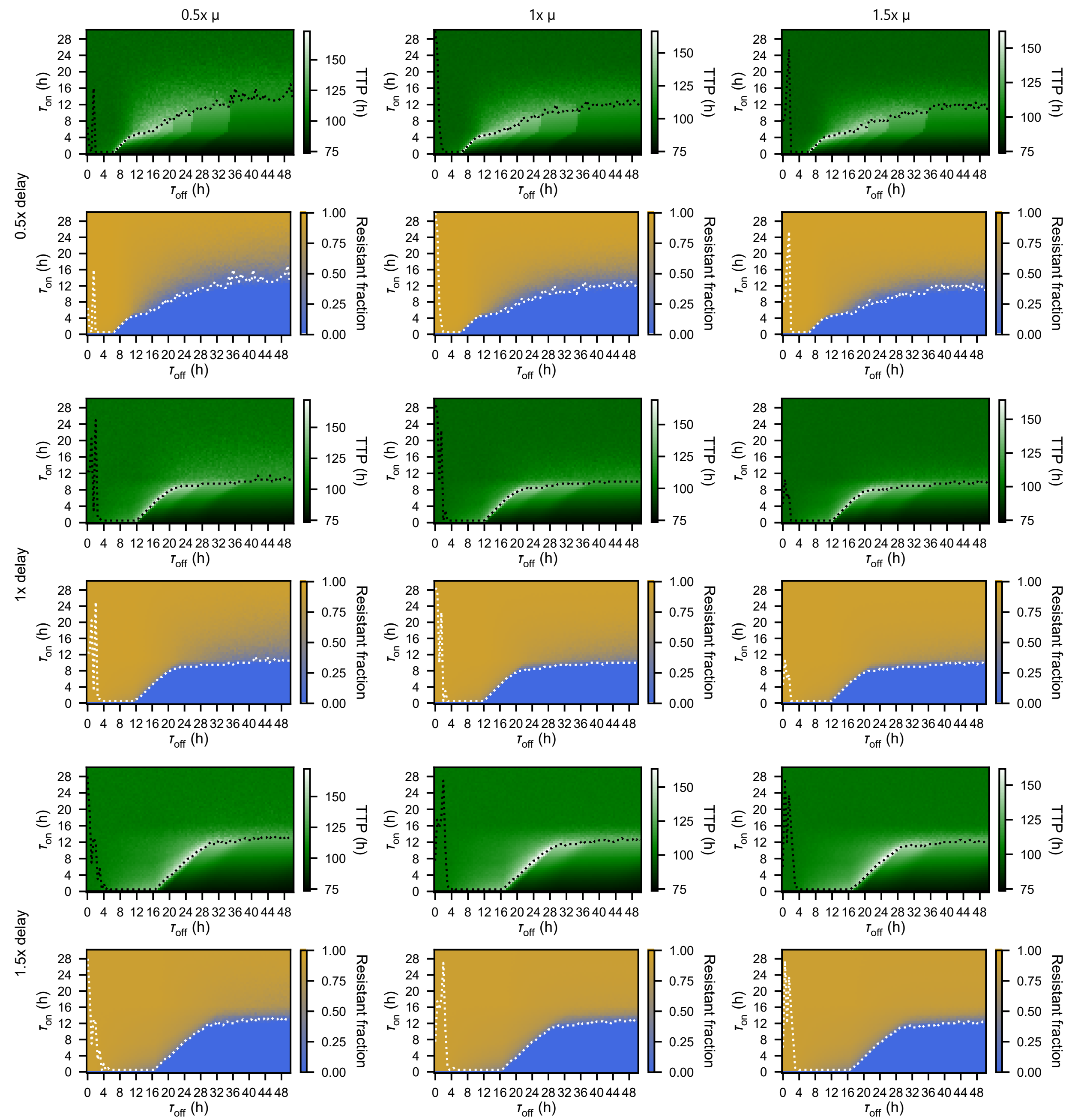

Supplementary Figure 4: Full parameter sweep plots for the sweep shown in Fig. 4a, b as well as sweeps with 0.5x and 1.5x the mutation prefactor and total delay duration. Each pixel is the median of 20 replicates.

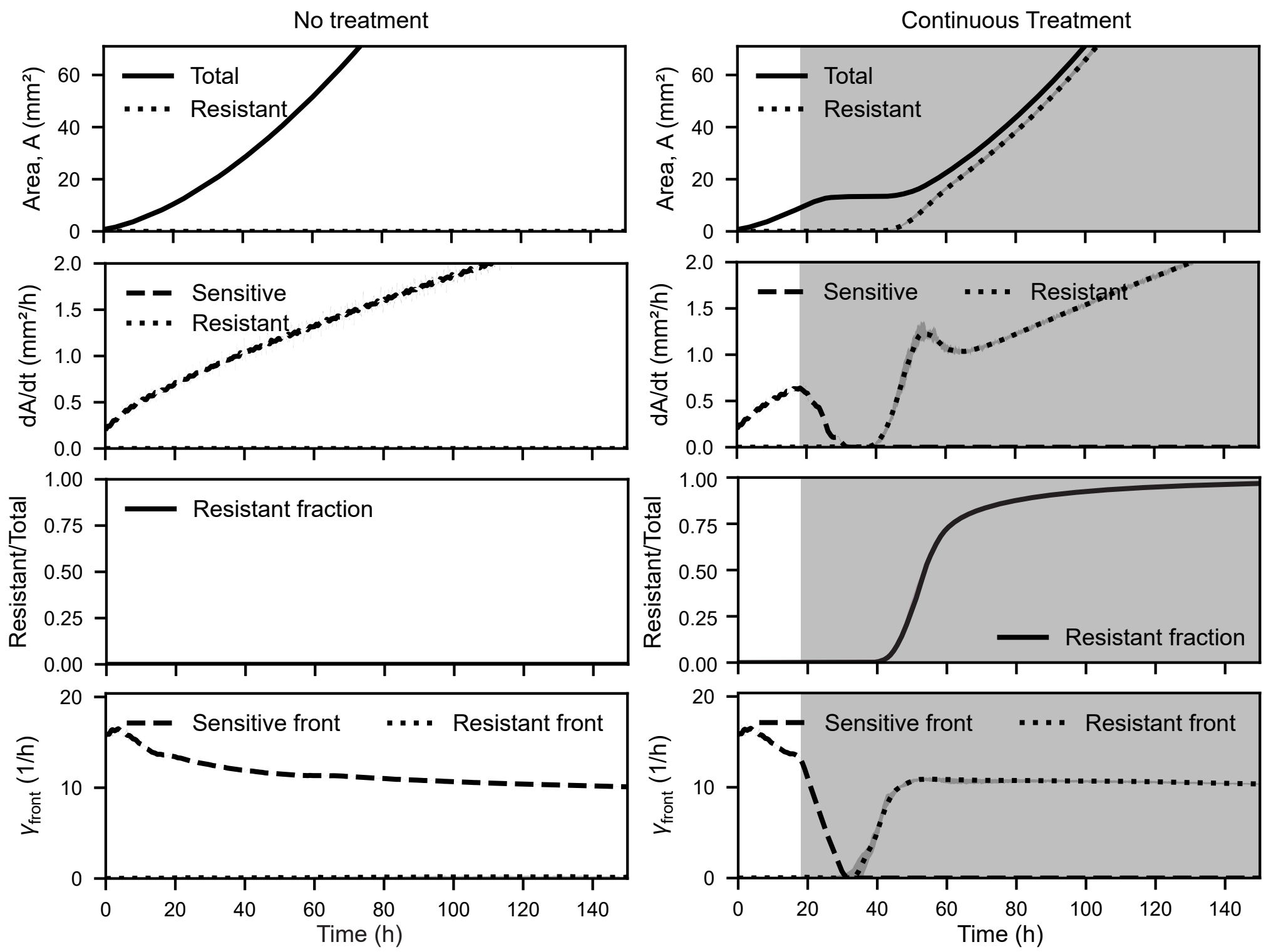

Supplementary Figure 5: Total and resistant area, sensitive and resistant area growth rates, resistant fraction, and front cell growth speeds for the no treatment and continuous treatment scenarios. The lines are the median over $n = 20$ colonies, with the shaded area being the IQR. Gray shading indicates treatment.

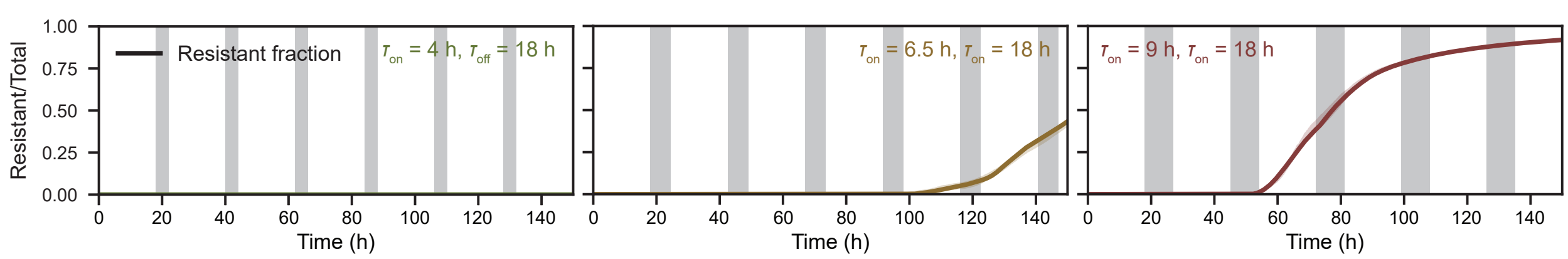

Supplementary Figure 6: Median resistant fractions ($n = 20$) with their respective IQRs for the treatments shown in Fig. 4. Gray shading indicates treatment.

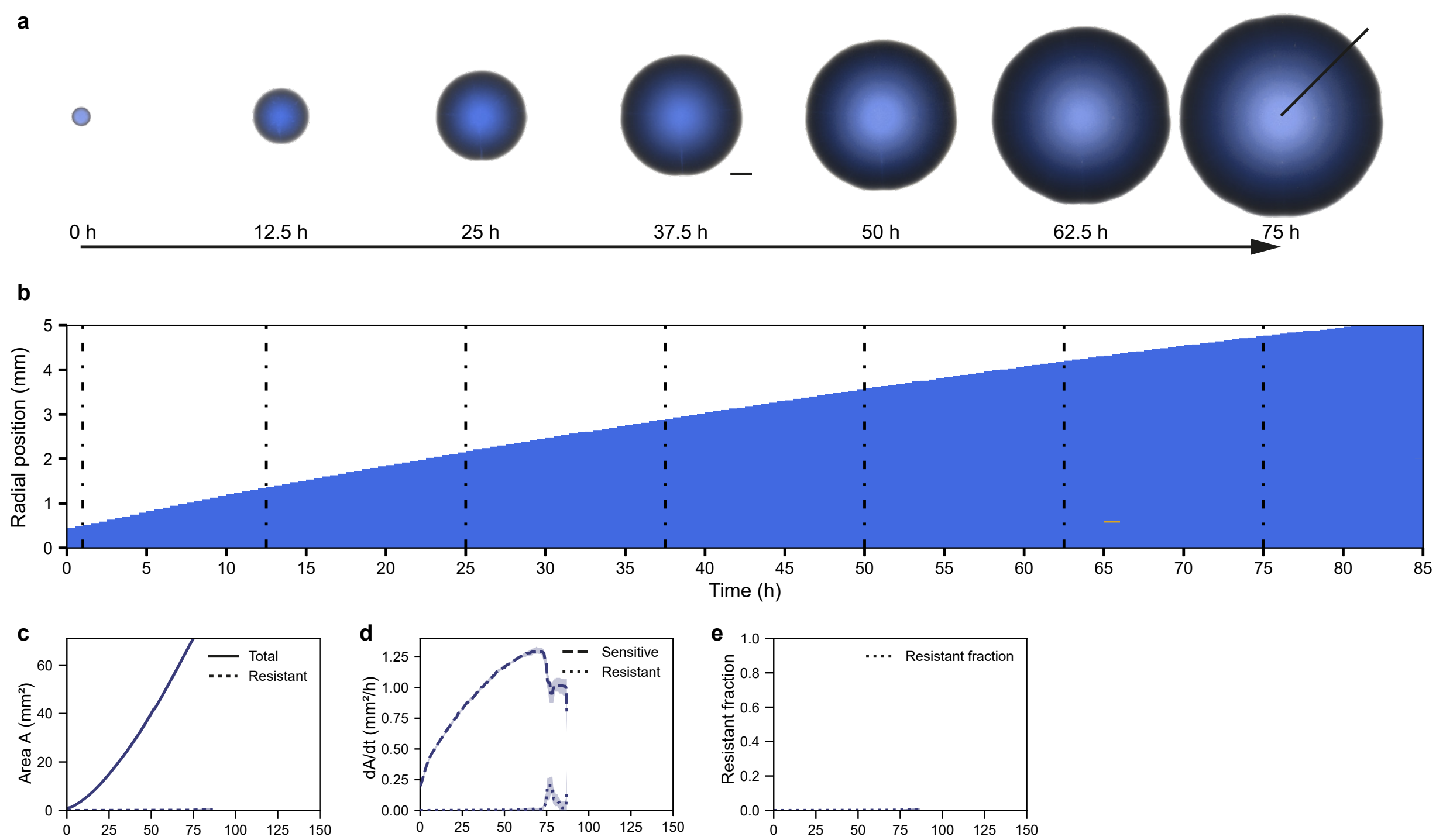

Supplementary Figure 7: **a** Representative time-lapse images of a no-treatment experiment. Imaging starts ($t = 0$ h) 24 h after seeding. Time points are at 1, 12.5, 25, 37.5, 50 and 62.5 h after imaging start. Scale bar indicates 1 mm. **b** Radial kymographs extracted from the colony depicted in **a** (black radial at 45 °). Dash-dotted lines correspond to the time points shown in (**a**). **c** Median total and resistant colony area for the no treatment (NT) experiment. **d** Median growth dynamics of sensitive and resistant populations. **e** Median temporal evolution of the resistant fraction (median, IQR). **c-e** Sample size is $n = 13$ colonies.

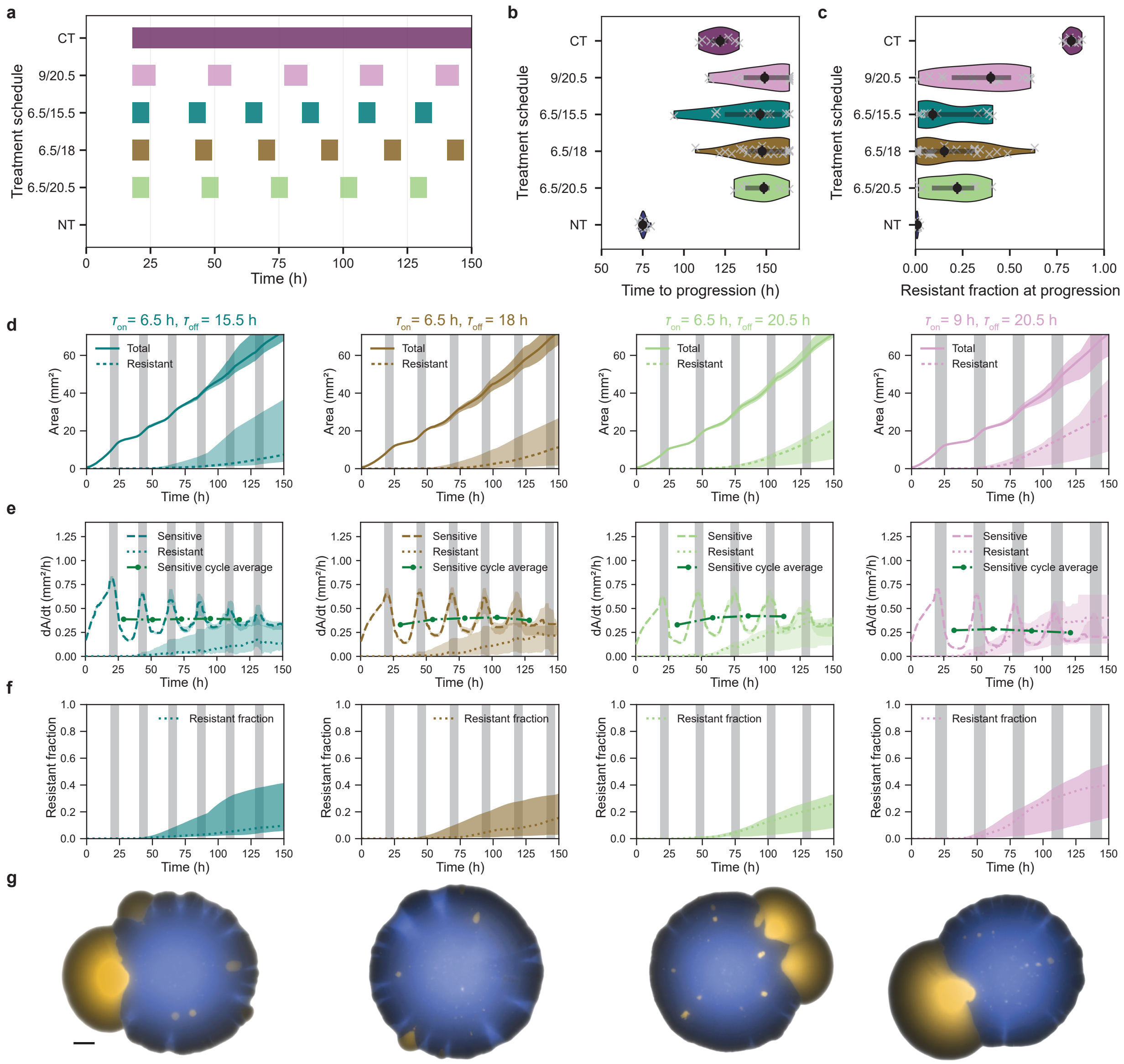

Supplementary Figure 8: **a** Additional experimentally tested schedules and corresponding τ_{on} intervals. **b** Violin plots of time to progression (TTP), defined as the first time point at which colony area reached ≥ 71 mm^2, for indicated treatment schedules. Inner bars show the interquartile range; dots indicate the median. **c** Resistant cell fraction at TTP (median, IQR). **d** Median total and resistant colony areas for the different intermittent schedules ($\tau_{\text{on}}/\tau_{\text{off}}$: 6.5 h/20.5 h, 6.5 h/15.5 h, 6.5 h/18 h and 9 h/20.5 h). **e** Median growth dynamics of sensitive and resistant populations, including cycle-averaged sensitive growth. Shaded areas indicate IQR. **f** Median temporal evolution of the resistant fraction (median, IQR). **g** Images at 120 h corresponding to the median TTP colonies of schedules shown in d-f. Scale bar, 1 mm. Grey shading denotes treatment periods. **b-f** Sample sizes were $n = 9$ colonies for CT, $n = 8$ for 6.5 /20.5 h, $n = 10$ for 6.5 /15.5 h, $n = 25$ for 6.5 h/18 h, $n = 15$ for 9 h/20.5 h, and $n = 13$ for NT

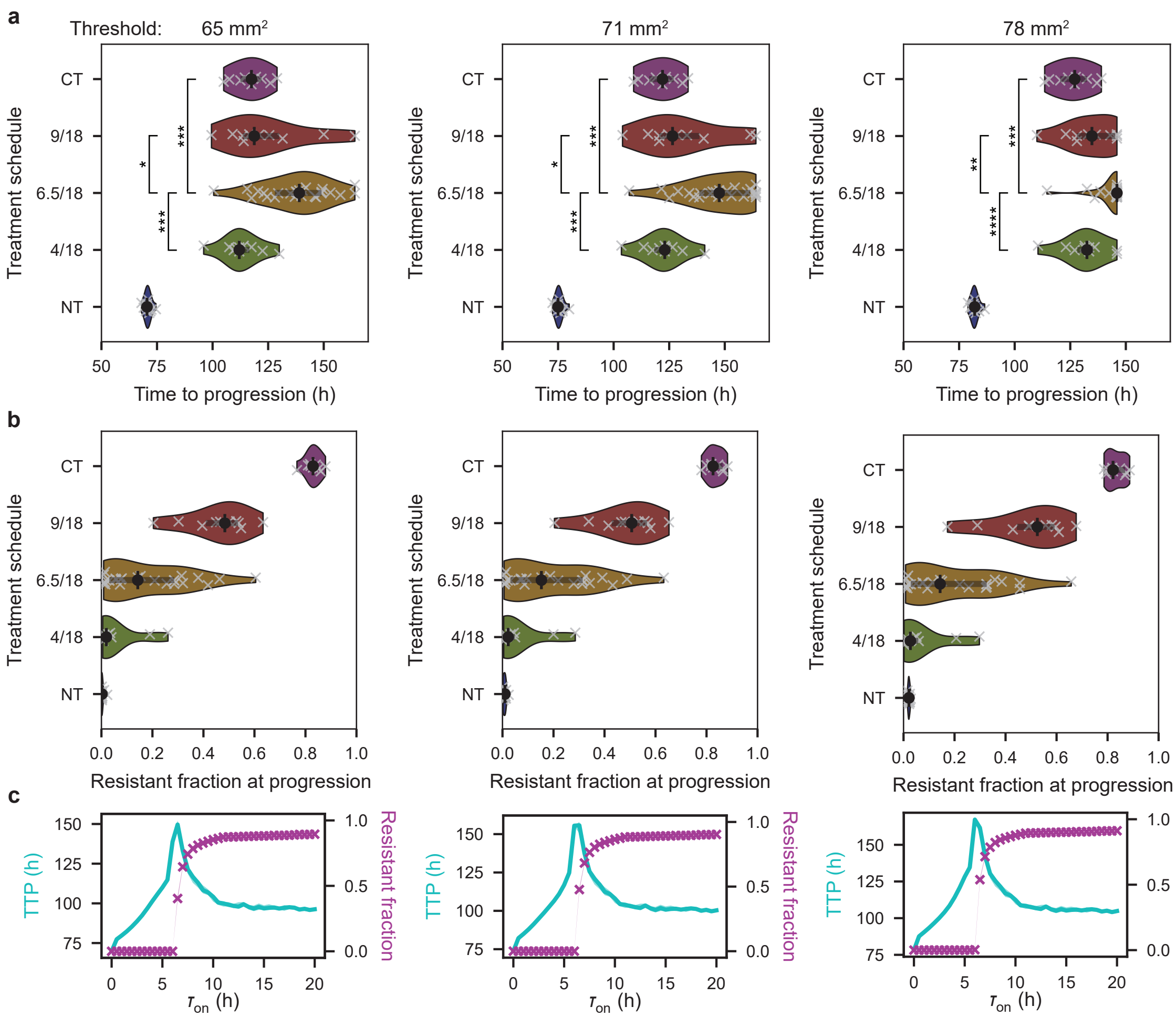

Supplementary Figure 9: **a** Comparison of time to progression (TTP), defined as the first time point at which colony area reached ≥ 65 mm^2, ≥ 71 mm^2 or ≥ 78 mm^2, for indicated treatment schedules. Inner bars show the interquartile range; dots indicate the median. **b** Resistant cell fraction at TTP for different thresholds (median, IQR). **c** Predicted median TTP optimum and transition point from the sensitive dominated to resistant dominated regime shown in the median resistant fraction in the simulation. The shaded area indicates IQR ($n = 20$). **a-b** Sample sizes were $n = 25$ colonies for 6.5 h/18 h, $n = 9$ for CT, $n = 10$ for 9 h/18 h, $n = 10$ for 4 h/18 h, and $n = 13$ for NT.

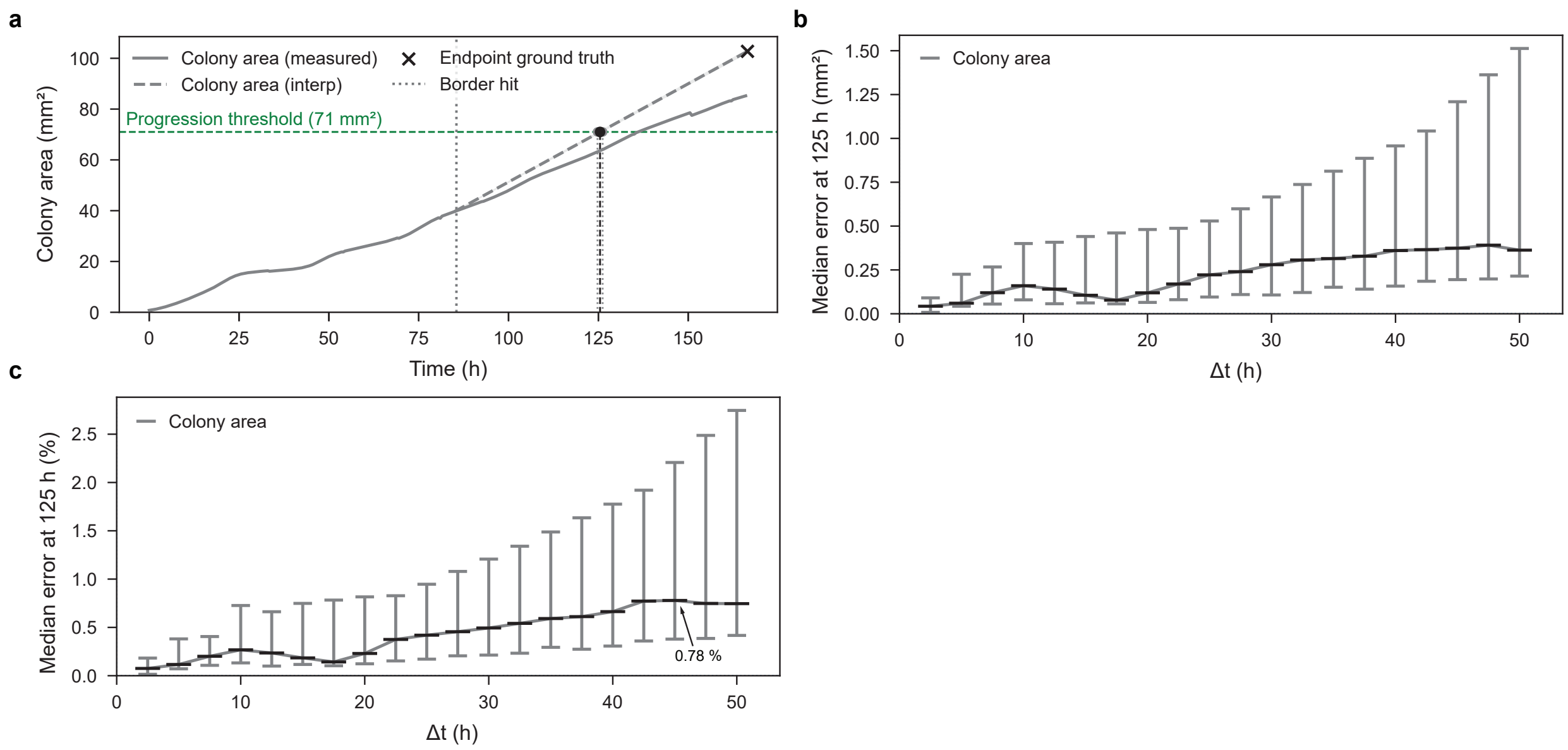

Supplementary Figure 10: **a** Representative colony-area trajectory illustrating the uncertainty estimate for time to progression (TTP). Measured colony area is shown as a solid gray line. After the colony first reaches the border of the field of view, colony area was extrapolated to the endpoint ground truth, indicated by a black cross. The progression threshold was set to 71 mm^2. A relative uncertainty of 0.78% was applied to the extrapolated colony area (shaded area) and propagated to the threshold crossing. For the example shown, this resulted in TTP estimates of 125.473 h for the central extrapolation, 126.195 h for the lower area bound, and 124.763 h for the upper area bound. **b** Absolute interpolation error of colony area at 125 h for different interpolation start points. Colony trajectories from the 6.5 h/18 h schedule that had not reached the image border at 125 h were used for this validation ($n = 16$). For each start point, the trajectory was interpolated to 125 h and compared with the measured ground truth at 125 h. The black line indicates the median error and grey whiskers show the interquartile range. **c** Relative interpolation error of colony area at 125 h for the same validation shown in **b**. The maximum median relative error was 0.78%, observed for $\Delta t = 45$ h. This value was used as the colony-area uncertainty for downstream TTP robustness analyses, as illustrated in **a**. The black line indicates the median error and grey whiskers show the interquartile range.

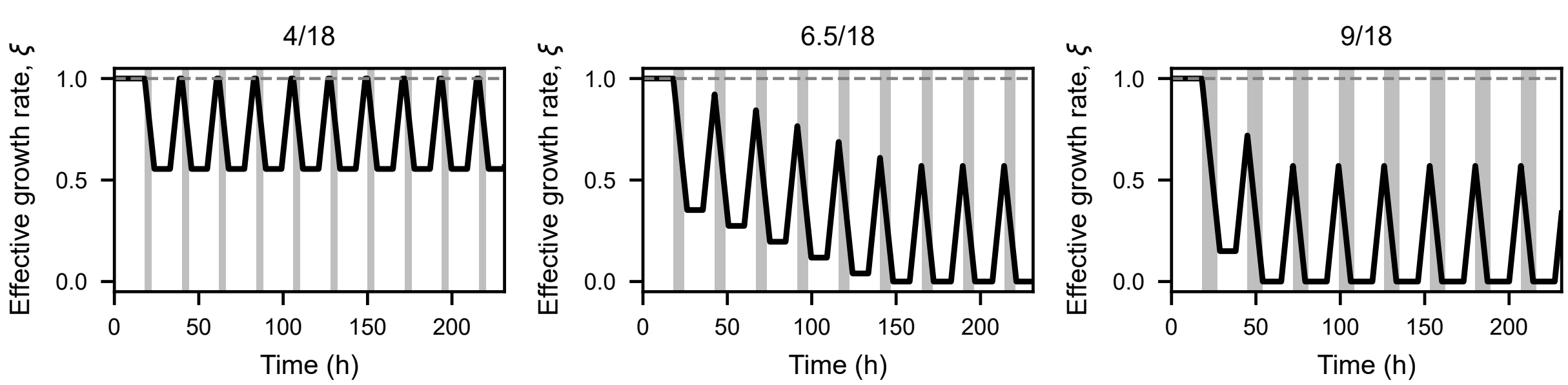

Supplementary Figure 11: Simulated effective growth rates ξ due to treatment for the intermittent treatments shown in Fig. 4. Gray shading indicates treatment.

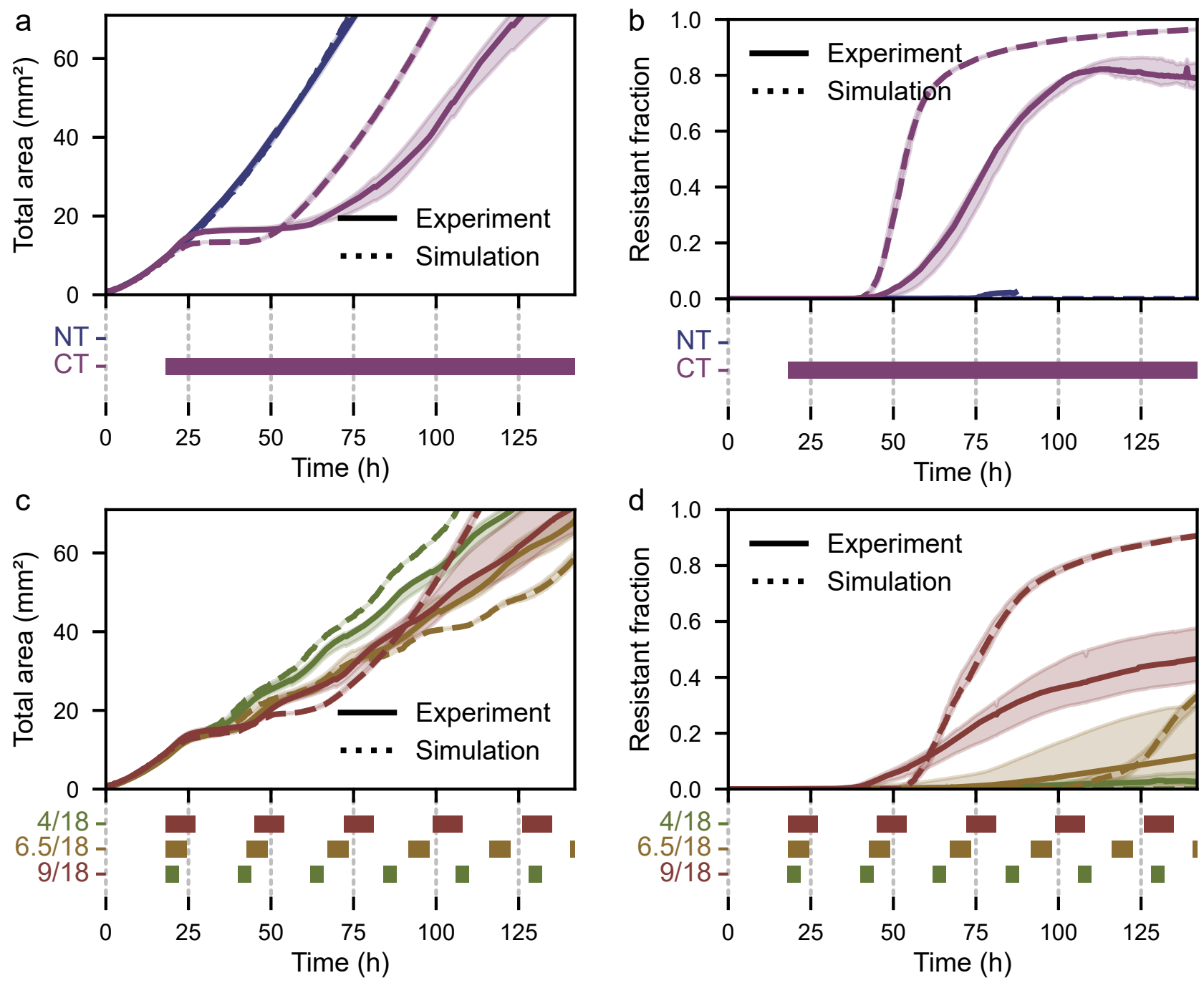

Supplementary Figure 12: **a-d** Direct comparison between the simulation and the experiment in total area and resistant fraction for the treatment schedules shown in Fig. 5. Lines indicate the median, shaded areas indicate the IQR. The sample size for the simulation is $n = 20$, the experiment sample size is the same as in Fig. 5. The colored bars indicate treatment.

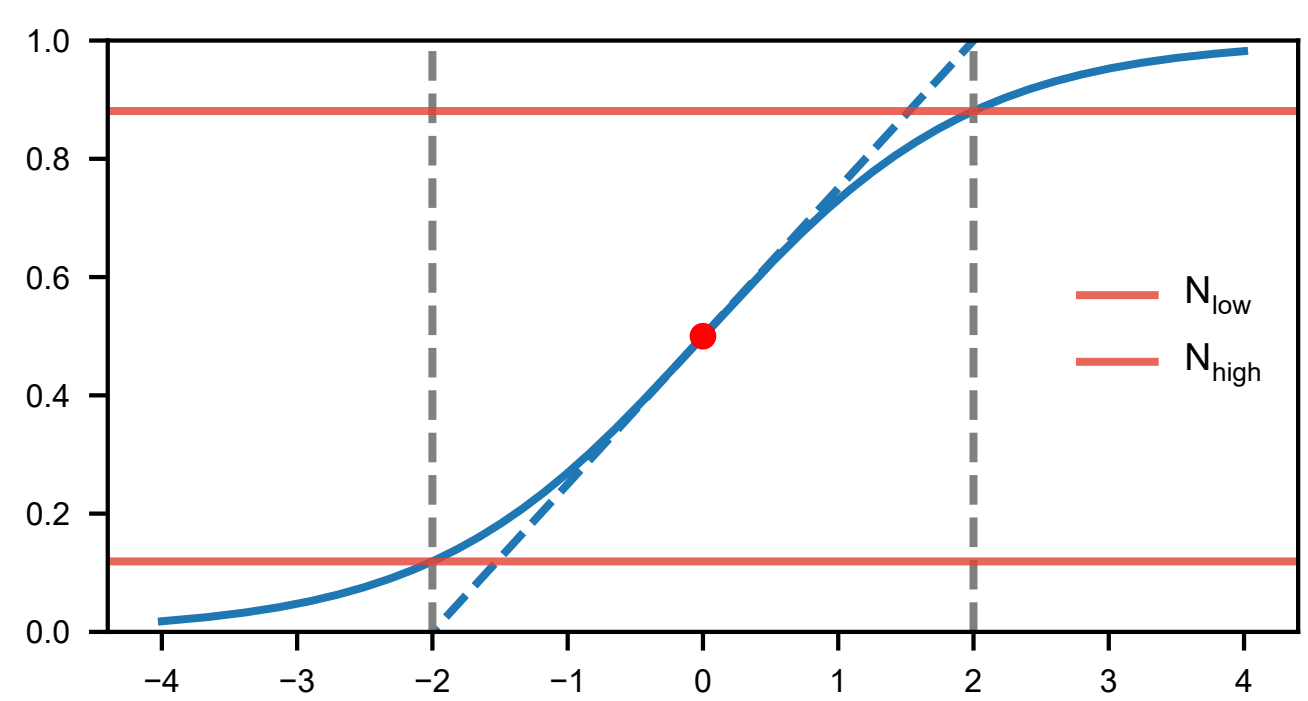

Supplementary Figure 13: Definition of the nutrient thresholds N_{low} and N_{high}, which define the position of λ_{low} and λ_{high}. The blue line is a sigmoidal function and the dashed blue line is a linear function with the slope of the sigmoid function at the red dot. The grey lines indicate the intersections of the linear function with 0 and 1.
