# Supplementary material for: A microbial therapy-mimicry assay shows how spatial resource dynamics control resistance escape
Source: Nat Ecol Evol. 2026 Sep 28;10(11):2160–73. doi: 10.1038/s41559-026-03178-z (PMC13736643; doi:10.1038/s41559-026-03178-z)
Supplement: Supplementary file 3 [file 41559_2026_3178_MOESM3_ESM.pdf]

nature portfolio

Peer Review File

A microbial therapy-mimicry assay shows how spatial resource dynamics control resistance escape

Corresponding Author: Dr Jona Kayser

Version 0:

Decision Letter:

18th March 2026

*Please ensure you delete the link to your author homepage in this e-mail if you wish to forward it to your co-authors.

Dear Jona,

Your manuscript entitled "Spatial resource dynamics control resistance escape" has now been seen by two reviewers, whose comments are attached. The reviewers have raised a number of concerns which will need to be addressed before we can offer publication in Nature Ecology & Evolution. We will therefore need to see your responses to the criticisms raised and to some editorial concerns, along with a revised manuscript, before we can reach a final decision regarding publication.

We therefore invite you to revise your manuscript taking into account all reviewer and editor comments. Please highlight all changes in the manuscript text file in Microsoft Word format.

We are committed to providing a fair and constructive peer-review process. Do not hesitate to contact us if there are specific requests from the reviewers that you believe are technically impossible or unlikely to yield a meaningful outcome.

When revising your manuscript:

* Include a “Response to reviewers” document detailing, point-by-point, how you addressed each reviewer comment. If no action was taken to address a point, you must provide a compelling argument. This response will be sent back to the reviewers along with the revised manuscript.

* If you have not done so already please begin to revise your manuscript so that it conforms to our Article format instructions at http://www.nature.com/natecolevol/info/final-submission. Refer also to any guidelines provided in this letter.

* Include a revised version of any required reporting checklist. It will be available to referees (and, potentially, statisticians) to aid in their evaluation if the manuscript goes back for peer review. A revised checklist is essential for re-review of the paper.

* Extended Data Figures - please ensure that any supplementary figures and tables that are crucial to the manuscript’s conclusions are converted into Extended Data figures and tables to increase visibility of these data. Extended Data figures and tables are online-only (present in the online PDF and full-text HTML versions of the paper), peer-reviewed display items that provide essential background to the article but are not included in the main article due to space constraints. A maximum of ten Extended Data display items (figures and tables) is permitted.

Please use the link below to submit your revised manuscript and related files:

Link Redacted

Note: This URL links to your confidential home page and associated information about manuscripts you may have submitted, or that you are reviewing for us. If you wish to forward this email to co-authors, please delete the link to your homepage.

We hope to receive your revised manuscript within four to eight weeks. If you cannot send it within this time, please let us know. We will be happy to consider your revision so long as nothing similar has been accepted for publication at Nature Ecology & Evolution or published elsewhere.

Nature Ecology & Evolution is committed to improving transparency in authorship. As part of our efforts in this direction, we are now requesting that all authors identified as 'corresponding author' on published papers create and link their Open Researcher and Contributor Identifier (ORCID) with their account on the Manuscript Tracking System (MTS), prior to acceptance. ORCID helps the scientific community achieve unambiguous attribution of all scholarly contributions. You can create and link your ORCID from the home page of the MTS by clicking on 'Modify my Springer Nature account'. For more information please visit please visit www.springernature.com/orcid.

Please do not hesitate to contact me if you have any questions or would like to discuss these revisions further.

We look forward to seeing the revised manuscript and thank you for the opportunity to review your work.

[redacted]

Reviewer expertise:

Reviewer #1: cancer evolution, evolution-based therapies

Reviewer #2: spatial cell interaction, computational and experimental

Reviewers' comments:

Reviewer #1 (Remarks to the Author):

see attached

Reviewer #2 (Remarks to the Author):

This manuscript reports experimental and computational work on how drug resistance evolves in spatially structured populations. It uses a yeast colony model where each colony starts from a single cell, resistance appears through an irreversible fluorescent switch, and resistant clones are tracked under defined treatment schedules. The main result is that pulsed treatment can alter resource availability and either allow resistant clones to expand or keep them confined. This mechanism may apply to other systems, including antibiotic resistance in biofilms and chemotherapy resistance in tumors. The study is promising, but should be revised before publication.

Main issues:
1. The current model calibration approach undermines independent validation. The digital twin is interesting, but the model is calibrated to experimental properties that are closely tied to the main claims, such as radial expansion, clone regrowth timing, mutation counts under continuous treatment, and breakout probabilities across pulse durations. As a result, this exercise remains explanatory rather than validative. The manuscript should separate fitting and test data more clearly, report parameter uncertainties, and demonstrate how uncertainty affects the inferred optimal ridge in schedule space. Additionally, the experimental validation of the predicted sweet spot is limited to three schedules at a single tau_off value. This result is encouraging, but it does not prove the existence of a sharply defined optimum.

2. The paper should reconsider the use of the term 'effective phase transition.' The data show a sharp crossover between confinement-dominated and escape-dominated regimes, but 'phase transition' is not fully supported. To use this term, the paper would need to define an order parameter and show that the boundary is robust to different analysis choices, such as the progression threshold and other fitted quantities. Otherwise, it would be more accurate to use terms like transition, boundary, or crossover.

3. The translational focus should be more cautious. The Abstract and Discussion mention relevance to adaptive cancer therapy, but the results are limited to a 2D yeast colony model and do not include features like cell killing or 3D structure. The mechanism may generalize, but the paper does not demonstrate this in tumors.

Minor issues:
1. The statistical comparisons in Fig. 5 should be clearer, with effect sizes and confidence intervals for the main schedule comparisons.
2. The robustness of time-to-progression to the area threshold and extrapolation method should be shown.
3. The manuscript and supplement also need careful copyediting (e.g., the abstract explicitly states "\textit{in silico}", suggesting a lack of proofreading).

*******************END*******************

Version 1:

Decision Letter:

17th July 2026

Dear Jona,

Thank you for submitting your revised manuscript "Spatial resource dynamics control resistance escape" (NATECOLEVOL-26020690A). It has now been seen again by the original reviewers and their comments are below. The reviewers find that the paper has improved in revision, and therefore we'll be happy in principle to publish it in Nature Ecology & Evolution, pending minor revisions to satisfy the reviewers' final requests and to comply with our editorial and formatting guidelines.

Please email us a copy of the file in an editable format (Microsoft Word or LaTex)-- we can not proceed with PDFs at this stage.

If you have not done so already, please ensure that you also email us a completed copy of the Reporting summary :

Reporting summary: https://www.nature.com/documents/nr-reporting-summary.pdf

We are now performing detailed checks on your paper and will send you a checklist detailing our editorial and formatting requirements in a couple of weeks. Please do not upload the final materials and make any revisions until you receive this additional information from us.

Thank you again for your interest in Nature Ecology & Evolution. Please do not hesitate to contact me if you have any questions.

[redacted]

Reviewer #1 (Remarks to the Author):

The authors have responded thoughtfully to the previous reviews, and the manuscript is much improved. The new experiments, statistical analyses, colony-based fitness controls, and model extensions all strengthen the work. I also appreciate the more careful distinction between the sharp boundary seen in the model and the somewhat softer experimental crossover. Overall, the paper is in very good shape, and I would be happy to support publication after a few minor revisions.

1. I would still tread lightly around "dynamic bifurcation" and "phase transition." The new analyses are suggestive and interesting, but in a finite, stochastic, expanding system they may not quite establish a formal bifurcation. "Phase-transition-like" or "sharp stochastic transition between trajectory classes" seems safer.

2. The front-growth ratio is a useful organizing idea, but gamma front star could be defined a little more clearly. My reading is that this is a system-dependent threshold that captures a potentially general ecological principle, rather than a universal constant. It would help to say that plainly.

3. The cytotoxic-treatment and spatial-gradient simulations are nice additions. Please keep a bright line between these model-based extensions and what has been directly demonstrated experimentally.

4. The adaptive-treatment result is fun and worth highlighting, but it should be introduced clearly as a proof-of-principle heuristic rather than an optimized control strategy.

5. The rebuttal does a good job distinguishing between experimentally locating the approximate sweet spot and experimentally proving the sharpness of the simulated boundary. I would carry that same caution consistently through the manuscript.

6. One final proofreading pass would be worthwhile to catch the remaining typographical and notation gremlins.

These are all fairly small points. The authors have addressed the substantive concerns from the first round, and the manuscript now tells a clearer, stronger, and more convincing story.

Reviewer #2 (Remarks to the Author):

Reviewer #2 (Remarks to the Author)

This revised manuscript reports an experimental and computational study on how drug resistance evolves in spatially

structured populations. As described in the original submission, the study uses a yeast colony model in which resistance can be tracked via an irreversible fluorescent switch and combines these experiments with a spatial computational model to study how intermittent treatment alters resource availability and resistant-clone escape.
The manuscript is substantially improved. The paper now more clearly separates model calibration and testing, adds uncertainty analyses for fitted parameters and the inferred schedule ridge, expands the experimental analysis of the intermittent-treatment optimum, qualifies the phase-transition language, adds robustness analyses for time-to-progression, and makes the translational discussion more cautious. In my view, this paper is ready for publication after minor editorial revisions.

Main issues:
1. Calibration and validation concerns have been addressed. The revision removed the pulse-duration-dependent breakout probabilities from the fitting procedure, and this version now estimates the cell-per-deme scaling from independent high-magnification images. It also reports parameter uncertainty, propagates this uncertainty through the schedule sweep, and adds leave-one-out cross-validation. The digital twin remains best interpreted as a mechanistic, explanatory model rather than a quantitative validator, because some fitted quantities remain closely related to the main biological dynamics. But the revised manuscript now states this limitation appropriately and tempers the claims about the exact prediction of the schedule optimum.
2. The experimental support for the treatment sweet spot is stronger and more appropriately framed. The revision has an increased sample size for the 6.5 h/18 h schedule, new comparisons around the optimum, reports effect sizes and confidence intervals, and adds experiments varying tau_off. These additions do not establish a globally sharp optimum across all schedule space, but the manuscript no longer claims that they do. The current data support the more nuanced claim that the tested schedules show a local optimum and a qualitative switch from confinement-dominated to escape-dominated dynamics.
3. The phase-transition language is more acceptable. The manuscript no longer presents the result simply as an "effective phase transition," but instead uses "dynamic phase-transition-like boundary" and explains that the analogy is to trajectory classes rather than to an equilibrium thermodynamic transition. The paper also defines the resistant fraction as an order-parameter-like observable and demonstrates its robustness to uncertainty in fitted parameters and to progression-threshold choice. This revision addresses a central concern, although readers should still understand the experimental evidence as supporting a sharp crossover rather than proving a formal bifurcation.
4. The translational framing is now more cautious. The revised Abstract and Discussion distinguish the general mechanism from direct tumor relevance, and the paper now notes limitations related to 2D geometry, reversible growth arrest, lack of cell killing in the main assay, drug penetration, and more complex tumor or biofilm structure. The added cytotoxic-treatment and spatial-treatment simulations are useful, and the paper correctly presents them as computational extensions rather than validation in tumor-like systems.

On the minor issues:
1. The revised Figure 5 statistics are much clearer, with schedule-to-schedule comparisons, effect sizes, and confidence intervals. I have no further major concerns about this point.
2. The robustness of time-to-progression to the progression threshold and out-of-frame interpolation is now shown in the supplement and described in the Methods. This addresses the previous concern.
3. The manuscript still needs copyediting. There are still a few small issues, such as missing spaces after periods (e.g., "pulse.Motivated" and "conclusions.As") and some incorrect wording ("this data"). These should be straightforward to fix.

INTERNAL USE ONLY AIP modules required:["Life Science"]

Open Access This Peer Review File is licensed under a Creative Commons Attribution 4.0 International License, which permits use, sharing, adaptation, distribution and reproduction in any medium or format, as long as you give appropriate credit to the original author(s) and the source, provide a link to the Creative Commons license, and indicate if changes were made.
In cases where reviewers are anonymous, credit should be given to 'Anonymous Referee' and the source.
The images or other third party material in this Peer Review File are included in the article's Creative Commons license, unless indicated otherwise in a credit line to the material. If material is not included in the article's Creative Commons license and your intended use is not permitted by statutory regulation or exceeds the permitted use, you will need to obtain permission directly from the copyright holder.
To view a copy of this license, visit https://creativecommons.org/licenses/by/4.0/

Response to the Reviewers

Appold and Citak et al.

We thank the reviewers for their thoughtful and constructive assessment of our manuscript. Their comments helped us clarify the interpretation of the treatment-schedule boundary, strengthen the statistical and modeling support, and make the translational framing more precise. Below, we respond point by point. Reviewer comments are reproduced in quoted text, followed by our responses. Each comment has a unique ID that is searchable in the supplied pdf with tracked changes. Where revisions overlap, we use the Reviewer 1 ID and cross-reference it in the Reviewer 2 response.

Reviewer 1

We thank Reviewer 1 for the positive assessment of the experimental platform, the digital-twin framework, and the mechanistic interpretation of intermittent treatment. We especially appreciate the suggestions to clarify the nature of the schedule-space transition, strengthen the validation of the fitness advantage in spatial colonies, quantify uncertainty, and more explicitly discuss translational limitations.

Key Results and Significance

> This manuscript by Appold and colleagues addresses the important open question of how the spatial structure of a growing population shapes resistance evolution under therapy, and whether that structure can be exploited therapeutically. The authors build a yeast based experimental platform in which colonies expand from single founder cells under programmable intermittent temperature shifts that mimic therapy. Resistant mutants arise stochastically and are fluorescently trackable in real time, providing full spatio-temporal trajectories of individual resistant clones across hundreds of generations.
>
> The central finding is that therapy-induced suppression of sensitive cell growth allows nutrients to redistribute toward the population bulk, releasing resistant mutants born in the bulk from resource mediated confinement. When therapy is paused, sensitive cells rebound at the colony front, re-establishing nutrient depletion in the bulk thus confining resistant clones. The authors then use a computational digital twin to scan a large space of treatment schedules and identify a sharp phase transition separating sensitivity dominated control from resistance driven failure. Schedules below a critical treatment duration maintain approximately Markovian cycle-to-cycle dynamics, while schedules above it produce a drift toward resistance. The authors experimentally validate this predicted sweet spot, finding that a 6.5h on, 18h off schedule maximizes time to progression while maintaining a low resistant fraction, consistent with model predictions.
>
> This work offers a mechanistic explanation for why intermittent therapy clinical trials have underperformed despite strong preclinical rationale. They find that the therapeutic sweet spot is narrow, biphasic, likely varying, and easily missed. The suggestion that growth rebound during treatment pauses could serve as a clinically accessible measure of confinement potential is an intriguing and testable idea. The real-to-sim-to-real framework is well executed and represents a significant methodological contribution.

We thank the reviewer for this generous and careful summary of the manuscript. We are pleased that the reviewer found the experimental platform, resistance-tracking approach, and real-to-sim-to-real framework compelling. In the revised manuscript, we have preserved the central mechanistic narrative while sharpening the terminology around the schedule-space boundary and making the translational claims more cautious, as discussed in detail below.

Data Validity, Methodology, and Analytical Approach

> The experimental system is well-designed and appropriate for the questions being asked. The genetically engineered yeast strain with a synthetic, irreversible recombinase based mutation switch decouples resistance acquisition from confounding fitness costs and enables reliable fluorescent tracking. Single-cell seeding ensures tractable lineage analysis, and the arrayed colony format provides reasonable throughput for imagining.

R1.C01 - Sample Size and Sharpness of Schedule Boundary

> Colony numbers are limited for some conditions (8-13 colonies per condition), which limits statistical power. The Mann-Whitney result for the key 6.5h condition is statistically significant compared to the control (CT), but other pairwise comparisons (between other experimental treatment schedules and the optimal one) are apparently not statistically significant (Fig. 5b). Given that the central claim rests on a sharp boundary in schedule space, the experimental evidence for that sharpness is not fully convincing at these sample sizes, as a result.

We thank the reviewer for raising this important point. In the initial manuscript, Fig. 5b reported significance only relative to continuous treatment, which demonstrated that intermittent treatment could outperform CT but did not directly quantify whether neighboring schedules differed from the apparent optimum.

We have revised this analysis and presentation. First, we now report the relevant strategy-to-strategy comparisons, including comparisons between the optimal schedule and adjacent intermittent schedules. Second, we have conducted additional experiments, improving colony numbers.

We want to emphasize that we expect the transition in experiments to be potentially much smoother than the simulated boundary because the assay contains additional biological and physical complexities not included in the minimal digital twin, such as collective cell motion or more complex treatment response kinetics. We therefore now distinguish more clearly between the sharp transition predicted in the model and the experimentally observed schedule-dependent change in TTP and resistant fraction. The revised text presents the experiments as supporting the existance and approximate location of a rapid transition in schedule space that is associated with a therapeutic optimum, while being appropriately cautious about the precision with which boundary sharpness can be inferred from the given data.

R1.C02 - Extrapolation Uncertainty

> The image analysis pipeline is a strength of the work. Machine learning based segmentation via Ilastik is well-validated for this type of data, and the clonal tracking methodology is clearly described with reasonable classification criteria. The extrapolation approach for colonies growing beyond the imaging field of view is reasonable, though it introduces non-trivial uncertainty at later timepoints that, though mentioned, could be better quantified.

We agree that the uncertainty introduced by our estimations of late-stage out-of-frame growth should be quantified more clearly. We have therefore expanded the Methods section describing time-to-progression and area estimation.

Note that our estimates are interpolations rather than extrapolations, as incorrectly stated in the original manuscript. Once a colony reaches the edge of the time-lapse field of view, the last trusted in-frame measurement is connected to a lower-magnification endpoint image acquired after the time lapse. Thus, the procedure is a direct linear interpolation between the final fully trusted time-lapse frame and an experimentally measured endpoint.

To make the effect of this procedure transparent, we have added a supplementary uncertainty analysis showing how the interpolation affects colony area, resistant area, and the derived TTP values (Supplementary Fig. 10). The data shows that the uncertainty added by the interpolation procedure is far below that of colony-to-colony variation. This analysis is now referenced from the Methods section so that readers can assess how much late-stage field-of-view truncation contributes to the conclusions.

R1.C03 - Therapy-Response Sensitivity Analysis

> The digital twin simulations are minimal, and intentionally so, capturing the essential nutrient growth coupling with few parameters fitted directly from experimental data. This makes it mechanistically interpretable, which is appropriate. The parameter fitting is described in sufficient detail in the supplementary material, and the qualitative agreement between simulation and experiment is convincing. The authors are appropriately forthcoming that quantitative predictions from the schedule sweep should be treated as rough guides. However, the sensitivity of the phase boundary to therapy response kinetics as shown in Supplementary Fig. 4 is substantial and should likely be presented in the main text rather than the supplement, as it directly impacts the robustness of the paper's central finding.

We agree that the sensitivity of the schedule boundary to therapy-response kinetics is central to the interpretation of the model and should be more visible than it was in the original manuscript. The detailed parameter sweep remains in the Supplementary Information because it contains several conditions and would be difficult to include in full in the main figure set. However, we now summarize its key result in the main text by adding the new panel Fig. 4d. This plot shows how changes in treatment-response kinetics alter the position and shape of the optimal ridge in schedule space, including an improved parameter uncertainty analysis.

In the accompanying text, we state more explicitly that the precise location of the sweet spot is not a universal number, but is governed by treatment response kinetics. This revision makes the sensitivity analysis part of the main argument while preserving the full sweep as supporting detail.

R1.C04 - Cytostatic Versus Cytotoxic Therapy

> A notable limitation is that the model and assay employ reversible growth inhibition rather than cell death, which is the mechanism of action for many clinically relevant therapies, particularly for cancer.
>
> The authors appropriately acknowledge this in the discussion, but the implications warrant further discussion. Whether the phase transition behavior and the confinement mechanism are preserved

> when the therapy induces cell death rather than growth arrest is an important question for clinical applicability.

(Note: The comment below was further down in the original remarks but we included it here to facilitate a consistent response)

> **3. Robustness of the confinement argument in cytotoxic therapies?** Could the authors delve a bit into how using a cytotoxic therapy rather than a cytostatic might change their results? Cell-killing therapies could alter spatial dynamics by: creating voids within the colony bulk changing local nutrient flux or altering competition dynamics between sensitive and resistant clones as is seen in cancer in the presence of targeted therapies. It would therefore be useful for the authors to comment on whether the predicted schedule sweet spot is expected to persist under cytotoxic rather than cytostatic therapies, or whether additional modeling would be required. A further discussion about spatial dynamics in heterogeneous tumors/biofilms could be useful as edge effects could dominate and also modify confinement dynamics.

We agree that reversible growth inhibition is an important limitation of our yeast-based model system, especially when considering translation to cytotoxic cancer therapies. We have revised the Discussion to treat this distinction more explicitly and to avoid implying that the present experiments directly demonstrate cytotoxic therapy behavior.

Mechanistically, we expect cytotoxic therapy to change the rebound phase after treatment cessation. In our assay, sensitive cells that are growth-arrested during treatment can later resume proliferation and re-establish a confining front. If therapy instead kills sensitive cells, fewer cells remain available to rebuild that front, local voids may alter nutrient flux, and resistant clones may have a longer window to reach the edge. Thus, cytotoxicity should generally shift the system toward easier resistance escape, particularly for longer treatment-on intervals.

To test this expectation in the modeling framework, we implemented therapy-induced sensitive-cell death as an additional simulation scenario (Extended Data Fig. 6). These simulations indicate that the same qualitative schedule-dependent boundary can persist when treatment is cytotoxic, but that it shifts toward shorter treatment durations as killing strength increases until any treatment causes competitive release. We now present these results in a new Extended Data Figure and discuss them as a model-based extension, not as an experimentally validated claim, in the Discussion and Methods sections. The revised manuscript therefore frames cytotoxic treatment as a key context in which the confinement mechanism may persist only when enough sensitive cells survive to rebuild the competitive barrier.

Major

R1.C05 - Spatially Graded Treatment Field

> **Major Remark 1: Spatially homogeneous treatment is a fundamental limitation that can be explored by further simulations.** Treatment in the assay is applied as a spatially uniform temperature change, which is a significant departure from how drug or treatment gradients arise in treatment of biofilms and solid tumors, where penetration likely decreases with depth from the edge of the population. This means the bulk of the population, where confined resistant mutants reside, may in reality receive substantially less treatment than the leading edge, potentially altering or eliminating the resource redistribution dynamics that are central to the paper's findings. While relaxing this experimentally may not be feasible or is outside the scope of this work, the authors

> could and perhaps should explore a spatially graded treatment field in their simulations, with treatment intensity highest at the colony periphery and decreasing toward the bulk. If such a gradient shifts or eliminates the therapeutic sweet spot, this would greatly change the interpretation of the findings and their translational relevance. This limitation should also receive more explicit discussion alongside the others which the authors identify.

We thank the reviewer for this suggestion. We agree that spatially homogeneous treatment is an important simplification relative to many biofilms and solid tumors, where treatment penetration can decrease with distance from the exterior. As suggested, we have therefore extended the simulation framework to include spatially graded treatment fields in which treatment intensity is highest near the population periphery and decreases toward the bulk (Extended Data Fig. 7).

In these simulations, the therapeutic sweet spot remains. However, the shape of the optimal ridge changes in position and magnitude with steeper gradients facilitating confinement. The observed stability, especially for the treatment pause durations probed in this work, can be understood by realizing that the bulk of the population is already largely growth-limited by nutrient depletion. Even under spatially homogeneous treatment, the strongest relative effect of therapy occurs in the proliferative peripheral region, where sensitive cells are actively consuming nutrients. A peripheral treatment gradient therefore changes the imposed field but does not eliminate the basic mechanism by which suppressing edge growth redistributes resources inward and can release bulk-born resistant clones.

We now include these simulation results in the Supplementary Information and discuss the implications and limitations in the main text. In particular, we emphasize that this analysis does not replace experiments with true drug gradients, but it shows that the confinement mechanism is not trivially dependent on perfectly homogeneous treatment.

R1.C06 - Phase Transition Terminology

> **2. Exploration of the phase transition.** The manuscript repeatedly describes a phase transition separating resistance escape from control under different treatment schedules. However, it remains unclear whether this transition reflects: a true bifurcation in the underlying ecological dynamics; or a smooth threshold behavior arising from resource redistribution timescales. Given the relatively small number of colonies per condition and the stochastic nature of mutation emergence, the experimental evidence for a sharp boundary appears limited. It would strengthen the manuscript to clarify: whether the digital twin predicts a true dynamical bifurcation, or whether the transition is simply a rapid but continuous change in failure probability. If the former, identifying the underlying control parameter and demonstrating the bifurcation explicitly would greatly strengthen the theoretical contribution. This makes me think a bit about John Drake's work on stochastic extinctions (or outbreaks) and the changes in variance before them (in time, yes, but maybe similar approaches could be taken spatially).

We agree that our original use of "phase transition" required clearer qualification. The populations studied here are expanding, stochastic, non-equilibrium systems, and we do not intend to claim a canonical thermodynamic phase transition. We have revised the manuscript to state this explicitly and to distinguish between the model prediction of a sharp dynamic boundary and the experimentally observed schedule-dependent crossover, which is smoothed by biological variability and other effects not captured by the simulation.

Motivated by the reviewer's comment, including the connection to work by John Drake and colleagues on dynamic transitions in population trajectories, we now analyze the transition in terms of trajectory classes rather than equilibrium phases. Using the endpoint resistant fraction as an integrating, trajectory-level order-parameter-like observable, we find strong evidence for a dynamic bifurcation in simulations, including increased standard deviation, skewness, and Binder cumulant near the boundary (Extended Data Fig. 5). Importantly, this analysis indicates that the underlying mechanism does not solely reside in the Markovian/non-Markovian transition or the resulting drift in the cycle-averaged treatment effect. Instead, it can be traced to a critical peripheral expansion rate (also see our response to comment R1.C07 below and Extended Data Fig. 3). We now explicitly show the resulting bimodal distribution of trajectories around this boundary (Extended Data Fig. 4).

We have also performed additional experiments to address the issue of limited colony number, especially at the optimal schedule. Closer inspection of these data suggests that trajectory bimodality in the resistant fraction may also be present experimentally. However, while corroborative, this evidence should be regarded as circumstantial at the resolution of our assay.

We therefore now present our observations as a dynamic phase-transition-like boundary in schedule space, rather than as an effective phase transition without further qualification, and support this interpretation with quantitative evidence. Where appropriate, the manuscript explains that the simulations support the notion of a dynamic phase transition, while the experiments validate the predicted direction and approximate location of the boundary without proving a formal bifurcation.

R1.C07 - What Governs the Confinement Break

> **4. What truly governs the confinement break?** The manuscript identifies a sharp boundary in treatment schedule space separating resistance escape from control. However, the mechanistic explanation provided suggests that this boundary arises from a competition between ecological timescales governing nutrient redistribution and front regrowth. It would strengthen the conceptual contribution of the work if the authors could identify the dimensionless parameter governing this transition and demonstrate that the observed schedule sweet spot reflects a more general ecological control principle rather than a system-specific optimization.

We thank the reviewer for pushing us to make the governing principle more explicit. We have revised the manuscript to separate two coupled mechanisms that govern confinement and escape dynamics in our system (also see above response to R1.C06).

The first is schedule-dependent: the choice of intermittent schedule changes whether sensitive growth at the population edge can fully recover during the treatment pause. When recovery is complete, consecutive cycles remain approximately independent so that average sensitive cell growth remains stable and sufficient to maintain confinement. When recovery is incomplete, the system accumulates a cycle-to-cycle drift in sensitive cell growth, leading to a gradual slowdown and eventually loss of confinement. This approximately Markovian to non-Markovian transition had already been described in the original manuscript.

Having identified drifting sensitive-cell growth as a key factor, we then asked whether the transition also persists for non-cycling treatments of different strengths. To this end, we simulated constant-treatment conditions over a range of effective sensitive-cell growth rates, revealing a second underlying transition that is schedule-independent (Extended Data Fig. 3). We found that confinement is maintained as long as the treatment-reduced sensitive growth rate remains above a critical threshold $\gamma^*_{\mathrm{front}}$. This threshold is

set by the minimal sensitive-front growth needed to reduce nutrient availability around resistant clones sufficiently to offset their resistance-mediated advantage.

We therefore identify the front-growth ratio $\gamma_{\text{front}}/\gamma^{*}_{\text{front}}$ as the underlying dimensionless parameter governing the observed crossover and suggest that this nutrient-depletion threshold constitutes a more general ecological control principle in spatially structured, range-expanding populations.

R1.C08 - Fitness Advantage in Colony Assay

> **5. Experimental validation of fitness advantage in the colony assay.** Growth rates of the sensitive and resistant strains are measured in liquid culture (Fig. 1e), but it does not appear that the fitness difference was confirmed under colony expansion conditions. We suggest the authors measure the radial expansion rate of 100% sensitive and 100% constitutively resistant colonies on solid agar at 30°C and 35°C to confirm that liquid culture growth rates correspond to the fitness advantage expressed during the colony assay, if possible. A competition experiment using a mixed inoculate of the two strains, for instance, in a planar geometry would further strengthen this control. At 35°C, the opening angle of sectors formed by the resistant strain can be used to quantify the fitness advantage directly, providing an independent validation consistent with methods previously employed in the relevant experimental literature.

We agree that the fitness difference between sensitive and resistant cells should be explicitly validated in the spatial colony context, not only in liquid culture. Although liquid-culture growth rates establish the intended temperature-dependent phenotype of the engineered strains, front expansion in colonies can reflect additional collective effects, including local crowding, nutrient gradients, and growth-induced motion.

In principle, the type-specific front velocities measured in our experiments already allow for a direct on-plate comparison at different temperatures, confirming neutral expansion at 30°C and a full stop of sensitive cells at 35°C (see e.g. kymographs in Fig. 2).

Following the reviewer's excellent suggestion, we have now added dedicated in-colony competition assays comparing sensitive and resistant populations on solid agar at 30°C, 31°C, 32°C, and 35°C, including both circular-colony and linear-front geometries. While angle-based measurements become unreliable for extreme fitness differences due to lateral expansion, these measurements corroborate the behavior observed in liquid culture. We report the new analysis in the revised Methods and in a new panel in the Extended Data Fig. 1 together with our previous liquid culture measurements.

R1.C09 - Supplementary Fig. 5 in Main Text

> **6. Supplementary Fig. 5 belongs in the main text.** The sensitivity of the phase boundary to therapy response kinetics is shown in Supplementary Fig. 5 and is substantial. Given that the location of the sweet spot is the paper's central quantitative prediction, this sensitivity analysis should be made more visible to readers to evaluate the main claims.

We agree and have addressed this together with the related comment on therapy-response sensitivity above (comment R1.C03). Because the full Supplementary Fig. 5 (now Supplementary Fig. 4) contains a detailed multi-parameter sweep, moving it in its entirety would make the main figure overly dense. Instead, we now promote the central result of this analysis into the main text as a new summary panel in Fig. 4.

The added panel shows how treatment-response kinetics shift the optimal ridge in schedule space, including propagated parameter uncertainties. The accompanying text now makes clear that the location of the sweet spot depends strongly on response kinetics and should therefore be interpreted as a system-specific prediction rather than a universal schedule. The full sensitivity sweep remains in the Supplementary Information, where readers can inspect the underlying parameter dependence in detail.

R1.C10 - Supplementary Fig. 10 in Main Text

> **7. Supplementary Fig. 10 warrants inclusion in the main text.** The adaptive therapy result in Supplementary Fig. 10 is very interesting and arguably the most clinically relevant finding in the paper. It demonstrates that 'closed loop' treatment decisions outperform the optimized fixed schedule. It would serve this work well to include in the main text.

We are delighted that the reviewer shares our enthusiasm for the adaptive therapy results and agree that they are relevant for the broader interpretation of the work. We have therefore promoted the closed-loop adaptive therapy experiment from the Supplementary Information into the main text and integrated it into the revised Fig. 5, moving the NT control data to the new dedicated Supplementary Fig. 7 to accomodate the adaptive therapy panels.

At the same time, we now discuss this result with appropriate caution. The adaptive strategy was designed as a proof-of-principle extension of the confinement mechanism, abstracting the learned insights into a simple heuristic, not as a fully optimized control policy. We therefore present it as evidence that population-level observables, such as the rate of population expansion, can in principle guide improved treatment decisions, while noting that a systematic exploration of adaptive control strategies is beyond the scope of the present manuscript.

Minor

R1.C11 - Fix Formatting Error

> Ln. 18: LaTeX formatting error: in silico should be italicized.

Thank you for catching this formatting error. We have corrected the abstract.

R1.C12 - Fix Typo

> Ln. 851: "an linear" should be "a linear".

Thank you for pointing this out. We have corrected "an linear" to "a linear" in the revised manuscript.

Clarity and Context

R1.C13 - Translational Parallels

> The manuscript is well-written and the figures are effective. The real-to-sim-toreal framing is clearly communicated. The translational parallels drawn to solid tumors and biofilms are plausible but somewhat broad given the mechanistic differences between the model system and those contexts. The authors would benefit from either qualifying these comparisons more carefully or providing explicit argument for why boundary diffusion nutrient dynamics in a 2D yeast colony capture the relevant physics of, a poorly vascularized fast growing tumor, for example.

We agree that the translational framing needed to be more precise. We have revised the Introduction and Discussion to more clearly separate what our yeast colony assay directly demonstrates from what may plausibly generalize to tumors and biofilms.

The central feature that we expect to generalize is the coupling between spatial resource gradients, local competition, and treatment-modulated growth. Many dense biofilms and poorly vascularized solid tumors contain spatial gradients in nutrients, oxygen, and proliferation state. For that reason, the principle that treatment can transiently reshape the spatial region in which resistant cells can grow may be relevant beyond the yeast system.

However, we now state the limitations more explicitly. Tumors and many biofilms can include three-dimensional architecture, active migration, heterogeneous microenvironments, immune and stromal interactions, multiple lesion sites, spatially nonuniform drug exposure, and therapies that kill cells rather than reversibly arresting growth. These features may shift, smooth, or even remove the schedule boundary observed in our controlled assay. To begin testing the robustness of the mechanism to two of these limitations, we extended the simulations to include spatial treatment gradients and therapy-induced sensitive-cell killing (see above responses to R1.C04 and R1.C05). These simulations are not experimental validation in tumor-like or biofilm-like systems, but they provide a sensitivity analysis showing that the qualitative confinement mechanism can persist while the boundary shifts. The revised manuscript therefore presents the tumor and biofilm parallels as mechanistic hypotheses and motivation for future work, not as direct evidence that the same quantitative schedules apply in clinical settings.

References

> The manuscript references the relevant prior literature appropriately, including the adaptive therapy literature and key spatial evolutionary biology work.

We thank the reviewer for this assessment. We have retained the relevant adaptive therapy and spatial evolutionary biology context and have added or emphasized additional literature where it helps clarify the dynamic-transition framing and the limitations of translating from our 2D yeast colony system to tumors and biofilms.

Reviewer 2

We thank Reviewer 2 for the constructive assessment and for identifying several places where our validation claims, terminology, and translational framing could be made more precise. We have revised the manuscript accordingly, as detailed below.

> This manuscript reports experimental and computational work on how drug resistance evolves in spatially structured populations. It uses a yeast colony model where each colony starts from a single cell, resistance appears through an irreversible fluorescent switch, and resistant clones are tracked under defined treatment schedules. The main result is that pulsed treatment can alter resource availability and either allow resistant clones to expand or keep them confined. This mechanism may apply to other systems, including antibiotic resistance in biofilms and chemotherapy resistance in tumors. The study is promising, but should be revised before publication.

We thank the reviewer for this clear summary and for recognizing the promise of the study. We agree that the manuscript required revision before publication, particularly in how we separated model calibration

from validation, qualified phase-transition language, and framed relevance to tumors and biofilms. The revised manuscript directly addresses these points while preserving the central experimentally grounded mechanism.

R2.C01 - Calibration, Validation, Uncertainty, and Optimal Ridge

> 1. The current model calibration approach undermines independent validation. The digital twin is interesting, but the model is calibrated to experimental properties that are closely tied to the main claims, such as radial expansion, clone regrowth timing, mutation counts under continuous treatment, and breakout probabilities across pulse durations. As a result, this exercise remains explanatory rather than validative. The manuscript should separate fitting and test data more clearly, report parameter uncertainties, and demonstrate how uncertainty affects the inferred optimal ridge in schedule space. Additionally, the experimental validation of the predicted sweet spot is limited to three schedules at a single tau_off value. This result is encouraging, but it does not prove the existence of a sharply defined optimum.

We thank the reviewer for identifying these important points. They prompted us to improve the manuscript in four main ways: i) a clearer separation of fitting and test data with reduced dependencies, ii) a robust analysis of parameter uncertainties and their downstream effects, iii) a more precise discussion of how the digital twin should be interpreted in relation to the experiments, and iv) additional experiments and analysis of the optimum in the yeast colony assay. We discuss each of these points in detail below.

To reduce circularity, we have removed pulse-duration-dependent breakout probabilities from the fitting procedure. In the original version, these data were used to estimate the cells-per-deme scaling factor, which is closely related to the later intermittent-therapy validation. In the revised analysis, this scaling is instead determined independently from high-magnification colony images (Supplementary Fig. 1d). Model calibration is restricted to independent measurements, including no-treatment radial expansion, treatment-response delays inferred from sensitive-colony radial growth, and mutation counts under continuous treatment. We explicitly state that breakout probability, time to progression, endpoint resistant fraction, and the location of the intermittent-schedule optimum are not used for model calibration.

We have also added uncertainty analyses around the fitted model. Because the original Nelder-Mead fitting procedure does not directly provide parameter uncertainty, we now estimate uncertainty through bootstrapping and report the resulting parameter ranges (see Supplementary Tab. 1). We then propagate these fitted-parameter uncertainties through the schedule sweep to assess how they affect the inferred optimal ridge in treatment-schedule space (Fig. 4d). In addition, because the amount of experimental data is limited, we use leave-one-out cross-validation as a practical way to separate fitting and test observations as far as possible within the available dataset (see Supplementary Tab. 2). Also see R1.C03 for the main-text treatment-response sensitivity analysis.

We want to emphasize that the digital twin is a mechanistically interpretable, coarse-grained model designed to test whether a minimal set of spatial dynamics is sufficient to reproduce the observed classes of behavior. It is not intended as a fully quantitative predictor of exact experimental outcomes. We now clarify this limitation more explicitly in the main text while also softening the interpretation of the experimental validation. The revised manuscript makes clear that the three experimentally tested schedules at a fixed off-treatment interval provide a targeted test of the model-predicted mechanism and

of the local optimum near the predicted ridge, but do not by themselves prove a globally sharp optimum throughout schedule space. Where appropriate, we now describe the simulations as identifying a candidate optimum and the experiments as supporting the predicted qualitative regime switch. Also see R1.C01 and R1.C06 for the corresponding changes to boundary sharpness and model-versus-experiment language.

Finally, we have conducted additional experimental runs at the optimal schedule (6.5h/18h) to improve colony number. We also performed experiments with varying τ_{off} values (6.5h/15.5h, 6.5h/20.5h, and 9h/20.5h; Supplementary Fig. 8). These results suggest that, in experiments, cycle-averaged sensitive growth may remain comparatively stable across changes in τ_{off} because recovery during the treatment pause seems to feed back on the effect of the subsequent treatment period, a feature not captured by the digital twin. Consistent with the proposed confinement mechanism, this stability is also reflected in TTP. Increasing τ_{on} (9/20.5) reduces the level of this growth plateau, facilitating resistant escape. While this observation is interesting, a systematic investigation of this phenomenon is beyond the scope of the present work. See also R1.C10 for how this growth-rebound logic motivated the adaptive-treatment analysis.

R2.C02 - Reconsider Effective Phase Transition

> 2. The paper should reconsider the use of the term 'effective phase transition.' The data show a sharp crossover between confinement-dominated and escape-dominated regimes, but 'phase transition' is not fully supported. To use this term, the paper would need to define an order parameter and show that the boundary is robust to different analysis choices, such as the progression threshold and other fitted quantities. Otherwise, it would be more accurate to use terms like transition, boundary, or crossover.

We thank the reviewer for this important clarification. We agree that our original use of "effective phase transition" was insufficiently qualified and could be read as claiming a formal thermodynamic phase transition, which is not what the current data establish. The revised manuscript therefore uses more cautious language throughout, referring primarily to a dynamic phase-transition-like boundary in schedule space. Where phase-transition language is retained, we now explicitly define it as an analogy to nonequilibrium dynamical transitions between trajectory classes, not as evidence for an equilibrium phase transition with asymptotic scaling. Also see R1.C06 and R1.C07 for the parallel revisions prompted by Reviewer 1.

We have also made the relevant trajectory-level observable explicit. In our system, the practical order-parameter-like quantity is the resistant fraction, or equivalently the classification of trajectories as sensitive-dominated versus resistance-dominated. This quantity changes sharply near the optimal ridge in the simulations, while the cycle-averaged sensitive front growth rate shows the accompanying shift from approximately history-independent recovery to history-dependent loss of reconfinement.

To address robustness, the revised analysis tests whether the optimal ridge is preserved under changes in fitted parameters (Fig. 4d) and under changes in the progression threshold used to define time to progression (Supplementary Fig. 9). These analyses are now discussed alongside the schedule sweep, and the limitations are stated more directly.

In response to the reviewer's question of whether the boundary may reflect a smooth threshold rather than a true dynamical transition, we investigated the underlying mechanism in more detail. This analysis suggests that the apparent boundary in intermittent schedule space combines two superimposed

transitions. First, treatment cycles can shift from approximately Markovian recovery, in which sensitive front growth is restored after each pause, to history-dependent incomplete recovery, in which growth suppression accumulates across cycles. Second, by studying constant-treatment simulations with different treatment strengths, we identified a schedule-independent confinement threshold governed by the relative growth of sensitive and resistant cells at the front. Confinement is maintained only while treatment-reduced sensitive front growth remains sufficiently high relative to resistant growth. Near this threshold, simulated trajectories separate into sensitive-dominated and resistance-dominated outcomes (Extended Data Fig. 3), producing bimodality in the endpoint resistant fraction (Extended Data Fig. 4, 5) and supporting the interpretation of a true dynamic bifurcation in the model. Thus, the intermittent-treatment boundary is not only a consequence of the chosen schedule grid, but reflects a more general ecological control principle in which sensitive cells must maintain a minimal average growth to contain resistant clones. This finding and the associated data are now presented in new dedicated extended data figures and discussed in the main text (also see R1.C07).

While we find strong evidence of a true dynamic bifurcation in simulations, we acknowledge that in the experimental data the available number of tested schedules and colonies supports a crossover in resistance fraction, including an optimum in TTP, but does not prove a formal bifurcation. To avoid overclaiming, the revised manuscript therefore presents the transition language as a useful mechanistic framework for understanding the switch from sensitive-dominated to resistance-dominated trajectories.

R2.C03 - Cautious Translational Focus

> 3. The translational focus should be more cautious. The Abstract and Discussion mention relevance to adaptive cancer therapy, but the results are limited to a 2D yeast colony model and do not include features like cell killing or 3D structure. The mechanism may generalize, but the paper does not demonstrate this in tumors.

We agree with the reviewer that a more cautious translational framing is warranted, given the intentionally reductionist approach of this study. The revised manuscript therefore makes clear that our results do not demonstrate the mechanism in tumors and should not be interpreted as a direct clinical prescription.

At the same time, the yeast system was chosen because it isolates a physical and ecological mechanism that may be relevant across spatially structured populations: therapy can alter where growth occurs, thereby reshaping resource availability and changing whether resistant subclones remain confined or escape. We have revised the Abstract and Discussion to distinguish this general mechanistic principle from its possible clinical relevance. In particular, we now state that the mechanism may generalize to other resource-limited structured populations, but that this remains a hypothesis requiring validation in more realistic tumor and biofilm models.

To further address the specific limitations pointed out by the reviewer, we have added simulations exploring therapy-induced cell killing and spatially graded treatment fields. These analyses are presented as extensions that test the sensitivity of the mechanism, not as substitutes for tumor experiments. We also expanded the limitations section to discuss how cytotoxicity, 3D geometry, spatial treatment penetration, and heterogeneous tumor growth could shift or weaken the schedule optimum, and why future work in 3D cancer organoids, matrix-producing biofilms, or other experimentally tractable 3D systems will be needed. See also responses to R1.C04 (cytotoxic treatment), R1.C05.(spatially graded treatment), and R1.C13 (more cautious translational language)

R2.C04 - Fig. 5 Statistics

> 1. The statistical comparisons in Fig. 5 should be clearer, with effect sizes and confidence intervals for the main schedule comparisons.

We thank the reviewer for this suggestion. We agree that the original Fig. 5 statistics were too compressed, because reporting only selected Mann-Whitney comparisons against continuous treatment did not sufficiently quantify the magnitude and uncertainty of the schedule-to-schedule differences.

In the revised Fig. 5, we now additionally report effect sizes and confidence intervals for the main treatment-schedule comparisons, with emphasis on comparisons across the predicted optimum rather than only comparisons against continuous treatment. This makes the experimental support for the local optimum more transparent and allows readers to distinguish the direction and magnitude of the observed effects from the statistical uncertainty.

We have also revised the text to avoid implying that the experimental data alone establish a globally sharp schedule boundary. Instead, the updated wording states that the experiments support the qualitative behavior predicted by the simulations in the tested region of schedule space: delayed progression near the predicted optimum, maintained low resistant fraction below the boundary, and increased resistant expansion for longer treatment-on durations.

See also R1.C01 for a similar comment of Reviewer 1 and associated improvements in the revised manuscript.

R2.C05 - Robustness to Threshold and Extrapolation

> 2. The robustness of time-to-progression to the area threshold and extrapolation method should be shown.

We thank the reviewer for pointing this out. Time-to-progression necessarily changes with the predefined colony-area threshold by construction. We therefore interpret the reviewer's request as asking whether the TTP-based optimum, relative schedule ordering, and inferred boundary are robust to reasonable changes in this threshold. In the revised analysis, we repeat the main TTP-based schedule comparison across a range of progression thresholds to demonstrate robustness (Supplementary Fig. 9).

We have also clarified the correction procedure for out-of-frame growth in the Methods and an the new Supplementary Fig. 10. In the original manuscript, this was described broadly as extrapolation beyond the imaging field of view. In practice, the procedure is not an extrapolation but an interpolation between the last trusted time-lapse frame and a lower-magnification endpoint image. The revised Methods now explain this process more carefully and describe how the last trusted frame, endpoint image, and interpolated colony or clone areas are defined.

Finally, we have added an uncertainty analysis for the interpolation. This analysis quantifies how sensitive TTP and resistant-fraction estimates are to the area reconstruction procedure. We now present these results in the supplement and refer to them from the main text so that readers can assess whether the central conclusions depend on a particular threshold or reconstruction choice. See R1.C02 for the associated changes in the revised manuscript.

R2.C06 - Copyediting and Abstract Formatting

> 3. The manuscript and supplement also need careful copyediting (e.g., the abstract explicitly states "\textit{in silico}", suggesting a lack of proofreading).

Thank you for catching this formatting error. We have corrected the abstract.

Response to the Reviewers

Appold and Citak et al.

We thank the reviewers for supporting the publication of our manuscript. Their final comments helped us to clarify the last remaining questions, which we address point-by-point below.

Reviewer 1

> The authors have responded thoughtfully to the previous reviews, and the manuscript is much improved. The new experiments, statistical analyses, colony-based fitness controls, and model extensions all strengthen the work. I also appreciate the more careful distinction between the sharp boundary seen in the model and the somewhat softer experimental crossover. Overall, the paper is in very good shape, and I would be happy to support publication after a few minor revisions.

We thank Reviewer 1 for the positive assessment of our revisions, the support to for publication of our manuscript and for identifing a few places that still need a more careful framing.

R1.C01 - Phase Transition Terminology

> I would still tread lightly around "dynamic bifurcation" and "phase transition." The new analyses are suggestive and interesting, but in a finite, stochastic, expanding system they may not quite establish a formal bifurcation. "Phase-transition-like" or "sharp stochastic transition between trajectory classes" seems safer.

We agree with the reviewer that, while suggestive, our analyis my not be enough to establish a formal bifurcation. To avoid overclaiming, we shifted the detailed bifurcation analysis into the SI while summarizing our findings more cautiously in the main text. We now cleary mention that the results are consistent with the notion of a dynamic bifurcation, but are not sufficient proof.

R1.C02 - Front-Growth Ratio

> The front-growth ratio is a useful organizing idea, but gamma front star could be defined a little more clearly. My reading is that this is a system-dependent threshold that captures a potentially general ecological principle, rather than a universal constant. It would help to say that plainly.

We agree that gamma front star should be cleary defined as a system dependent threshold, which we now do in the revised manuscript.

R1.C03 - Model-based Extensions vs Experiment

> The cytotoxic-treatment and spatial-gradient simulations are nice additions. Please keep a bright line between these model-based extensions and what has been directly demonstrated experimentally.

We concur and address this concern by introducing both, the cyctotoxic-treatment and spatial-gradiant results, cleary as results obtained from the digital twin.

R1.C04 - Adaptive Treatment Result Classification

> The adaptive-treatment result is fun and worth highlighting, but it should be introduced clearly as a proof-of-principle heuristic rather than an optimized control strategy.

We thank the reviewer for sharing our enthusiasm about the adpative-treatment result and now clearly introduce the stratevy as being a proof-of-principle heuristic and starting point for future studies rather than an already validated optimized control strategy.

R1.C05 - Experimental Sweet Spot vs Simulated Sharp Boundary

> The rebuttal does a good job distinguishing between experimentally locating the approximate sweet spot and experimentally proving the sharpness of the simulated boundary. I would carry that same caution consistently through the manuscript.

We agree. Throughout the entire manuscript we now make sure to use less ambigoues wording such as changing 'transition' to 'therapeutic sweet spot at the transition'.

R1.C06 - Copyediting

> One final proofreading pass would be worthwhile to catch the remaining typographical and notation gremlins.

We subject the final manuscript to another proofreading pass.

Reviewer 2

> This revised manuscript reports an experimental and computational study on how drug resistance evolves in spatially structured populations. As described in the original submission, the study uses a yeast colony model in which resistance can be tracked via an irreversible fluorescent switch and combines these experiments with a spatial computational model to study how intermittent treatment alters resource availability and resistant-clone escape. The manuscript is substantially improved. The paper now more clearly separates model calibration and testing, adds uncertainty analyses for fitted parameters and the inferred schedule ridge, expands the experimental analysis of the intermittent-treatment optimum, qualifies the phase-transition language, adds robustness analyses for time-to-progression, and makes the translational discussion more cautious. In my view, this paper is ready for publication after minor editorial revisions.

We thank Reviewer 2 for his consice summary of our manuscript and approving of the revisions we performed. We are especially thankfull of the assessment that the manuscript is publication ready after some minor editorial revisions which we are happy to implement.

Main

R2.C01 - Calibration and Validation

> Calibration and validation concerns have been addressed. The revision removed the pulse-duration-dependent breakout probabilities from the fitting procedure, and this version now estimates the cell-per-deme scaling from independent high-magnification images. It also reports parameter uncertainty, propagates this uncertainty through the schedule sweep, and adds leave-

> one-out cross-validation. The digital twin remains best interpreted as a mechanistic, explanatory model rather than a quantitative validator, because some fitted quantities remain closely related to the main biological dynamics. But the revised manuscript now states this limitation appropriately and tempers the claims about the exact prediction of the schedule optimum.

We are happy that our revisions adequatly addressed this concern of the reviewer.

R2.C02 - Treatment Sweet Spot

> The experimental support for the treatment sweet spot is stronger and more appropriately framed. The revision has an increased sample size for the 6.5 h/18 h schedule, new comparisons around the optimum, reports effect sizes and confidence intervals, and adds experiments varying tau_off. These additions do not establish a globally sharp optimum across all schedule space, but the manuscript no longer claims that they do. The current data support the more nuanced claim that the tested schedules show a local optimum and a qualitative switch from confinement-dominated to escape-dominated dynamics.

We are happy that our revisions adequatly addressed this concern of the reviewer.

R2.C03 - Phase Transiton Terminology

> The phase-transition language is more acceptable. The manuscript no longer presents the result simply as an "effective phase transition," but instead uses "dynamic phase-transition-like boundary" and explains that the analogy is to trajectory classes rather than to an equilibrium thermodynamic transition. The paper also defines the resistant fraction as an order-parameter-like observable and demonstrates its robustness to uncertainty in fitted parameters and to progression-threshold choice. This revision addresses a central concern, although readers should still understand the experimental evidence as supporting a sharp crossover rather than proving a formal bifurcation.

We are happy that our revisions adequatly addressed this concern of the reviewer and agree that it should be made clear for the reader that the experimental evidence does not proof a formal bifurcation. In the revised manuscript we strenghten this distinction further (see R1.C05).

R2.C04 - Translational Framing

> The translational framing is now more cautious. The revised Abstract and Discussion distinguish the general mechanism from direct tumor relevance, and the paper now notes limitations related to 2D geometry, reversible growth arrest, lack of cell killing in the main assay, drug penetration, and more complex tumor or biofilm structure. The added cytotoxic-treatment and spatial-treatment simulations are useful, and the paper correctly presents them as computational extensions rather than validation in tumor-like systems.

We are happy that our revisions adequatly addressed this concern of the reviewer.

Minor

R2.C05 - Fig. 5 Statistics

> The revised Figure 5 statistics are much clearer, with schedule-to-schedule comparisons, effect sizes, and confidence intervals. I have no further major concerns about this point.

We are happy that our revisions adequatly addressed this concern of the reviewer.

R2.C06 - Robustness to Threshold

> The robustness of time-to-progression to the progression threshold and out-of-frame interpolation is now shown in the supplement and described in the Methods. This addresses the previous concern.

We are happy that our revisions adequatly addressed this concern of the reviewer.

R2.C07 - Copyediting

> The manuscript still needs copyediting. There are still a few small issues, such as missing spaces after periods (e.g., "pulse.Motivated" and "conclusions.As") and some incorrect wording ("this data"). These should be straightforward to fix.

We thank the reviewer for catching these copyediting errors and submit the manuscript another proofreading pass to catch the last of these mistakes.
